# Dual compartment lipid carriers hijack exocytosis to empower natural killer cells against solid tumours

**DOI:** 10.1038/s41467-026-75852-6

**Published:** 2026-07-21

**Authors:** Shixin Chen, Zhenxuan Shao, Yijia Zhou, Yucheng Xue, Xupeng Chai, Lingxiao Jin, Haochen Mou, Xiao Ma, Teng Ma, Liang Chen, Xiayu Hu, Kelei Wang, Senxu Lu, Fangqian Wang, Shenzhi Zhao, Minjun Yao, Chengcheng Yu, Junjie Pan, Xiaohua Yu, Zhaoming Ye, Binghao Li

**Affiliations:** 1 Department of Orthopedic Surgery, The Second Affiliated Hospital, Zhejiang University School of Medicine, Hangzhou, China; 2 Orthopedics Research Institute of Zhejiang University, Hangzhou, China; 3 Key Laboratory of Motor System Disease Research and Precision Therapy of Zhejiang Province, Hangzhou, China; 4 Clinical Research Center of Motor System Disease of Zhejiang Province, Hangzhou, China; 5 Department of Obstetrics and Gynecology, The First Affiliated Hospital, Sun Yat-sen University, Guangzhou, China; 6 Guangdong Provincial Clinical Research Center for Obstetrical and Gynecological Diseases, Guangzhou, China

**Keywords:** Nanobiotechnology, Cytological techniques, Cancer immunotherapy

## Abstract

Solid tumours resist adoptive cell therapies through lactate driven immunosuppression, which depletes intracellular nicotinamide adenine dinucleotide, suppresses interferon gamma production in T cells, and reprogrammes macrophages. Here we show that a dual compartment lipid carrier system hijacks exocytosis for spatiotemporal metabolic reprogramming. Lipid nanoparticles deliver nicotinamide mononucleotide to restore intracellular nicotinamide adenine dinucleotide, while endoplasmic reticulum targeted carriers exploit the endoplasmic reticulum to Golgi pathway to achieve trafficking directed exocytic dichloroacetate export, enabling precise lactate depletion without systemic toxicity. This integrated approach rewires the metabolic landscape, extends natural killer cell persistence and reactivates cytolytic function. Endoplasmic reticulum engineered natural killer cells achieve potent tumour suppression through spatiotemporal metabolic reprogramming, a platform strategy that also extends to conventional T cell and macrophage therapies for enhanced therapeutic efficacy across adoptive cell systems. Our work establishes exocytosis co option as a strategy to empower cellular therapies and improve antitumour efficacy against lactate rich solid cancers.

## Abstract (web summary)

Solid tumors resist adoptive cell therapies through lactate-driven immunosuppression. Here, this group engineers a dual-compartment nanocarrier system that hijacks exocytosis for spatiotemporal metabolic reprogramming, thereby further extending NK-cell persistence and reactivating cytolytic function.

## Introduction

Cancer immunotherapy has advanced significantly in recent decades, with adoptive cell therapy (ACT) emerging as a promising approach, including tumour-infiltrating lymphocytes, T-cell-receptor-engineered T cells and chimeric antigen receptor NK cells^1,2^, which can achieve complete remission rates of 70–90% in select hematological malignancies^3^. By contrast, ACT efficacy in solid tumours is profoundly limited by multifaceted immunosuppression driven by tumour-derived lactate—a hallmark metabolic waste product that orchestrates immune dysfunction in the TME^4–7^. This lactate-driven suppression depletes NAD⁺ in NK cells by transcriptionally repressing NAMPT^8^, blocks NFAT-driven IFN-γ in CD8⁺ T cells^9,10^, and stabilizes tumour-promoting M2-like macrophages through GPR132 signaling and histone lactylation^4,11–13^, culminating in an immunosuppressive, pro-angiogenic milieu that accelerates tumour growth, metastasis and therapeutic resistance. In response, dichloroacetate (DCA) has been used to effectively reduce lactate production but induces severe peripheral neuropathy at therapeutic doses, limiting its clinical application^14–16^. Conversely, supplementation with nicotinamide mononucleotide (NMN) temporarily replenishes NAD+ pools in NK cells, but their function rapidly deteriorates as persistent lactate re-suppresses NAMPT^8,17^. This critical conundrum—wherein neither NAD⁺ preservation alone in the presence of lactate overload nor lactate clearance without concurrent NAD⁺ support restores immunometabolic function—reveals a pivotal knowledge gap: the development of integrated strategies capable of simultaneously shielding cellular metabolic machinery while reprogramming the immunosuppressive lactate milieu, all without inducing dose-limiting toxicity.

While endocytosis remains a dominant pathway for drug delivery^18,19^, exploiting exocytosis—particularly the ER-Golgi secretory route—for controlled therapeutic release has been largely overlooked^18,20–22^. Endocytosis effectively internalizes cargo but often subjects it to lysosomal degradation and lacks spatiotemporal precision upon release^23,24^. Conversely, regulated exocytosis offers a compelling^25^, yet underutilized, alternative: it enables secretory-pathway-dependent outward cargo export at the cell surface, bypassing lysosomal degradation and providing micrometre-scale spatial control. Here, spatiotemporal precision refers to ER–Golgi-guided subcellular export from engineered immune cells and its temporal alignment with NK-cell effector activity. For instance, Qiu et al. demonstrated this principle by using ER-hybrid lipid nanoparticles (ER-LNPs) to co-opt the secretory pathway^26,27^. This pathway underpins critical biological functions like neurotransmission and immune effector responses, highlighting its potential for delivering labile or toxic agents exactly when and where needed. Crucially, combining both pathways provides functional advantages: endocytosis facilitates initial cellular uptake of engineered carriers, while subsequent hijacking of the ER-Golgi-exocytosis axis ensures protected transit, precise vesicular loading, and ultimately, localized, trafficking-directed cargo export during immune-cell activation^28,29^. Building on this, we propose ER-LNPs as programmable intracellular carrier modules within various therapeutic cells: leveraging endocytosis for entry and the ER-Golgi-exocytosis cascade for efficient, spatially-restricted cargo export toward tumours, precisely upon target engagement.

**Fig. 1:**
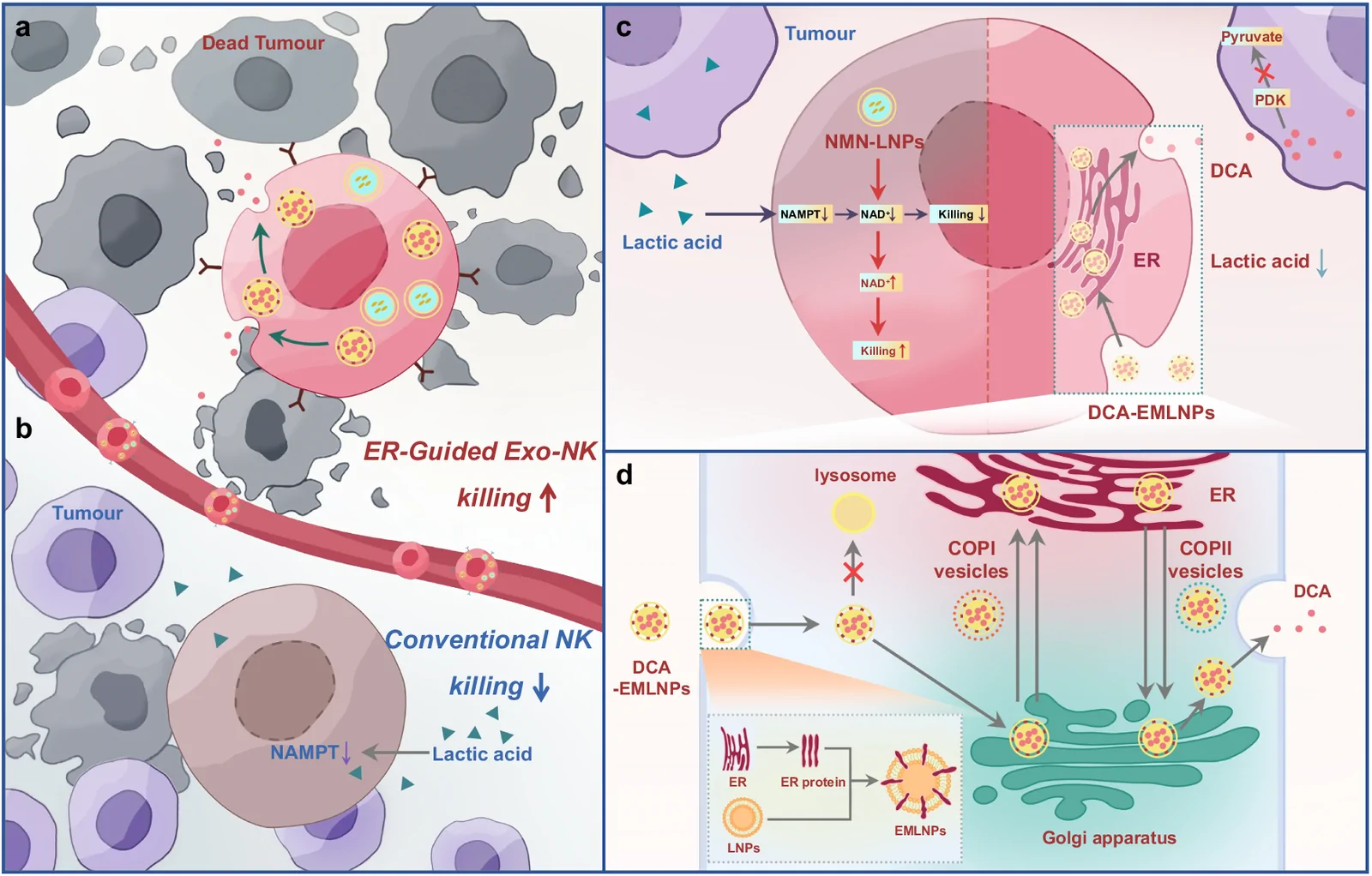
ER-guided Exo-NK design and mechanism of spatiotemporal metabolic reprogramming. **a** Schematic of the ER-Guided Exo-NK platform, incorporating cytosolic nicotinamide mononucleotide-loaded lipid nanoparticles (NMN-LNPs), endoplasmic-reticulum-hybrid dichloroacetate-loaded lipid nanoparticles (DCA-EMLNPs) and a tumour-homing peptide. **b** Conventional natural killer (NK) cells are suppressed in lactate-rich tumours through nicotinamide phosphoribosyltransferase (NAMPT) inhibition, NAD⁺ depletion and impaired effector function. **c** In ER-Guided Exo-NK cells, NMN-LNPs replenish NAD⁺, whereas DCA-EMLNPs co-opt ER-Golgi secretory trafficking to release DCA locally at the tumour-immune-cell interface. DCA inhibits pyruvate dehydrogenase kinase (PDK), restores pyruvate dehydrogenase (PDH)-dependent pyruvate oxidation and reduces lactate production in neighbouring tumour cells. **d** DCA-EMLNP trafficking model showing endocytic entry, lysosome bypass, coat protein complex I (COPI)-mediated retrograde transport to the ER, coat protein complex II (COPII)-mediated Golgi export, and secretory cargo release.

In this work, we engineer a dual-compartment system to address the dual metabolic barriers of intracellular NAD⁺ depletion and extracellular lactate accumulation that cripple NK cell therapy. Lipid nanoparticles deliver NMN to rescue intracellular NAD⁺ reserves, while ER-hybrid lipid nanoparticles encapsulating DCA co-opt the ER-Golgi secretory axis to achieve trafficking-guided outward DCA export toward the tumour-facing pericellular compartment (Fig. 1). In this study, spatiotemporal metabolic reprogramming refers to the coordinated restoration of NK-cell intracellular NAD⁺ metabolism and ER–Golgi-guided local suppression of the tumour lactate-producing pathway. We exploit the biological principle that endoplasmic-reticulum-tagged vesicles merge into the ER-Golgi secretory pathway for regulated exocytosis, thereby creating a biologically gated internalize-and-release circuit that rewires tumour metabolism, extends NK persistence and improves control of solid tumours. By concurrently safeguarding intracellular NAD⁺ and depleting pericellular lactate, our dual-compartment platform restores immunometabolic competence and re-establishes NK efficacy against refractory solid tumours.

## Results

### Lactate drives NK cell dysfunction in the osteosarcoma microenvironment

**Fig. 2:**
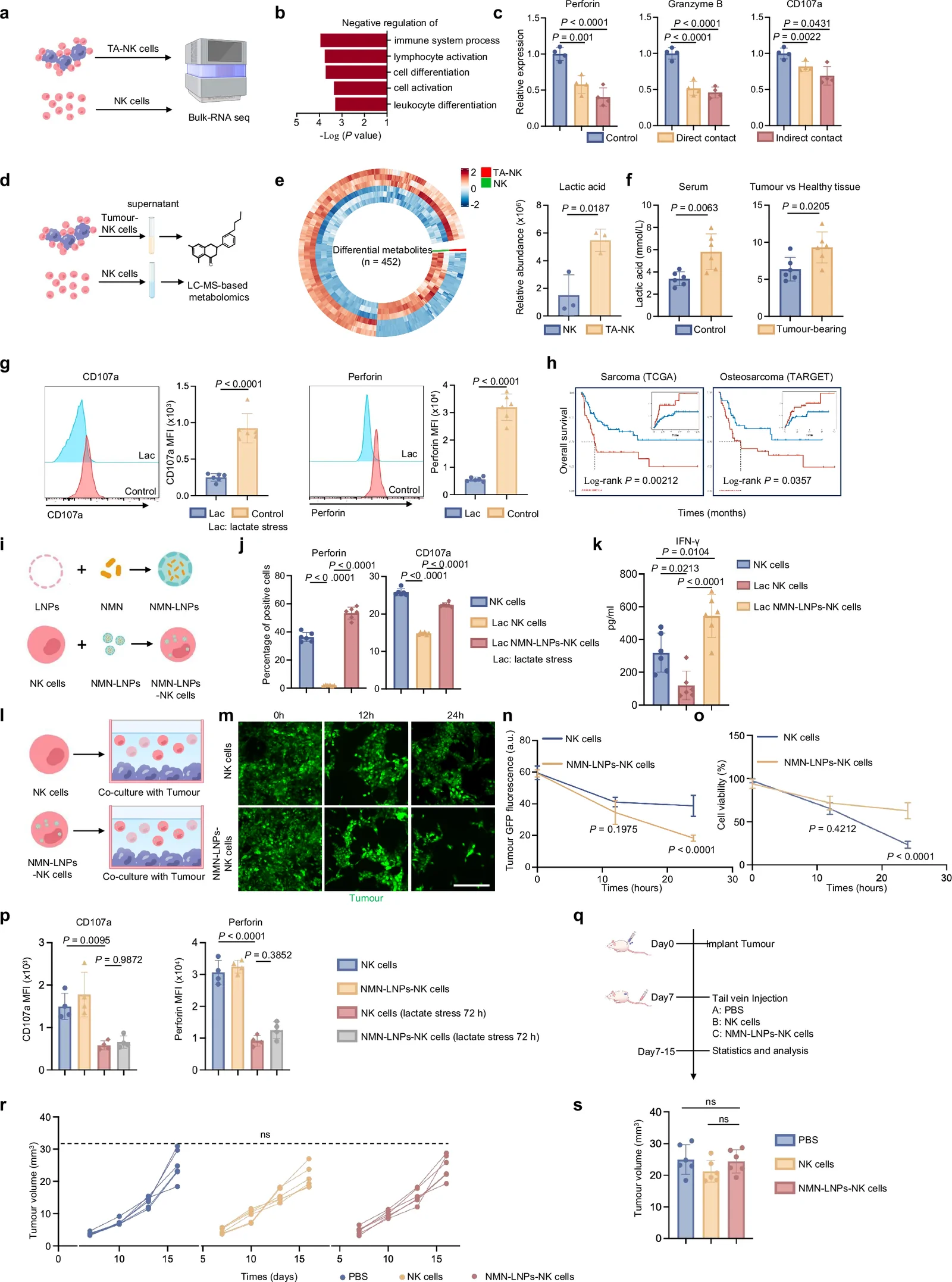
NMN-LNPs transiently rescue lactate-impaired NK-cell function. **a** RNA-seq design for NK cells alone or with GFP-HOS cells; *n* = 3 independent NK-cell cultures per group. **b** GO enrichment; one-sided hypergeometric test with Benjamini–Hochberg FDR correction. **c** Perforin, granzyme B and CD107a after HOS contact or conditioned medium; *n* = 4 independent NK-cell cultures per group, mean ± SD; one-way ANOVA, Dunnett versus Control; *P* values: perforin, 0.001 and <0.0001; granzyme B, both <0.0001; CD107a, 0.0431 and 0.0022. **d**, **e** LC–MS metabolomics of supernatants; *n* = 3 independent cultures; lactic acid, unpaired two-tailed *t*-test. **f** Serum and tibial HOS-tissue lactate; *n* = 6 mice per group, mean ± SD; unpaired two-tailed *t*-test. **g** CD107a and perforin after lactate challenge; *n* = 6 independent NK-cell cultures per group, mean ± SD; unpaired two-tailed *t*-test; both *P* < 0.0001. **h** Kaplan–Meier analysis by LDHA expression in TCGA-SARC and TARGET-OS cohorts; *n* represents patients: TCGA-SARC, *n* = 152; TARGET-OS, *n* = 95; log-rank test. **i** NMN-LNP schematic. **j**, **k** Effector markers after lactate stress with or without NMN-LNPs; *n* = 6 independent NK-cell cultures per group, mean ± SD; one-way ANOVA, Tukey. **l**, **m** Cytotoxicity assay and GFP-HOS micrographs; scale bar, 100 µm. **n** Residual GFP fluorescence; *n* = 4 independent NK-HOS co-cultures per group, mean ± SD, fields averaged per co-culture; two-way repeated-measures ANOVA, Sidak; *P* = 0.1975 at 12 h and *P* < 0.0001 at 24 h. **o** CCK-8 viability; *n* = 4 independent NK-HOS co-cultures per group per time point, mean ± SD; two-way repeated-measures ANOVA, Sidak; *P* = 0.4212 at 12 h and *P* < 0.0001 at 24 h. **p** Perforin and CD107a MFI at 0 and 72 h; *n* = 4 independent NK-cell cultures per group per time point, mean ± SD; two-way ANOVA, Sidak; *P* values: CD107a, 0.0095 and 0.9872; perforin, <0.0001 and 0.3852. **q**–**s** In vivo timeline, tumour growth and endpoint volume; *n* = 6 mice per group, mean ± SD for **r**, **s**; two-way repeated-measures ANOVA, Sidak, for **r** and one-way ANOVA, Tukey, for **s**; ns not significant. Source data are provided as a Source data file.

Adoptive NK cell therapy faces particular challenges in solid tumours like osteosarcoma, where the immunosuppressive tumour microenvironment (TME) impairs effector function. As highlighted in the introduction, lactate accumulation is a key mechanism of TME-mediated immunosuppression, linked to NAD⁺ depletion and functional silencing in immune cells. To define how the osteosarcoma milieu specifically disables NK cells and identify dominant suppressive factors, we profiled NK cell responses in co-culture models and aligned these with in vivo and clinical readouts. RNA-seq comparing NK cells alone with HOS co-culture identified 12,006 differentially expressed genes (FDR < 0.05). Gene set analysis revealed significant enrichment for terms linked to negative regulation of lymphocyte activation and differentiation, collectively defining a tumour-associated NK (TA-NK) programme (Fig. 2a, b). NK-cell impairment was reproduced by tumour-conditioned medium, with perforin, granzyme B and CD107a reduced to a similar extent as in direct co-culture, indicating that soluble mediators are sufficient (Fig. 2c). Untargeted LC–MS of supernatants revealed 452 altered metabolites (VIP > 1, *P* < 0.05), among which lactate was selectively increased (*P* = 0.0187; Fig. 2d, e and Supplementary Fig. 1a–d). In an orthotopic tibial model, tumour-bearing NSG mice exhibited higher lactate in serum (*P* = 0.0063) and at tumour sites (*P* = 0.0205) than controls (Fig. 2f). Exogenous lactate directly suppressed perforin and CD107a in activated NK cells in vitro (both *P* < 0.0001; Fig. 2g). Clinically, elevated LDH expression—marking increased lactate metabolism—associated with poorer overall survival in TCGA-SARC (*n* = 152) and TARGET-OS (*n* = 95) cohorts (log-rank *P* < 0.05; Fig. 2h).

### Liposomal NMN provides only transient rescue of lactate-impaired NK cell function

Given the link between lactate, NAD⁺ depletion, and functional impairment, we next tested a proximate countermeasure: transient replenishment of intracellular NAD⁺ using liposomally encapsulated nicotinamide mononucleotide (NMN-LNPs). NMN was efficiently encapsulated into ~100 nm LNPs via low-temperature microfluidic mixing, shifting the *ζ*-potential from −5 mV to −31 mV; FT-IR confirmed loading (Supplementary Fig. 2a–c). Human NK cells internalized NMN-LNPs within 2 h (Fig. 2i). To emulate the clinically relevant ex vivo engineering workflow (brief loading followed by extensive washing before infusion), we compared free NMN versus NMN-LNPs under a pulse-and-wash paradigm and tracked intracellular NAD⁺/NADH dynamics over time in lactate-stressed NK cells. Notably, free NMN produced only a transient effect and was not significantly different from lactate-stressed NK controls across the time course (ns), whereas NMN-LNP treatment maintained a markedly higher NAD⁺/NADH ratio from 6 to 48 h (*P* = 0.001 versus NK controls; *P* = 0.0109 versus free NMN; Supplementary Fig. 2d), indicating superior intracellular delivery and durable redox restoration. We next asked whether this NMN-LNP-mediated redox restoration translated into functional recovery of NK-cell effector programmes. Lactate exposure reduced the intracellular NAD⁺/NADH ratio, whereas NMN-LNP treatment restored this redox balance (Supplementary Fig. 2e). We then evaluated three representative NK-cell effector readouts: CD107a as a marker of degranulation and cytotoxic granule exocytosis, perforin as a cytotoxic effector molecule, and IFN-γ as a cytokine-producing effector readout. Lactate stress reduced CD107a, perforin, and IFN-γ production, indicating impaired degranulation, cytotoxic effector capacity, and cytokine function. NMN-LNP preloading restored CD107a and perforin expression (Fig. 2j), increased IFN-γ secretion (Fig. 2k), and raised the percentage of IFN-γ⁺ NK cells (Supplementary Fig. 3b, c). Moreover, the intracellular NAD⁺/NADH ratio positively correlated with both CD107a MFI and IFN-γ⁺ NK-cell frequency (Supplementary Figs. 2g and 3d), supporting a functional linkage between NMN-LNP-mediated redox restoration and NK-cell effector competence. We therefore pretreated NK cells with NMN-LNPs, removed unbound particles, and evaluated their cytotoxicity against tumour cells in co-culture (Fig. 2l). Under lactate stress, NMN-LNP-NK cells initially maintained perforin and CD107a at control-like levels (Fig. 2j). Time-point fluorescence imaging showed no difference in cytotoxicity at 12 h (*P* = 0.1975) but a significant advantage for NMN-LNP-treated NK cells by 24 h (*P* < 0.0001; Fig. 2m, n). This transient enhancement in cytotoxicity was corroborated by CCK-8 viability assays at the corresponding time points (Fig. 2o). To define the molecular basis underlying the improved fitness and cytotoxicity observed 24 h after NMN-LNP conditioning, we next profiled mitochondrial oxidative metabolism and global transcriptional programmes in NMN-LNP-treated NK cells. Seahorse flux analysis revealed a consistent elevation of oxygen consumption rate (OCR) across the mitochondrial stress test in NMN-LNP-primed NK cells compared with untreated controls (Supplementary Fig. 3e). Quantification of key bioenergetic parameters showed significantly increased maximal respiration, spare respiratory capacity, and basal respiration, together with an increase in ATP-linked respiration (Supplementary Fig. 3f), indicating that NMN-LNP treatment enhances oxidative phosphorylation capacity and respiratory reserve under these conditions. In parallel, bulk RNA-seq demonstrated broad transcriptional remodeling following NMN-LNP conditioning (Supplementary Fig. 3g). Gene ontology enrichment of differentially expressed genes highlighted upregulation of signaling- and activation-associated pathways, including peptidyl-tyrosine phosphorylation, positive regulation of peptidyl-tyrosine phosphorylation, calcium ion import across the plasma membrane, positive regulation of protein tyrosine kinase activity, and positive regulation of tyrosine phosphorylation of STAT protein, together with cellular oxidant detoxification (Supplementary Fig. 3h). However, by 72 h, both cytotoxicity and effector marker expression (Fig. 2p) in NMN-LNP-treated cells had converged back to the levels of untreated, lactate-impaired cells. In vivo, a single intravenous infusion of unmodified or NMN-loaded NK cells into mice bearing established subcutaneous HOS tumours did not improve tumour control versus untreated cohorts (longitudinal volumes and end-point tumour measurements by callipers; Fig. 2q–s). Consistent with the transient effects observed in vitro, this NMN supplementation strategy failed to translate into sustained therapeutic benefit in vivo.

Together, Fig. 2 establishes lactate as a dominant soluble mediator suppressing key NK cell antitumour functions and demonstrates that liposomal NMN delivery, while providing a transient boost in NAD⁺-dependent cytotoxicity and effector marker expression, fails to confer durable therapeutic efficacy. This limitation is attributed to the inability of NAD⁺ replenishment alone to counteract the continued immunosuppressive pressure exerted by high extracellular lactate accumulation within the TME. These findings strongly motivate the development of alternative strategies that directly target and deplete tumour-derived lactate to achieve sustained metabolic reprogramming and immune cell function.

### ER-hybrid nanocarriers leverage secretory exocytosis for spatiotemporally controlled delivery

**Fig. 3:**
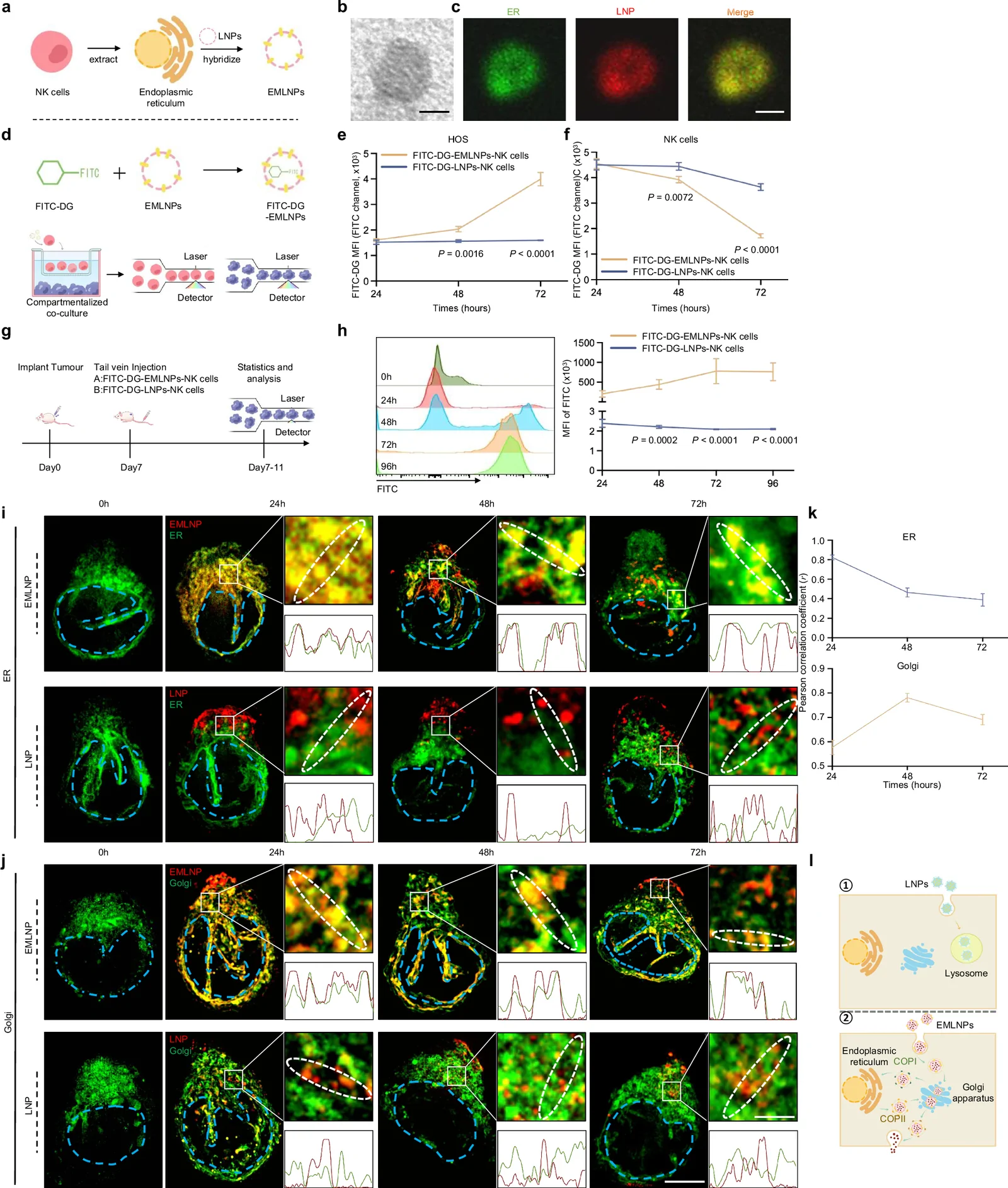
ER-hybrid lipid nanoparticles mediate ER-Golgi-guided payload export. **a** EMLNP preparation by fusing ER membranes from human NK cells with synthetic LNPs. **b** Transmission electron microscopy showing ~100 nm spherical EMLNPs; scale bar, 50 nm. **c** Confocal images showing overlap of green-labelled ER membranes and red-labelled lipids in EMLNPs; scale bar, 50 nm. **d** Transwell assay using donor NK cells loaded with FITC-dextran (FITC-DG)-EMLNPs or FITC-DG-LNPs and recipient HOS cells. **e**, **f** FITC MFI in recipient HOS cells and donor NK cells over time; *n* = 4 independent Transwell co-cultures per group, mean ± SD; two-way ANOVA, Sidak. Exact *P* values for FITC-DG-EMLNPs-NK cells versus FITC-DG-LNPs-NK cells are: **e**, *P* = 0.0016 at 48 h and *P* < 0.0001 at 72 h; **f**, *P* = 0.0072 at 48 h and *P* < 0.0001 at 72 h. **g**, **h** In vivo FITC-DG delivery in orthotopic HOS tumours after i.v. infusion of FITC-DG-loaded NK cells; for quantification in **h**, *n* = 6 tumours per group per time point, mean ± SD; two-way ANOVA, Sidak. **i**, **j** Structured-illumination microscopy images showing PKH26-labelled nanoparticles in red and ER or Golgi markers in green at 0, 24, 48 and 72 h; scale bars, 5 µm and 0.8 µm. **k** Pearson correlation coefficient quantification of nanoparticle co-localisation with ER and Golgi markers; *n* = 4 independent NK-cell imaging experiments per group per time point, mean ± SD, with technical imaging fields averaged per experiment. **l** Model comparing lysosomal routing of conventional LNPs with ER-Golgi-associated secretory cargo export by EMLNPs. Source data are provided as a Source data file.

Conventional nanocarriers suffer from lysosomal entrapment and degradation, severely limiting therapeutic payload delivery to target sites—a critical barrier in cancer immunotherapy. To overcome this, we hypothesized that fusing endogenous organelle membranes with synthetic nanoparticles could co-opt cellular trafficking machinery for precise release. Here, we engineered endoplasmic reticulum (ER)-hybrid liposomes (EMLNPs) by fusing ER membranes isolated from human NK cells with lipid nanoparticles via controlled ultrasonication (Fig. 3a). Dynamic light scattering and TEM confirmed uniform spherical morphology (diameter ≈100 nm; Fig. 3b and Supplementary Fig. 4a), while confocal imaging demonstrated complete overlap of green-labelled ER and red-labelled lipids (Fig. 3c), indicating seamless membrane integration. Coomassie staining further verified retention of functional ER proteins (Supplementary Fig. 4b), collectively establishing a biohybrid platform capable of hijacking secretory pathways for payload delivery.

To assess whether EMLNPs enhance cell-to-cell payload delivery in solid tumours, we employed a Transwell co-culture model (HOS tumour cells and NK cells). FITC-dextran-loaded EMLNPs achieved 150.2% higher accumulation in HOS cells versus conventional LNPs at 72 h (MFI: 3995.3 vs 1596.5; *P* < 0.0001; Fig. 3e), with parallel 111.1% MFI decline in NK cells (Fig. 3f), confirming efficient intercellular transfer. This transcellular delivery translated to in vivo specificity: Orthotopic HOS models showed tumour-selective payload accumulation, with MFI increasing 362.29-fold (*P* < 0.0001; Fig. 3g, h). Structured illumination microscopy (SIM) revealed that EMLNPs strongly co-localized with ER and Golgi markers over time (Fig. 3i, j), in stark contrast to conventional LNPs, which showed minimal organelle co-localization. Two-dimensional intensity-distribution plots (Supplementary Fig. 6a, b) further illustrated the dense diagonal clustering of EMLNP pixels, consistent with strong co-localisation. Temporal analysis of Pearson correlation coefficients (PCC) uncovered a dynamic redistribution pattern: ER co-localization progressively decreased from 0.81 ± 0.03 at 24 h to 0.39 ± 0.05 by 72 h, while Golgi association peaked at 48 h (0.78 ± 0.02) before slightly declining to 0.70 ± 0.03 at 72 h (Fig. 3k). To causally test whether this ER-to-Golgi trafficking underpins the proposed exocytosis-hijacking mechanism, we quantified EMLNP cargo export using a two-readout release assay. Donor NK cells were preloaded with FITC-DG-EMLNPs and, at 24, 48 and 72 h, FITC fluorescence in the culture supernatant was measured by a plate reader, reflecting released cargo, while intracellular FITC retention in donor NK cells was quantified by flow cytometry. Pharmacological inhibition of early secretory transport with Golgicide A (GCA, 5–10 μM) significantly reduced supernatant FITC accumulation and concomitantly attenuated intracellular FITC loss from NK cells (Supplementary Fig. 6c–e). Consistently, ARF1 knockdown (COPI) suppressed FITC-DG export and preserved intracellular FITC (Supplementary Fig. 6f–h). Blocking ER export with FLI-06 (5–10 μM) similarly impaired FITC-DG release (Supplementary Fig. 6i–k). Moreover, SEC23B knockdown (COPII) markedly inhibited FITC-DG export and prevented the reciprocal decline of intracellular FITC in NK cells (Supplementary Fig. 6l–n). This biphasic trajectory, characterized by initial COPI-mediated retrograde transport to the ER followed by COPII vesicle packaging, supports a model of secretory-pathway-dependent cargo export (Fig. 3l). This ER-Golgi secretory exploitation provides two functional advantages: payload protection and spatiotemporally constrained release at tumour-NK contact sites. The quantified organelle retention dynamics and payload protection data collectively validate EMLNPs as a nanoplatform that co-opts endogenous secretory machinery for precision immunometabolic modulation.

### Dual-compartment nanoparticle design combining ER-targeted lactate depletion and cytosolic NK metabolic support rescues lactate-impaired antitumour cytotoxicity

**Fig. 4:**
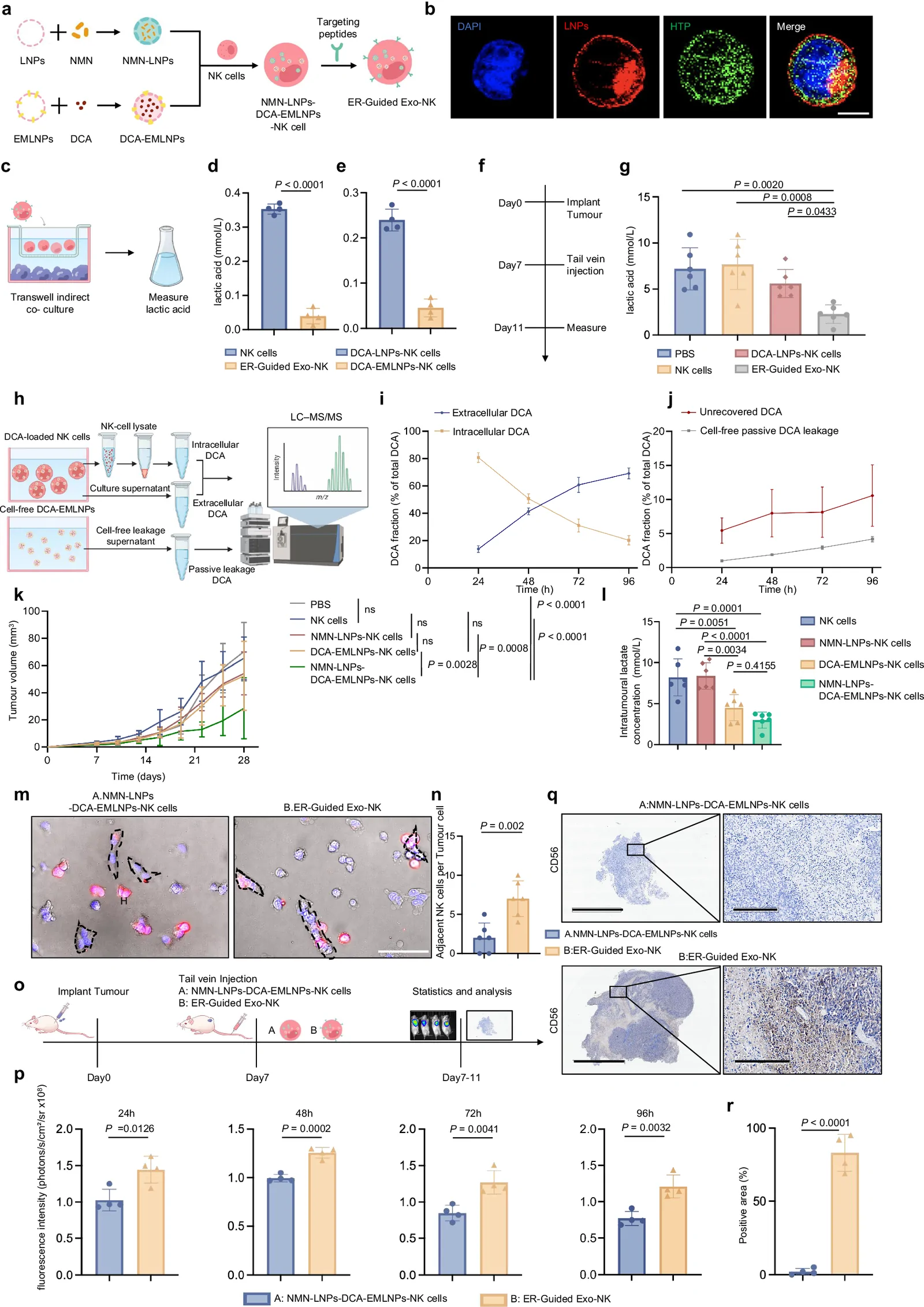
Dual-compartment NK-cell engineering restores cytotoxicity under lactate stress. **a** Co-loading strategy generating ER-Guided Exo-NK cells with NMN-LNPs, DCA-EMLNPs and HOS-targeting peptide (HTP). **b** Confocal validation of HTP membrane localization and intracellular LNP uptake; nuclei, blue; LNPs, red; HTP, green; scale bar, 5 µm. **c** Transwell assay for lactate suppression in HOS co-culture. **d**, **e** Lactate after ER-Guided Exo-NK treatment or NK cells bearing conventional DCA-LNPs versus DCA-EMLNPs; *n* = 4 independent Transwell co-cultures per group, mean ± SD; unpaired two-tailed *t*-test; both *P* < 0.0001. **f**, **g** In vivo timeline and tumour lactate; *n* = 6 mice per group, mean ± SD; one-way ANOVA with Dunnett’s test using ER-Guided Exo-NK as reference; *P* = 0.0020, *P* = 0.0008 and *P* = 0.0433. **h** LC–MS/MS workflow measuring intracellular DCA, extracellular DCA, passive leakage and unrecovered fraction. Created in BioRender. Chai, X. (2026) https://BioRender.com/j3zoy8s. **i**, **j** DCA fractions and passive leakage over 24–96 h; *n* = 6 independent NK-cell cultures for intracellular, extracellular and unrecovered DCA fractions, and *n* = 6 independent cell-free DCA-EMLNP incubations for passive leakage; mean ± SD. **k**, **l** Tumour growth and tumour lactate after PBS, NK, NMN-LNPs-NK, DCA-EMLNPs-NK or NMN-LNPs-DCA-EMLNPs-NK treatment; *n* = 6 mice per group, mean ± SD; two-way repeated-measures ANOVA with Sidak’s test for k and one-way ANOVA with Tukey’s test for l; ns, not significant. **m**, **n** Spatial association of PKH26-labelled NK-cell products with HOS cells; scale bar, 100 µm; *n* = 6 independent NK-HOS co-cultures per group, mean ± SD, fields averaged per co-culture; unpaired two-tailed *t*-test. **o**, **p** In vivo tracking of DiR-labelled NK-cell products; A, NMN-LNPs-DCA-EMLNPs-NK cells; B, ER-Guided Exo-NK cells; *n* = 4 mice per group measured longitudinally, mean ± SD; unpaired two-tailed *t*-test at each time point; *P* = 0.0126, *P* = 0.0002, *P* = 0.0041 and *P* = 0.0032 at 24, 48, 72 and 96 h. **q**, **r** CD56 immunohistochemistry and quantification; scale bars, 4 mm and 300 µm; *n* = 4 tumours from 4 mice per group, mean ± SD, fields averaged per tumour; unpaired two-tailed *t*-test; *P* < 0.0001. Source data are provided as a Source data file.

Building upon the EMLNP platform capable of hijacking secretory exocytosis for spatiotemporally controlled payload release (Fig. 3), we engineered a dual-compartment system (ER-Guided Exo-NK, Fig. 4a) to overcome the transient efficacy of NAD⁺ replenishment alone (Fig. 2) by simultaneously reviving NK cell metabolism (via cytosolic NMN-LNPs) and depleting the immunosuppressive lactate pool at its source (via ER-targeted DCA-EMLNPs released at tumour-NK interface). Purified NK cells were simultaneously co-incubated for 2 h with NMN-LNPs and DCA-EMLNPs in a one-step co-loading procedure, followed by extensive washing to remove unbound nanoparticles. In this dual-compartment design, NMN-LNPs served as the intracellular NAD⁺-supporting module, whereas DCA-EMLNPs served as the ER-guided lactate-depleting module. In tumour-conditioned settings, co-loading with DCA-EMLNPs further increased the NK-cell NAD⁺/NADH ratio compared with NMN-LNPs alone, consistent with lactate depletion alleviating ongoing NAD⁺ suppression (Supplementary Fig. 2f). ζ-Potential measurements confirmed DCA encapsulation (shift from −3 mV for empty EMLNPs to −28.1 mV after loading; Supplementary Fig. 4c). FT-IR spectroscopy showed attenuation of DCA bands at 799, 1399 and 1620 cm⁻¹ and the emergence of a 1110 cm⁻¹ band, consistent with sodium-phosphate interactions within the lipid matrix (Supplementary Fig. 4d). To enhance tumour engagement, a phage-derived targeting peptide was displayed on the NK-cell membrane. Confocal microscopy verified correct assembly: the FITC-labelled peptide remained confined to the surface, whereas Cy5.5-labelled NMN-LNPs and DCA-EMLNPs localised intracellularly, indicating efficient vesicular uptake with preserved surface targeting (Fig. 4b). Because the complete dual-compartment formulation interfaces with intracellular trafficking and the ER–Golgi secretory route, we next examined whether nanoparticle co-loading perturbed native NK-cell effector secretion. Under the same activation conditions used in the functional assays, neither DCA-EMLNP loading nor full NMN-LNP and DCA-EMLNP co-loading altered the degranulation marker CD107a or perforin expression compared with untreated NK cells or NMN-LNP-only controls (Supplementary Fig. 5a–d). These data indicate that the ER-Guided Exo-NK engineering procedure preserves endogenous NK-cell cytolytic secretory function while adding a controllable ER-guided cargo-export module. We next quantified lactate control and targeting performance. In a Transwell co-culture (ER-Guided Exo-NK in the upper chamber; HOS cells below; Fig. 4c), conditioned media from ER-Guided Exo-NK showed reduced lactate versus controls (Fig. 4d). Direct comparison demonstrated that ER-hybrid DCA-EMLNPs suppressed lactate more effectively than conventional DCA-LNPs at matched doses (Fig. 4e), consistent with ER-guided trafficking and secretory-trafficking-coupled exocytosis. In an HOS xenograft model, tail-vein administration of ER-Guided Exo-NK reduced intratumoural lactate relative to PBS, unmodified NK cells and DCA-LNPs-NK (*P* = 0.0020, *P* = 0.0008 and *P* = 0.0433, respectively; Fig. 4f, g). The failure of conventional DCA-LNPs-NK to significantly reduce lactate highlights the necessity of the ER-targeted, spatiotemporally controlled delivery mechanism for effective lactate depletion within the tumour bed.

To quantitatively define the fate of DCA after cellular loading, we established an LC–MS/MS-based mass-balance workflow to separately measure intracellular DCA, extracellular DCA and cell-free passive leakage (Fig. 4h), with representative chromatograms shown in Supplementary Fig. 16a–d. Extracellular DCA progressively increased from 13.86 ± 2.42% at 24 h to 41.31 ± 2.51% at 48 h, 60.77 ± 5.53% at 72 h and 69.13 ± 3.92% at 96 h, whereas intracellular DCA declined reciprocally from 80.70 ± 3.63% to 50.70 ± 3.56%, 31.09 ± 4.67% and 20.29 ± 3.41% over the same time points (Fig. 4i). The calculated unrecovered DCA fraction, defined as 100%—extracellular DCA fraction—intracellular DCA fraction, remained low, measuring 5.43 ± 1.87%, 7.99 ± 3.48%, 8.14 ± 3.69% and 10.58 ± 4.52% at 24, 48, 72 and 96 h, respectively (Fig. 4j). As a cell-free reference, passive leakage from DCA-EMLNPs was measured under identical incubation conditions, with extracellular DCA detected at 0.99 ± 0.12%, 1.89 ± 0.18%, 2.94 ± 0.27% and 4.18 ± 0.38% at 24, 48, 72 and 96 h (Fig. 4j and Supplementary Fig. 16b). Together, these LC–MS/MS data quantified the time-dependent extracellular accumulation and intracellular retention of DCA after DCA-EMLNP loading. To further dissect the relative contribution of the two metabolic modules, we compared matched NK-cell products loaded with NMN-LNPs alone, DCA-EMLNPs alone, or both NMN-LNPs and DCA-EMLNPs. In vivo tumour-growth analysis showed that NMN-LNPs-DCA-EMLNPs-NK cells achieved the strongest tumour control, whereas NMN-LNP-loaded NK cells and DCA-EMLNP-loaded NK cells produced only partial antitumour effects (Fig. 4k). Tumour lactate analysis further showed that DCA-containing NK-cell products markedly reduced lactate accumulation, whereas NMN-LNP-loaded NK cells alone had limited effect on tumour lactate levels, indicating that DCA was the principal lactate-depleting module (Fig. 4l). Targeting specificity was supported by microscopy: PKH26-labelled ER-Guided Exo-NK exhibited greater spatial association with HOS cells than non-targeted NK cells (Fig. 4m, n). Whole-animal imaging with DiR-labelled NK cells showed preferential tumour accumulation of ER-Guided Exo-NK that increased from 24–96 h post-injection and exceeded that of non-targeted counterparts (Fig. 4o, p). Finally, immunohistochemistry demonstrated marked infiltration of targeted NK cells into tumour tissue (≈80% positive areas) compared with ~1% in non-targeted groups (*P* < 0.0001; Fig. 4q, r). To determine whether intracellular NMN-mediated NK-cell protection and DCA-mediated lactate suppression occurred within compatible functional windows, we performed a time-aligned analysis of NK-cell tumour homing, NK-cell function, DCA export and tumour lactate reduction. Based on the tumour-homing data shown in Fig. 4o, p, ER-Guided Exo-NK cells were detectable in the tumour region from 24 h after systemic infusion and progressively accumulated from 24 to 96 h. We therefore defined 0–24 h as the NK-cell tumour-homing phase and 24–96 h as the main post-homing functional window. To clarify the temporal relationship between NK-cell functional preservation and DCA export, we used the lactate-stress-only condition as a reference for the duration of NK-cell activity when lactate was not effectively cleared. Under persistent lactate stress, ER-Guided Exo-NK cells maintained higher CD107a expression through the early post-homing period, but functional decline became evident by approximately 72 h. During the same 24–72 h overlap window, extracellular DCA export progressively increased and was largely established by 72 h (Supplementary Fig. 17f). When DCA-mediated lactate relief was included in the ER-Guided Exo-NK system, NK-cell functional activity was maintained beyond the lactate-stress-only reference window and was accompanied by progressive tumour lactate reduction (Supplementary Fig. 17g). These data indicate that NAD⁺ replenishment and lactate clearance continuously support each other during the critical immune-cell activity window, rather than presenting a mismatched or merely sequential action that could limit therapeutic efficacy. Together, pairing ER-directed DCA delivery with NMN-mediated metabolic support lowers extracellular lactate, enhances tumour localization and infiltration, and restores NK-cell antitumour cytotoxicity under lactate stress. The inability of conventional DCA-LNPs-NK to recapitulate these outcomes highlights ER-hybrid trafficking as the key determinant of effective lactate depletion at the tumour interface. This integrated, spatiotemporally controlled metabolic reprogramming strategy thus provides a platform to enhance adoptive cell therapy efficacy against lactate-rich solid tumours

### Spatiotemporal metabolic reprogramming drives potent antitumour efficacy and survival benefit

**Fig. 5:**
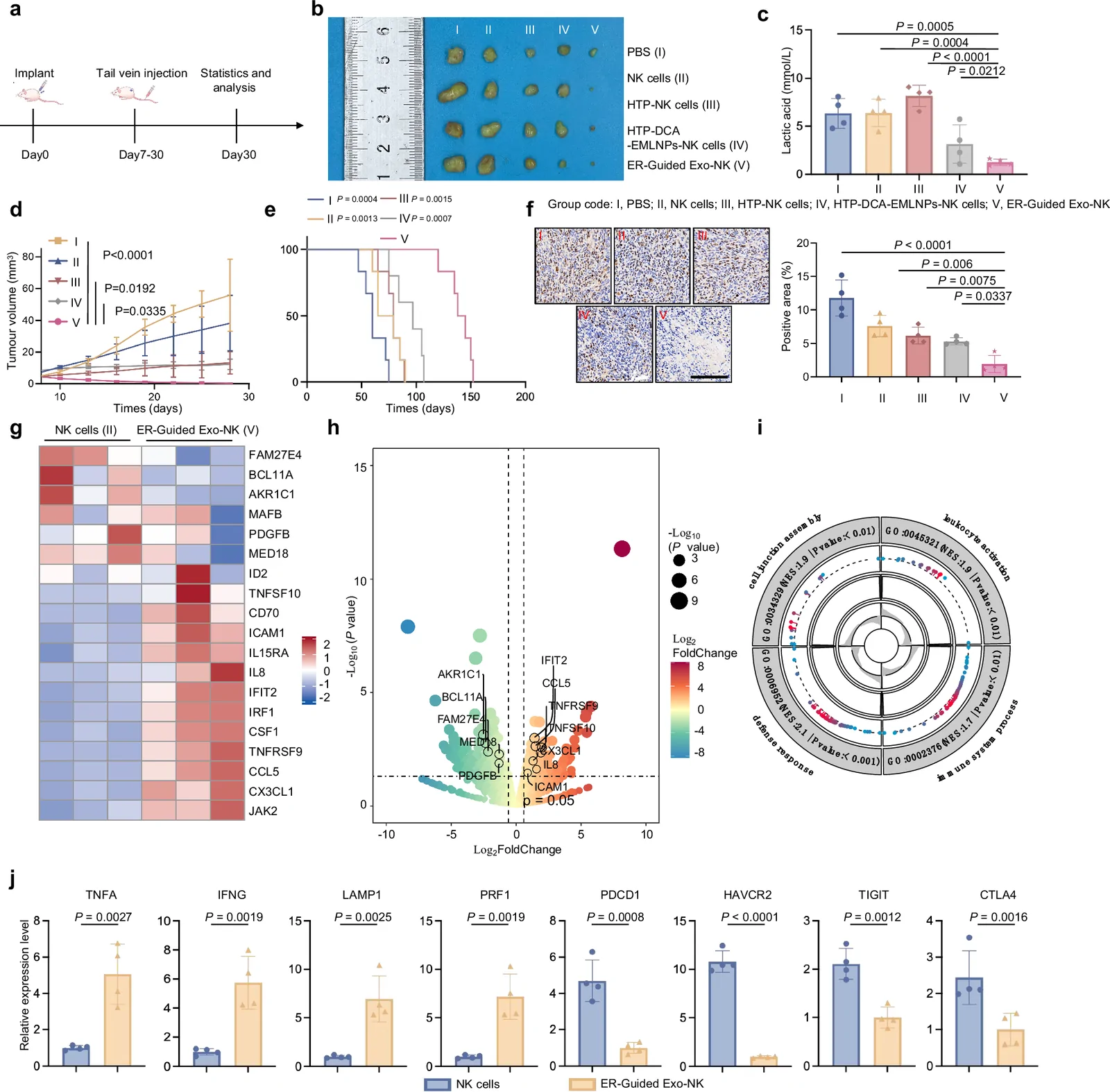
ER-Guided Exo-NK improves tumour control and survival. **a** Subcutaneous osteosarcoma treatment design and groups: PBS (I), NK cells (II), HTP-NK cells (III), HTP-DCA-EMLNPs-NK cells (IV) and ER-Guided Exo-NK cells (V). **b** Representative endpoint tumour images. **c** Intratumoural lactate at day 30; *n* = 4 mice per group, mean ± SD; one-way ANOVA, Tukey. Exact *P* values versus ER-Guided Exo-NK are *P* = 0.0005 for PBS, *P* = 0.0004 for NK cells, *P* < 0.0001 for HTP-NK cells and *P* = 0.0212 for HTP-DCA-EMLNPs-NK cells. **d** Tumour-growth curves; *n* = 4 mice per group, mean ± SD; two-way repeated-measures ANOVA, Sidak. Exact *P* values are I versus V, *P* < 0.0001; III versus V, *P* = 0.0192; and IV versus V, *P* = 0.0335. **e** Kaplan–Meier survival curves; *n* = 6 mice per group; log-rank test. **f** Ki-67 immunohistochemistry and proliferation quantification; *n* = 4 tumours from 4 mice per group, mean ± SD, with imaging fields averaged per tumour; one-way ANOVA, Tukey. Exact *P* values versus ER-Guided Exo-NK are *P* < 0.0001 for PBS, *P* = 0.006 for NK cells, *P* = 0.0075 for HTP-NK cells and *P* = 0.0337 for HTP-DCA-EMLNPs-NK cells; scale bar, 150 µm. **g**, **h** Tumour transcriptomics as heatmap and volcano plot; *n* = 3 tumours per group; edgeR two-sided exact test with Benjamini–Hochberg FDR correction. **i** GO enrichment; one-sided Fisher’s exact test with Benjamini–Hochberg FDR correction. **j** qRT-PCR validation; *n* = 4 mice per group, mean ± SD; unpaired two-tailed *t*-test for each gene. Exact *P* values are TNFA, 0.0027; IFNG, 0.0019; LAMP1, 0.0025; PRF1, 0.0019; PDCD1, 0.0008; HAVCR2, <0.0001; TIGIT, 0.0012; CTLA4, 0.0016. Source data are provided as a Source data file.

With the ER-Guided Exo-NK platform’s ability to lower lactate, enhance targeting, and restore NK function established (Fig. 4), we assessed its therapeutic efficacy in osteosarcoma models. Critically, we sought to determine if its core spatiotemporal metabolic reprogramming strategy delivers superior tumour control and survival versus controls. Therapeutic efficacy was evaluated in a subcutaneous HOS osteosarcoma model. Following tumour establishment on day 0, mice were randomized on day 7 into five treatment groups: PBS; native NK; peptide-targeted NK (HTP-NK); HTP-DCA-EMLNPs-NK; or ER-Guided Exo-NK (Fig. 5a). Across the monitoring window, ER-Guided Exo-NK achieved the greatest tumour control, being the only arm to show regression while groups I-IV continued to enlarge (Fig. 5b, d). Mechanistically, and in line with local ER-guided DCA delivery, intratumoural lactate fell only in DCA-containing regimens, with the largest reduction in the ER-Guided Exo-NK cohort (versus PBS *P* = 0.0005; versus HTP-NK 0.0001; Fig. 5c). Consistent with this lactate-depleting effect, ER-Guided Exo-NK also reduced tumour PDHA1 phosphorylation, as shown by western blotting and p-PDHA1 immunohistochemistry (Supplementary Fig. 17a–e). These data further support pathway-level suppression of the PDK-PDHA1 axis underlying lactate-producing pyruvate metabolism. This translated into a survival advantage in an independent cohort (Fig. 5e). Concordantly, Ki67 indices were lowest in ER-Guided Exo-NK tumours (Fig. 5f). As shown in the component-dissection analysis in Fig. 4k, l, matched NK-cell products loaded with both NMN-LNPs and DCA-EMLNPs achieved the strongest tumour control, whereas NMN-LNP-loaded NK cells and DCA-EMLNP-loaded NK cells produced only partial antitumour effects. Tumour lactate analysis further showed that DCA-containing NK-cell products markedly reduced lactate accumulation, whereas NMN-LNP-loaded NK cells alone had limited effect on tumour lactate levels, indicating that DCA was the principal lactate-depleting module. However, DCA alone did not fully restore NK-cell effector function. The combined NMN-LNPs-DCA-EMLNPs-NK cells produced the strongest recovery of IFN-γ production, as shown by ELISA, intracellular flow cytometry and tumour IFN-γ immunostaining (Supplementary Fig. 18a–c, h, i). Consistently, CD107a and perforin expression were most strongly restored in the combined-treatment group (Supplementary Fig. 18d–g). These findings indicate that NMN-LNPs and DCA-EMLNPs perform complementary and non-redundant functions: DCA-EMLNPs primarily remodel the lactate-rich tumour metabolic niche, whereas NMN-LNPs reinforce NK-cell intracellular metabolic fitness and effector competence. Their combination is therefore required for optimal IFN-γ recovery, cytotoxic effector restoration and tumour control. ER-Guided Exo-NK demonstrated a favourable safety profile. No significant elevation in cytokines associated with cytokine release syndrome (CRS) or markers of liver or cardiac injury was observed compared to PBS controls. Histopathological examination of major organs revealed no treatment-related abnormalities (Supplementary Figs. 7a, 11a and 14a). Mechanistically, bulk tumour transcriptome analysis revealed that ER-Guided Exo-NK treatment reprogrammed the tumour immune microenvironment, upregulating pathways critical for leukocyte recruitment (IL8, CCL5, CX3CL1, ICAM1), NK-sustaining cytokines (IL15RA), and cytotoxic/co-stimulatory pathways (TRAIL/TNFSF10, 4-1BB/TNFRSF9) (Fig. 5g, h). GO enrichment analysis confirmed coordinated immune reactivation—enriched for leukocyte adhesion, activation, and effector responses (Fig. 5i). qRT-PCR validated functional reinvigoration: elevated TNFA, IFNG, LAMP1, and PRF1 coupled to diminished exhaustion markers (PDCD1,HAVCR2, TIGIT, CTLA4). Together, these data demonstrate that the ER-Guided Exo-NK platform drives potent tumour control by integrating two complementary metabolic interventions: DCA-EMLNP-mediated lactate depletion in the tumour niche and NMN-LNP-mediated reinforcement of NK-cell metabolic fitness. This coordinated dual-module strategy restores IFN-γ production and cytotoxic effector function, reprogrammes the immunosuppressive TME, and extends survival with a favourable safety profile.

### Translational validation of ER-Guided Exo-NK therapy in patient-derived xenograft models

**Fig. 6:**
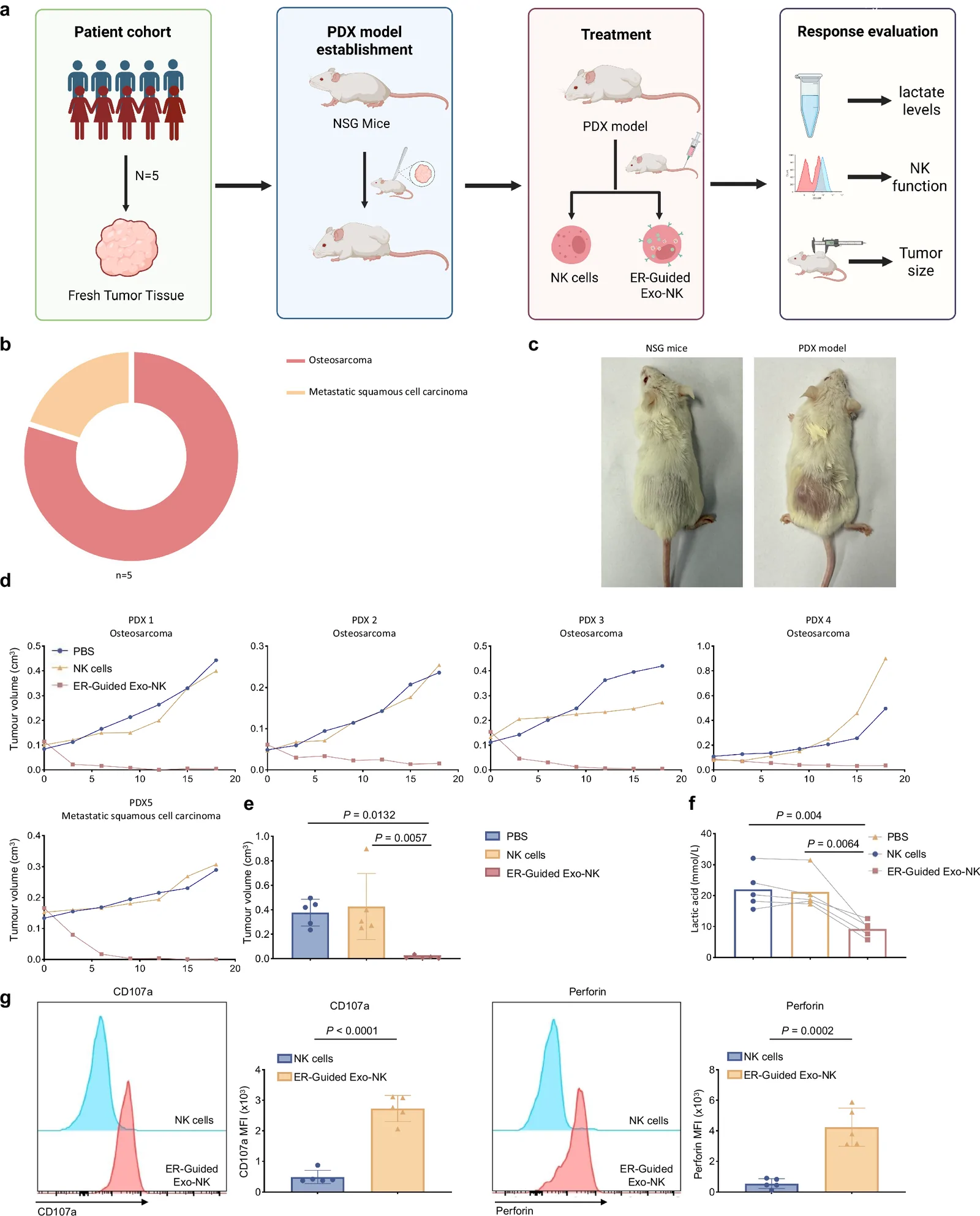
ER-guided Exo-NK suppresses patient-derived xenograft growth and lactate accumulation. **a** Patient-derived xenograft (PDX) establishment and treatment scheme. Fresh tumour specimens from five patients were implanted into NOD scid gamma mice and treated with PBS, NK cells or ER-Guided Exo-NK cells. Created in BioRender. Chai, X. (2026) https://BioRender.com/o8npq6k. **b** PDX cohort composition: four osteosarcoma cases and one metastatic squamous cell carcinoma case. **c** Representative mouse images before and after PDX establishment. **d** Tumour growth kinetics of individual PDX models; PDX1–PDX4, osteosarcoma; PDX5, metastatic squamous cell carcinoma. **e** Endpoint tumour volume; *n* = 5 PDX models per treatment group, mean ± SD; one-way ANOVA with two-sided Tukey’s multiple-comparisons test. Exact *P* values versus ER-Guided Exo-NK are *P* = 0.0132 for PBS and *P* = 0.0057 for NK cells. **f** Intratumoural lactate; *n* = 5 PDX models per treatment group, mean ± SD; one-way ANOVA with two-sided Tukey’s multiple-comparisons test. Exact *P* values versus ER-Guided Exo-NK are *P* = 0.004 for PBS and *P* = 0.0064 for NK cells. **g** Flow-cytometry histograms and quantification of CD107a and perforin in tumour-associated NK cells; *n* = 5 PDX models per group, mean ± SD; unpaired two-tailed *t*-test for each marker; *P* < 0.0001 for CD107a and *P* = 0.0002 for perforin. Source data are provided as a Source data file.

Clinically, we sought to strengthen the translational relevance of our two-compartment platform by validating its efficacy in patient-derived xenograft (PDX) models established from freshly resected tumour tissues (*n* = 5) implanted into NSG mice (Fig. 6a–c). This PDX cohort encompassed four osteosarcoma cases and one metastatic squamous cell carcinoma case, thereby capturing inter-patient heterogeneity and histological diversity (Fig. 6b). After successful engraftment, mice bearing established PDX tumours were treated with PBS, unmodified NK cells, or ER-Guided Exo-NK cells, and responses were evaluated using a multimodal readout comprising tumour growth kinetics, intratumoural lactate levels, and endpoint NK-cell functionality (Fig. 6a, d–g). Across individual PDXs, ER-Guided Exo-NK cells consistently restrained tumour outgrowth relative to both PBS and conventional NK-cell transfer (Fig. 6d). When responses were integrated across the five PDX models, ER-Guided Exo-NK treatment resulted in a significantly reduced endpoint tumour burden compared with PBS (*P* = 0.0132) and NK cells (*P* = 0.0057) (Fig. 6e). In parallel, metabolic profiling of tumours revealed that ER-Guided Exo-NK therapy markedly decreased intratumoural lactate levels versus PBS (*P* = 0.004) and NK-cell transfer (*P* = 0.0064) (Fig. 6f), supporting effective in vivo lactate depletion as a core pharmacodynamic consequence of the ER-guided exocytic delivery strategy. Importantly, this metabolic remodelling translated into a reinforced effector phenotype at the tumour site. At endpoint, NK cells recovered from tumours in the ER-Guided Exo-NK group exhibited significantly enhanced degranulation (CD107a; *P* < 0.0001) and elevated perforin expression (*P* = 0.0002) compared with NK cells in the conventional transfer group (Fig. 6g). Collectively, these PDX data demonstrate that the dual-compartment platform maintains efficacy across heterogeneous patient-derived tumours, coupling intratumoural lactate reduction with restoration of NK-cell cytotoxic capacity, and thereby providing a clinically relevant validation for the translatability of ER-Guided Exo-NK therapy.

### The dual-compartment platform revitalizes T cell- and macrophage-driven immunotherapies through lactate depletion

**Fig. 7:**
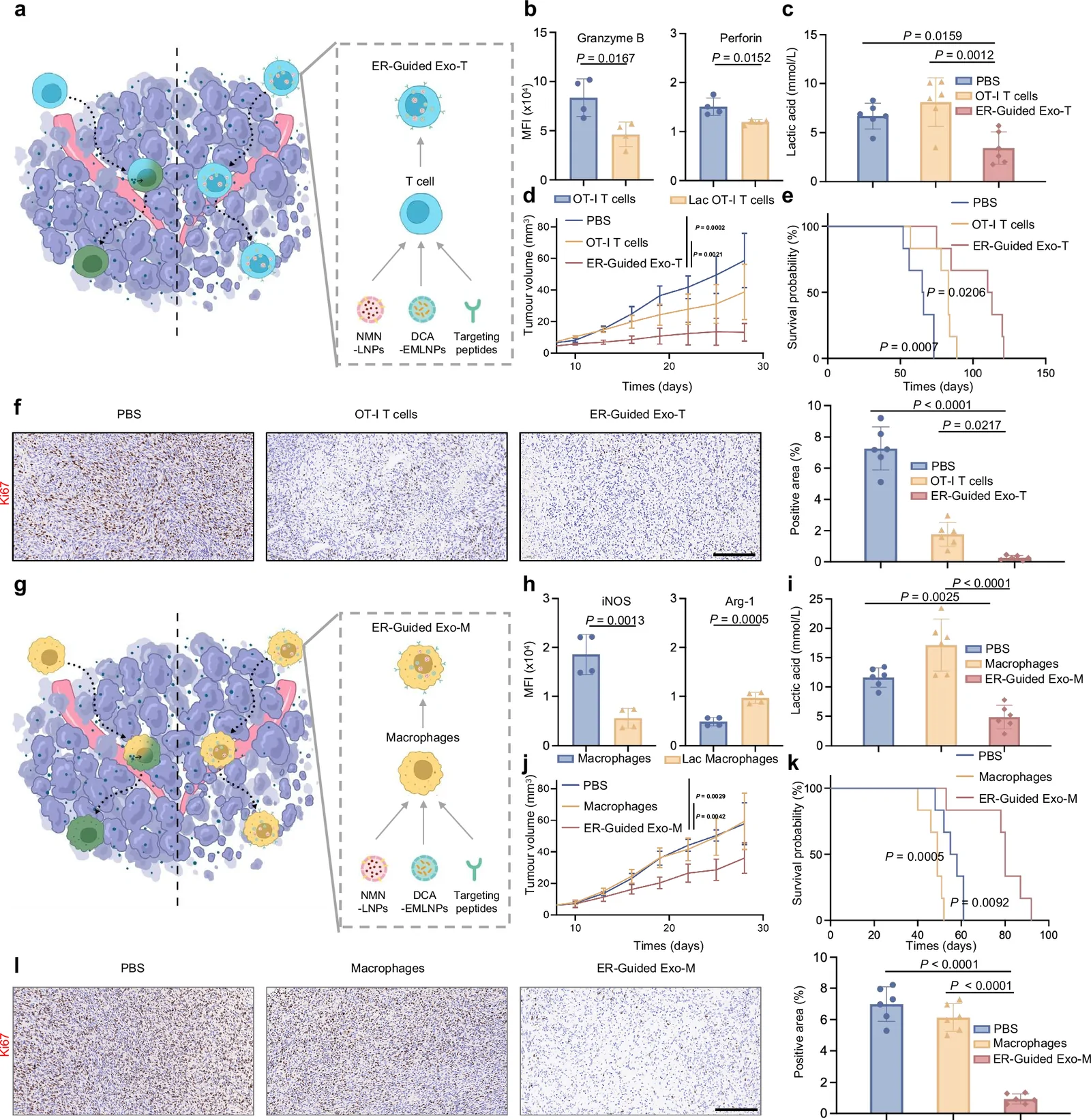
The dual-compartment platform extends to T-cell and macrophage therapies. **a** ER-guided Exo-T construction by co-loading primary CD8⁺ T cells with NMN-LNPs and DCA-EMLNPs, followed by MC38-homing peptide decoration. **b** Granzyme B and perforin in CD8⁺ T cells with or without 20 mM lactate; *n* = 4 independent OT-I CD8⁺ T-cell cultures per group, mean ± SD; unpaired two-tailed *t*-test for each marker. **c**–**f** MC38-OVA tumour responses after PBS, OT-I T cells or ER-Guided Exo-T treatment. **c** Intratumoural lactate; *n* = 6 mice per group, mean ± SD; one-way ANOVA, Tukey; exact *P* values are shown in the panel. **d** Tumour growth; *n* = 6 mice per group, mean ± SD; two-way repeated-measures ANOVA, Sidak. **e** Survival; *n* = 6 mice per group; log-rank test. **f** Ki-67 staining and quantification; *n* = 6 tumours from 6 mice per group, mean ± SD; one-way ANOVA, Tukey; *P* < 0.0001 for PBS and *P* = 0.0217 for OT-I T cells versus ER-Guided Exo-T; scale bar, 150 µm. **g** ER-Guided Exo-M preparation using bone-marrow-derived macrophages. **h** Inducible nitric oxide synthase and arginase-1 in macrophages with or without 20 mM lactate; *n* = 4 independent macrophage cultures per group, mean ± SD; unpaired two-tailed *t*-test for each marker. **i**–**l** MC38 tumour responses after PBS, macrophage or ER-Guided Exo-M treatment. **i** Intratumoural lactate; *n* = 6 mice per group, mean ± SD; one-way ANOVA, Tukey; *P* = 0.0025 for PBS and *P* < 0.0001 for macrophages versus ER-Guided Exo-M. **j** Tumour growth; *n* = 6 mice per group, mean ± SD; two-way repeated-measures ANOVA, Sidak. **k** Survival; *n* = 6 mice per group; log-rank test. **l** Ki-67 staining and quantification; *n* = 6 tumours from 6 mice per group, mean ± SD; one-way ANOVA, Tukey; both *P* < 0.0001 versus ER-Guided Exo-M; scale bar, 150 µm. Source data are provided as a Source data file.

To validate the universal applicability of our exocytosis-hijacking strategy beyond NK cells, we engineered dual-compartment platforms for T cells (ER-Guided Exo-T) and macrophages (ER-Guided Exo-M). We engineered an ER-Guided Exo-T variant by adapting the platform to primary CD8⁺ T cells through co-loading of NMN-LNPs (sustaining intracellular NAD⁺) and DCA-EMLNPs (depleting pericellular lactate), followed by tumour-targeting peptide conjugation (Fig. 7a). Lactate exposure suppressed T-cell cytotoxic programmes, reducing Granzyme B and Perforin (Fig. 7b; MFI histograms in Supplementary Fig. 8a, b). To further delineate the molecular and bioenergetic changes induced by NMN-LNP conditioning in CD8⁺ T cells, we performed Seahorse flux analysis and bulk RNA-seq. NMN-LNP-treated OT-I T cells displayed an increased OCR profile and significantly elevated maximal respiration, spare respiratory capacity, basal respiration and ATP-linked respiration (Supplementary Fig. 8e, f). In parallel, transcriptomic profiling revealed activation of cytotoxicity-associated programmes, including regulation of cell killing and leukocyte-mediated cytotoxicity pathways (Supplementary Fig. 8o, p), consistent with a strengthened effector-state In parallel, lactate lowered the NAD⁺/NADH ratio in OT-I CD8⁺ T cells, which was restored by NMN-LNPs and further increased by co-loading with DCA-EMLNPs; the NAD⁺/NADH ratio positively correlated with Granzyme B expression (Supplementary Fig. 9a–c). We next assessed whether this NMN-LNP-mediated redox recovery was associated with restoration of IFN-γ production in CD8^+^ T cells. Lactate strongly suppressed IFN-γ secretion and reduced the percentage of IFN-γ^+^ T cells, whereas NMN-LNP treatment restored both extracellular IFN-γ release and intracellular IFN-γ positivity under lactate stress (Supplementary Fig. 15a–c). Consistently, IFN-γ^+^ T-cell frequency showed a positive correlation with the intracellular NAD^+^/NADH ratio, further supporting a link between NAD^+^ restoration and cytokine-producing effector function in lactate-stressed T cells (Supplementary Fig. 15d). To confer tumour specificity, a phage-selected MC38-binding peptide was displayed on the T-cell surface. In an MC38-OVA model, mice randomised on day 7 received PBS, unmodified T cells or ER-Guided Exo-T. ER-Guided Exo-T lowered intratumoural lactate (Fig. 7c), slowed tumour growth (Fig. 7d) and extended survival relative to controls (Fig. 7e), with reduced Ki-67 staining at endpoint (Fig. 7f). To ensure that full dual-compartment loading does not blunt endogenous T-cell effector programmes, we assessed canonical cytolytic outputs and found no significant differences in granzyme B or perforin levels among untreated T cells, NMN-LNPs-T cells, DCA-EMLNPs-T cells and NMN-LNPs-DCA-EMLNPs-T cells under the activation conditions used (Supplementary Fig. 8i–l). Safety profiling showed no increase in cytokines associated with cytokine-release syndrome (TNF-α, IL-6, IFN-γ, IL-1β, IL-18; Supplementary Fig. 9d), no elevation in ALT, AST or CK (Supplementary Fig. 14b), and no histopathological evidence of injury in major organs (Supplementary Fig. 12a).

Similarly, we generated ER-Guided Exo-M by arming bone marrow-derived macrophages with identical payloads and targeting moieties (Fig. 7g). Lactate polarised macrophages toward an immunosuppressive state (iNOS↓, Arg-1↑; Fig. 7h; MFI histograms in Supplementary Fig. 8c, d). We next assessed whether NMN-LNP conditioning rewires macrophage bioenergetic fitness and transcriptional programmes. Seahorse profiling showed an increased OCR trajectory in NMN-LNP-treated macrophages, accompanied by significantly higher maximal respiration, spare respiratory capacity, basal respiration and ATP-linked respiration (Supplementary Fig. 8g, h). Bulk RNA-seq further indicated upregulation of activation-associated signaling pathways, including STAT/ERK-linked phosphorylation programmes and D-glucose transmembrane transport, together with macrophage differentiation-related signatures (Supplementary Fig. 8q, r). Consistently, lactate reduced the NAD⁺/NADH ratio in macrophages, which was rescued by NMN-LNPs and further enhanced upon co-loading with DCA-EMLNPs; the NAD⁺/NADH ratio positively correlated with iNOS expression (Supplementary Fig. 10a–c). We evaluated TNF-α as a macrophage-relevant antitumour cytokine. Lactate reduced TNF-α secretion from macrophages, whereas NMN-LNP treatment restored TNF-α production under lactate stress (Supplementary Fig. 15e). TNF-α levels also positively correlated with the intracellular NAD⁺/NADH ratio, indicating that NMN-LNP-mediated redox restoration supports macrophage-relevant antitumour cytokine output (Supplementary Fig. 15f). In vivo, ER-Guided Exo-M reduced intratumoural lactate (Fig. 7i), curtailed MC38 growth (Fig. 7j) and prolonged survival (Fig. 7k), with lower Ki-67 indices at endpoint (Fig. 7l). Likewise, to exclude a global disruption of macrophage secretory function by the complete dual-compartment formulation, we quantified cytokine outputs and observed comparable TNF-α and IL-6 levels among untreated macrophages, NMN-LNPs-macrophages, DCA-EMLNPs-macrophages and NMN-LNPs-DCA-EMLNPs-macrophages (Supplementary Fig. 8m, n), indicating preserved endogenous cytokine secretion. As with ER-Guided Exo-T, circulating cytokines, serum biochemistry and organ histology were unaltered (Supplementary Figs. 10d, 14c and 13a).

Collectively, the exocytosis-hijacking strategy demonstrates cross-lineage applicability: ER-Guided Exo-T and Exo-M restore effector functions through coordinated NAD⁺ maintenance and spatially focused lactate depletion, achieving potent tumour control without compromising safety. This establishes the ER-Golgi secretory pathway as a versatile biological conduit for metabolic reprogramming across adoptive cell therapies.

## Discussion

This study develops an exocytosis-directed dual-compartment lipid carrier platform to mitigate lactate-driven resistance to adoptive cell therapy in solid tumours. We identify lactate accumulation as a soluble suppressive feature of the osteosarcoma microenvironment and show that NMN-LNP-mediated NAD⁺ support, although able to restore NK-cell effector markers and cytotoxicity at early time points, does not sustain antitumour activity when extracellular lactate pressure persists. By combining intracellular NMN delivery with ER-hybrid DCA carriers that undergo ER-Golgi-associated exocytic export, the platform links immune-cell metabolic support with local suppression of tumour lactate production. This design reduces intratumoural lactate, restores NK-cell cytotoxic function, improves tumour control and extends survival in xenograft and patient-derived tumour models. More broadly, these findings reposition engineered immune cells from solely cytotoxic effectors to living vehicles for spatially gated metabolic intervention.

Our results align with the emerging view that tumour-derived lactate is not simply a glycolytic by-product, but an active regulator of immune escape. Lactic acid has been shown to blunt T-cell and NK-cell immunosurveillance^9^, polarize tumour-associated macrophages toward a tumour-promoting state^4^, support tumour-infiltrating regulatory T cells^30^ and reinforce exhausted T-cell dysfunction through lactate metabolism^10^. These studies provide an important framework for interpreting the lactate-rich state observed in our osteosarcoma models. Our data extend this concept to engineered NK-cell therapy by showing that metabolic support of the immune cell is insufficient if the extracellular suppressive metabolite remains in place. In this setting, lactate is both a tumour-derived environmental constraint and a barrier that limits the durability of intracellular NAD⁺ restoration. NMN-LNPs improved redox balance and temporarily restored NK-cell cytotoxicity, but this benefit diminished over time and failed to translate into durable tumour control. These findings suggest that intracellular metabolic rescue must be paired with relief of the suppressive extracellular metabolite burden to achieve sustained immune-cell function.

The component-dissection experiments further support the non-redundant roles of the two metabolic modules. DCA-containing ER-hybrid carriers primarily remodel the lactate-rich tumour niche, whereas NMN-LNPs reinforce intracellular NAD⁺ metabolism, mitochondrial fitness and effector competence. In our system, DCA alone reduces lactate but does not fully restore effector function, whereas NMN alone transiently supports cytotoxicity but does not provide durable tumour control. Their combination therefore addresses two coupled but distinct constraints: tumour-derived lactate overload and immune-cell metabolic insufficiency. The ER–Golgi trafficking route adds an essential spatial dimension to this intervention by using immune cells as local delivery vehicles rather than relying on systemic exposure to a small-molecule metabolic modulator. The imaging, trafficking-inhibition and LC–MS/MS mass-balance analyses support a secretory-route-dependent export mechanism, while the in vivo lactate reduction, immune-cell infiltration, effector restoration and tumour-control data link this trafficking behaviour to therapeutic activity. Thus, the platform does not simply encapsulate DCA and NMN; it coordinates where and when metabolic modulation occurs.

A central conceptual advance of this work is the co-option of the ER–Golgi secretory pathway as an outward delivery route for cellular therapy. Conventional nanoparticle delivery strategies have largely focused on cellular uptake, endosomal escape and intracellular cargo release. By contrast, our ER-hybrid nanocarriers are designed to enter therapeutic immune cells and then exploit endogenous secretory trafficking for outward cargo export at the tumour–immune-cell interface. This “internalize-and-release” logic is particularly suited for adoptive cell therapy, where engineered cells actively traffic to tumours, engage target cells and form local contact zones. In this setting, the therapeutic cell provides both biological targeting and a temporally regulated delivery compartment. Such a strategy may be especially useful for agents such as DCA, whose systemic administration is limited by dose-dependent toxicity, but whose local metabolic activity can be beneficial within lactate-rich tumours.

Several limitations should be considered. First, although the PDX experiments support translational relevance, the number of models remains limited and is composed mainly of osteosarcoma samples, with one metastatic squamous cell carcinoma model included to probe broader applicability. Second, the use of immunodeficient mice also limits assessment of how the platform interacts with a complete endogenous immune system. Extension to OT-I CD8⁺ T cells and macrophages suggests modular adaptability, but these cell types differ in activation requirements, effector mechanisms, targeting peptides and tumour models; these data should therefore be interpreted as proof of adaptability rather than equivalence across cell therapies. Future studies should evaluate this strategy in larger tumour panels, immunocompetent settings, repeated-dosing regimens and manufacturing workflows closer to clinical cell therapy production.

## Methods

### Ethics statement

All research involving human participants, human biospecimens and animals complied with all relevant ethical regulations. The collection and use of human tumour tissues and peripheral blood samples were approved by the Ethics Committee of The Second Affiliated Hospital, Zhejiang University School of Medicine (Approval (2024) Ethics Review (Research) No. (0735)). The collection and use of patient tumour tissues for patient-derived xenograft establishment were approved by the same institution (Approval (2021) Ethics Review (Research) No. (0977)). Written informed consent was obtained from all participants, or from their legal guardians, before sample collection. All human materials were handled as de-identified samples, and no identifiable private information was accessed by the investigators. The study was conducted in accordance with the Declaration of Helsinki. All animal procedures were approved by the Institutional Animal Care and Use Committee of Deruikang Biotechnology Co., Ltd. (Approval No. DRK20240601001) and were conducted in accordance with the approved institutional protocols.

### Cell lines

HOS human osteosarcoma cells were obtained from Procell Life Science & Technology (Wuhan, China; CL-0360). MC38 murine colon adenocarcinoma cells were obtained from the Cell Bank of the Chinese Academy of Sciences (Shanghai, China; SCSP-5431). HEK293T cells used for lentiviral packaging were obtained from the Cell Bank of the Chinese Academy of Sciences (Shanghai, China; SCSP-502). HOS and MC38 cells were cultured in Dulbecco’s Modified Eagle’s Medium (DMEM; Gibco), supplemented with 10% fetal bovine serum (FBS; Gibco) and 1% penicillin-streptomycin (P/S; Gibco). HEK293T cells were maintained under standard DMEM-based culture conditions and were used for lentiviral particle production. Cells were maintained at 37 °C in a humidified incubator with 5% CO₂ and were regularly tested to confirm the absence of mycoplasma contamination. MC38-OVA cells were generated from parental MC38 cells by lentiviral transduction and puromycin selection as described below. GFP-HOS cells were generated from parental HOS cells by lentiviral transduction with a GFP reporter vector, followed by antibiotic selection or fluorescence-based enrichment. Stable expression was confirmed before downstream experiments.

### Primary cells

#### Human NK cells

Peripheral blood mononuclear cells (PBMCs) were isolated from fresh human blood samples using density gradient centrifugation with Ficoll-Paque PLUS (GE Healthcare). NK cells were then purified from PBMCs via negative selection using the EasySep™ Human NK Cell Isolation Kit (STEMCELL Technologies), following the manufacturer’s instructions. The isolated NK cells (CD3⁻CD56⁺) were cultured in RPMI 1640 medium supplemented with 10% fetal bovine serum (FBS; Gibco), 1% penicillin-streptomycin (P/S; Gibco), and 100 IU/mL interleukin-2 (IL-2; PeproTech) at 37 °C in a humidified incubator with 5% CO₂. NK-cell purity was verified by flow cytometry as live CD3⁻CD56⁺ cells before downstream experiments. Unless otherwise stated, functional assays were performed using IL-2-maintained NK cells that were subsequently stimulated by tumour-cell co-culture or lactate-stress conditions, rather than unstimulated resting NK cells.

#### Mouse CD8⁺ T cells

Spleens were aseptically harvested from OT-I transgenic mice (C57BL/6 background), which express a T-cell receptor specific for the OVA₂₅₇-₂₆₄ peptide. Single-cell suspensions were prepared by mechanical dissociation through a 70-μm cell strainer in RPMI 1640 medium. Red blood cells were lysed using Red Blood Cell Lysis Buffer (Invitrogen) for 2–3 min at room temperature, followed by washing with phosphate-buffered saline (PBS; Gibco). CD8⁺ T cells were isolated using CD8a MicroBeads (Miltenyi Biotec) according to the manufacturer’s protocol. Cells were passed through MS columns placed in a magnetic separator, and the labeled CD8⁺ T cells were eluted as the positive fraction. Purity of isolated CD8⁺ T cells was confirmed by flow cytometry, typically exceeding 90%. Isolated CD8⁺ T cells were cultured in RPMI 1640 medium supplemented with 10% FBS, 1% P/S, 1 mM sodium pyruvate, nonessential amino acids, 20 mM HEPES, and 50 U/mL IL-2 (PeproTech). For activation, cells were stimulated with 1 μg/mL OVA₂₅₇-₂₆₄ peptide (InvivoGen).

### Mouse bone marrow-derived macrophages (BMDMs)

Bone marrow cells were harvested from the femurs and tibias of C57BL/6 mice by flushing with sterile PBS using a 26 G needle. Cells were cultured in non-tissue culture-treated Petri dishes in DMEM/F12 medium supplemented with 10% FBS, 1% P/S, and 20% L929 cell-conditioned medium as a source of macrophage colony-stimulating factor (M-CSF). Cultures were maintained at 37 °C in a humidified atmosphere with 5% CO₂. On day 3, an equal volume of fresh medium was added. On day 7, adherent cells were washed with warm PBS and gently detached using a non-enzymatic cell dissociation solution (Cellstripper; Corning). The resulting BMDMs were resuspended in DMEM/F12 medium and used for subsequent experiments.

### NK-, T-cell and macrophage preconditioning and effector-function assays

Primary human NK cells were maintained in IL-2-containing complete medium before functional assays. In direct NK–HOS co-culture and cytotoxicity assays, HOS tumour cells served as the target-cell stimulus, and no additional activation was applied. For NK cells treated without tumour-cell co-culture, including control, lactate-, NMN-, NMN-LNP-, DCA-EMLNP-, or combined nanoparticle-treated groups, cells were uniformly restimulated with HOS cells for 4 h before effector-function analysis. This standardized 4-h HOS restimulation was used to evaluate the ability of preconditioned NK cells to respond to tumour-cell challenge. After stimulation, NK-cell effector competence was assessed by CD107a degranulation and intracellular perforin, granzyme B and IFN-γ expression. For IFN-γ secretion assays, culture supernatants were collected after tumour-cell stimulation and analysed by ELISA. For OT-I CD8⁺ T-cell assays, MC38-OVA tumour cells served as the antigen-specific target-cell stimulus. In direct OT-I T-cell–MC38-OVA co-culture experiments, no additional activation was applied. For OT-I T cells treated without tumour-cell co-culture, including lactate-, NMN-, NMN-LNP-, DCA-EMLNP- or combined nanoparticle-treated groups, cells were uniformly restimulated with MC38-OVA cells for 4 h before effector-function analysis. This standardized antigen-specific restimulation was used to evaluate the ability of preconditioned OT-I T cells to respond to OVA-expressing tumour-cell challenge. T-cell effector competence was assessed by granzyme B, perforin and IFN-γ readouts. For macrophage assays, MC38 tumour cells served as the tumour-cell stimulus. In direct macrophage–MC38 co-culture experiments, no additional activation was applied. For macrophages treated without tumour-cell co-culture, including lactate-, NMN-, NMN-LNP-, DCA-EMLNP- or combined nanoparticle-treated groups, cells were uniformly restimulated with MC38 cells for 4 h before functional analysis. This standardized MC38 restimulation was used to evaluate the ability of preconditioned macrophages to respond to tumour-cell challenge. Macrophage functional status was assessed by iNOS, Arg-1 and TNF-α readouts.

### Plasmids and lentiviral transduction

To generate MC38 cells stably overexpressing ovalbumin (OVA), the plasmid PGMLV-CMV-MCS-EF1-Puro-WPRE (Genomeditech) was used for lentiviral vector construction. The full-length coding sequence of the OVA gene was amplified using the following primers:

- OVA Full-Length Amplification Primers: Forward: 5′-GCCACCATGGGCTCCATCG-3′ Reverse: 5′-AGGGGAAACACATCTGCCAAAG-3′

Lentiviral particles were produced by co-transfecting the OVA-expressing plasmid and packaging plasmids into HEK293T cells using Lipofectamine 3000 (Thermo Fisher Scientific), following the manufacturer’s instructions. Viral supernatants were collected 48–72 h post-transfection, filtered through a 0.45 μm membrane, and immediately used for transduction.

Lentiviral Transduction: MC38 cells were seeded into six-well plates at a density of 2 × 10⁵ cells per well and incubated for 24 h. Cells were then transduced with filtered lentiviral supernatants in the presence of 8 μg/mL polybrene. After 12–16 h, the viral medium was replaced with fresh complete DMEM. Cells were allowed to recover for 48 h, followed by puromycin selection (2 μg/mL), with the optimal dose determined via prior titration. Stable MC38-OVA cells were expanded and maintained in DMEM supplemented with 10% fetal bovine serum (FBS; Gibco) and 1% penicillin-streptomycin at 37 °C in a 5% CO₂ humidified incubator.

### Generation of GFP-expressing HOS cells

GFP-expressing HOS cells were generated by lentiviral transduction with a GFP reporter vector, followed by antibiotic selection or fluorescence-based enrichment. Stable GFP expression was confirmed by fluorescence microscopy before cytotoxicity and tumour-cell residual fluorescence assays. GFP-HOS cells were maintained under the same culture conditions as parental HOS cells.

### RNA extraction and library construction

Total RNA was extracted using TRIzol reagent (Invitrogen) following the manufacturer’s instructions. RNA quantity and purity were assessed using a NanoDrop 2000 spectrophotometer (Thermo Fisher Scientific), and RNA integrity was evaluated via electrophoresis on a 1% denaturing agarose gel and confirmed with an Agilent 2100 Bioanalyzer (Agilent Technologies). Only samples with RNA Integrity Number (RIN) > 7.0 were used for library preparation. From each sample, 1 μg of total RNA was used to isolate poly(A)+ mRNA using Dynabeads Oligo(dT)25 (Invitrogen) with two rounds of purification. The purified mRNA was fragmented into ~200 nt fragments using Magnesium RNA Fragmentation Module (NEB) at 94 °C for 5–7 min. First-strand cDNA was synthesized using SuperScript™ II Reverse Transcriptase (Invitrogen), followed by second-strand synthesis with DNA Polymerase I, RNase H (NEB), and dUTP (Thermo Scientific). After end-repair, A-tailing, and adapter ligation, the products were PCR-amplified (8 cycles) under the following conditions: 95 °C for 3 min; 8 cycles of 98 °C for 15 s, 60 °C for 15 s, 72 °C for 30 s; and final extension at 72 °C for 5 min. Final libraries with an average insert size of 300 ± 50 bp were selected and purified.

### Bioinformatics analysis of RNA-seq

Raw sequencing reads were subjected to quality control using fastp to remove adapters, low-quality bases, and ambiguous N nucleotides. For mouse OT-I CD8⁺ T cells, BMDMs, and MC38 and MC38-OVA tumour samples, reads were aligned to the mouse reference genome GRCm38/mm10 (https://ccb.jhu.edu/software/hisat2). Aligned reads were assembled into transcripts using StringTie, and transcript abundance was quantified as FPKM (Fragments Per Kilobase of exon model per Million mapped fragments). Differential expression analysis was performed using the edgeR package (https://bioconductor.org/packages/release/bioc/html/edgeR.html). Genes with a |log₂(fold change)| > 1 and *p*-value < 0.05 were defined as differentially expressed genes (DEGs). Gene Ontology (GO) and pathway enrichment analyses were conducted using DAVID and GSEA tools.

### LC–MS-based untargeted metabolomics

For LC–MS-based untargeted metabolomics, culture supernatants from primary human NK cells cultured alone and NK cells co-cultured with HOS osteosarcoma cells for 24 h under identical medium conditions were analysed. The analysis included two experimental groups, with *n* = 3 independent culture supernatant samples per group and six biological samples in total. A pooled quality-control (QC) sample was prepared by mixing equal aliquots from all analysed samples and was used to equilibrate the LC–MS system and evaluate analytical stability throughout the run. For metabolite extraction, each supernatant sample was thawed at 4 °C. Four millilitres of each sample were lyophilized, followed by addition of 1.5 mL pre-cooled 80% methanol in water. Samples were vortexed, sonicated on ice for 20 min, incubated at −20 °C for 1 h and centrifuged at 7000 × *g* for 20 min at 4 °C. The supernatants were collected and dried using a high-speed vacuum concentrator. Before LC–MS analysis, dried extracts were reconstituted in 150 µL pre-cooled 50% methanol in water and centrifuged at 20,000 × g for 15 min at 4 °C. The resulting supernatants were used for LC–MS injection. Untargeted metabolomics was performed using ultra-performance liquid chromatography coupled to quadrupole time-of-flight mass spectrometry. Chromatographic separation was performed on a Waters UPLC I-class system using a Waters ACQUITY UPLC HSS T3 column (2.1 × 100 mm, 1.7 µm). Samples were maintained at 4 °C in the autosampler. The injection volume was 5 µL, the column temperature was 40 °C, and the flow rate was 300 µL min⁻¹. Mobile phase A was 0.1% formic acid in water, and mobile phase B was acetonitrile containing 0.1% formic acid. The LC gradient was as follows: 0–2 min, 0% B; 2–6 min, 0–48% B; 6–10 min, 48–100% B; 10–12 min, 100% B; 12–12.1 min, 100–0% B; and 12.1–15 min, 0% B. Mass spectrometry was performed on an AB Sciex TripleTOF 5600+ mass spectrometer in both positive and negative electrospray ionization modes using information-dependent acquisition. The TOF MS scan range was *m*/*z* 60–1200, and the product-ion scan range was *m*/*z* 25–1200. The accumulation time was 0.25 s per spectrum for TOF MS scans and 0.05 s per spectrum for product-ion scans. In each acquisition cycle, eight precursor ions were selected for fragmentation with a collision energy of 30 V and a collision-energy spread of ±15 V. For positive-ion mode, the source temperature was 550 °C, ion source gas 1 was 40, ion source gas 2 was 50, curtain gas was 35, and ion spray voltage floating was 5500 V. For negative-ion mode, the source temperature was 550 °C, ion source gas 1 was 40, ion source gas 2 was 50, curtain gas was 35, and ion spray voltage floating was −4500 V. Raw data were processed using MS-DIAL ver. 4.9 for peak alignment, retention-time correction, and peak-area extraction. Metabolite annotation was performed by matching accurate mass with a mass tolerance of <10 ppm and MS/MS spectra with a mass tolerance of <0.01 Da against HMDB, MassBank, GNPS, and the in-house Bioprofile metabolite standard library. Ion peaks with >50% missing values within a group were removed before downstream statistical analysis. Peak areas from positive- and negative-ion modes were normalized separately by total peak area, and positive- and negative-ion features were then integrated for downstream analysis. Data were scaled using unit variance scaling before multivariate statistical analysis. QC performance was evaluated using total ion chromatogram overlap and principal-component analysis of QC and study samples. Differential metabolites were identified using variable importance in projection (VIP) > 1 and *P* < 0.05. Lactate abundance was compared between NK-alone and NK–HOS co-culture supernatants using an unpaired two-tailed Student’s *t*-test.

### Mice

Mus musculus was used for all animal experiments. Male C57BL/6J mice and male NOD scid gamma mice aged 6–8 weeks were used in this study. C57BL/6J mice (strain no. N000013) were purchased from GemPharmatech (Nanjing, China), and NOD scid gamma mice (NOD.Cg-Prkdc scid Il2rg em1Smoc, strain no. NM-NSG-001) were obtained from Shanghai Model Organisms Center (Shanghai, China). Mice were housed under specific pathogen-free conditions at 22–24 °C and 55–60% humidity on a 12-h light/12-h dark cycle, with ad libitum access to autoclaved food and water. Exact animal numbers for individual experiments are provided in the corresponding figure legends and Source Data. All animal procedures were approved by the Institutional Animal Care and Use Committee of Deruikang Biotechnology Co., Ltd. (Approval No. DRK20240601001) and were conducted in accordance with the approved institutional protocols. All surgical interventions were performed under anaesthesia via intraperitoneal injection of 1% pentobarbital sodium, and all efforts were made to minimize animal suffering. Animals were euthanized by an overdose of pentobarbital sodium, followed by confirmation of death. Sex was considered during study design. Only male mice were used to control experimental variables and reduce biological variability in tumour implantation, adoptive-cell-transfer and xenograft efficacy experiments. The study was not designed or powered to assess sex-specific differences, and no sex-specific conclusions are drawn. Because all animals used in the study were male, sex-disaggregated analysis was not performed. For all subcutaneous xenograft and patient-derived xenograft experiments, the maximal tumour burden permitted by the approved IACUC protocol was 2000 mm³. Tumour size was monitored regularly with calipers, and mice were euthanized at the experimental endpoint or earlier if humane endpoints were reached. The maximal permitted tumour size/burden was not exceeded in this study.

### Quantitative real-time PCR

Total RNA was extracted from human immune cells using TRIzol reagent (Invitrogen) according to the manufacturer’s instructions. The quantity and purity of RNA were measured using a NanoDrop 2000 spectrophotometer (Thermo Fisher Scientific). Complementary DNA (cDNA) was synthesized from 1 μg of total RNA using the PrimeScript™ RT Reagent Kit (Takara) following the manufacturer’s protocol. Quantitative real-time PCR (qRT-PCR) was performed with TB Green® Premix Ex Taq™ II (Takara) on a QuantStudio™ 6 Flex Real-Time PCR System (Applied Biosystems). Thermal cycling conditions were: initial denaturation at 95 °C for 30 s, followed by 40 cycles of 95 °C for 5 s and 60 °C for 34 s. Each reaction was run in triplicate. Gene expression levels were normalized to human GAPDH, and relative expression was calculated using the 2^−ΔΔCt^ method. The human genes analyzed in this study included GZMB, TNFA, IFNG, LAMP1, PRF1, PDCD1, HAVCR2 (TIM-3), TIGIT, and CTLA4. The primer sequences for these genes are listed in Table 1.

**Table 1:** Human primer sequences used for quantitative real-time PCR

Genes	Primer sequence
Homo-GZMB-F	TCAAAGAACAGGAGCCGACC
Homo-GZMB-R	TGGCGTAAGTCAGATTCGCA
Homo-TNF-F	TCTTCTCGAACCCCGAGTGA
Homo-TNF-R	ATGAGGTACAGGCCCTCTGA
Homo-IFNG-F	CAGCTCTGCATCGTTTTGGG
Homo-IFNG-R	CTGTTTTAGCTGCTGGCGAC
Homo-LAMP1-F	CCCCAGTCTCGTGATTGCTT
Homo-LAMP1-R	TGTTCACAGCGTGTCTCTCC
Homo-PRF1-F	ACCTTCATCCAAGCATGGGG
Homo-PRF1-R	TATTGTCCCACACGGTGCTC
Homo-PDCD1-F	CACGAGGGACAATAGGAGCC
Homo-PDCD1-R	GCCCATTCCGCTAGGAAAGA
Homo-TIGIT-F	ATGGGACGTACACTGGGAGA
Homo-TIGIT-R	GCAGTTACCCAGGCTTCTGT
Homo-HAVCR2-F	GGAATACAGAGCGGAGGTCG
Homo-HAVCR2-R	AGGGACACATCTCCTTTGCG
Homo-CTLA4-F	CTCAGCTGAACCTGGCTACC
Homo-CTLA4-R	CGCACAGACTTCAGTCACCT

### Lactate quantification

Lactate levels in serum, tumour tissues, and healthy tissues were quantified using a commercially available Lactate Assay Kit (Sigma-Aldrich), following the manufacturer’s protocol. For serum samples, whole blood was collected from anesthetized mice via retro-orbital puncture or cardiac puncture, allowed to clot at room temperature for 30 min, and centrifuged at 1500 × *g* for 10 min at 4 °C. The resulting supernatant was collected and stored at −80 °C until analysis. For tumour and healthy tissue samples, freshly excised tissues were weighed and homogenized in ice-cold lactate assay buffer (provided in the kit) using a bead mill homogenizer. Homogenates were centrifuged at 13,000 × *g* for 15 min at 4 °C, and supernatants were collected for immediate assay or stored at −80 °C. Samples were incubated with the lactate reaction mix at 37 °C for 30 min in a 96-well plate, and absorbance was measured at 450 nm using a microplate reader (Molecular Devices). Lactate concentrations were calculated by comparing absorbance values to a standard curve generated using known lactate concentrations. For serum, culture supernatants and tissue homogenate supernatants, lactate concentrations were expressed as mmol/L unless otherwise indicated. All samples were measured in technical duplicates or triplicates. Standard curves and negative controls were included in each assay plate to ensure assay accuracy and reproducibility.

### Flow cytometry

For in vitro analysis, cultured NK cells or tumour cells were collected, washed twice with PBS (GIBCO), and stained with Zombie Fixable Viability Dye (BioLegend) for 20 min at room temperature in the dark to assess viability. After washing, surface marker staining was performed by incubating cells with fluorochrome-conjugated antibodies for 30 min at 4 °C in FACS buffer (PBS with 2% FBS). For intracellular protein detection (e.g., Granzyme B, Perforin, IFN-γ), cells were fixed and permeabilized using the Intracellular Fixation & Permeabilization Kit (eBioscience), followed by antibody staining for 30 min at 4 °C in the dark. For in vivo tumour-infiltrating immune cell analysis, tumours were excised from treated mice, minced into small fragments, and digested in RPMI 1640 medium (GIBCO) containing 2 mg/mL collagenase IV, 0.1 mg/mL hyaluronidase, and 0.1 mg/mL DNase I (all from Sigma) at 37 °C for 30 min. The digested tissue was filtered through a 100-μm cell strainer, centrifuged at 300 × *g* for 5 min, and resuspended in PBS. Single-cell suspensions were subjected to viability staining with Zombie Aqua™, followed by surface and intracellular staining as described above. All samples were acquired using a Beckman Coulter CytoFLEX LX flow cytometer, and data were analyzed using FlowJo v10 software. Fluorescence compensation was performed using single-stain controls, and gating strategies were validated using fluorescence-minus-one (FMO) controls.

### Antibodies and antibody validation

All antibodies used in this study are listed below with supplier, catalogue number and clone information. Flow-cytometry antibodies were used at the manufacturer-recommended amount or dilution for the indicated species and application, unless otherwise stated. The following antibodies were used for flow cytometry: Brilliant Violet 510 anti-mouse CD45 (BioLegend, Cat. 103137, clone 30-F11), APC anti-mouse NK-1.1 (BioLegend, Cat. 108709, clone PK136), FITC anti-mouse CD107a/LAMP-1 (BioLegend, Cat. 121605, clone 1D4B), APC/Fire 750 anti-mouse Perforin (BioLegend, Cat. 154317, clone S16009A), PerCP anti-mouse/human CD11b (BioLegend, Cat. 101229, clone M1/70), FITC anti-mouse F4/80 (BioLegend, Cat. 123107, clone BM8), PE/Cyanine7 anti-Nos2/iNOS (BioLegend, Cat. 696813, clone W16030C), PE anti-mouse Arginase 1 (BioLegend, Cat. 165803, clone W21047I), APC anti-mouse CD8a (BioLegend, Cat. 162305, clone S18018E), Brilliant Violet 421 anti-human CD3 (BioLegend, Cat. 317343, clone OKT3), PE anti-human CD56 (BioLegend, Cat. 318306, clone HCD56), Brilliant Violet 510 anti-human CD45 (BioLegend, Cat. 368525, clone 2D1), FITC anti-human CD107a (BioLegend, Cat. 328605, clone H4A3), Alexa Fluor 647 anti-human/mouse Granzyme B (BioLegend, Cat. 515405, clone GB11), APC/Cyanine7 anti-human Perforin (BioLegend, Cat. 308127, clone dG9), Brilliant Violet 711 anti-human IFN-γ (BioLegend, Cat. 502539, clone 4S.B3) and Brilliant Violet 711 anti-mouse IFN-γ (BioLegend, Cat. 505835, clone XMG1.2). For intracellular flow-cytometric staining of IFN-γ, Brilliant Violet 711 anti-human IFN-γ and Brilliant Violet 711 anti-mouse IFN-γ were used at 5 µL per 10⁶ cells in 100 µL staining volume. For immunohistochemistry, the following primary antibodies were used: anti-human CD56 (Abcam, Cat. ab75813, clone EP2567Y, 1:200), anti-Ki-67 (Cell Signaling Technology, Cat. 9449, clone 8D5, 1:400), anti-IFN-γ (Abcam, Cat. ab231036, clone EPR21704, 1:500) and anti-phospho-PDHA1 (Abcam, Cat. ab177461, clone EPR12200, 1:250). For western blotting, the following primary antibodies were used: anti-phospho-PDHA1 (Abcam, Cat. ab177461, clone EPR12200, 1:1000), anti-total PDHA1 (Abcam, Cat. ab168379, clone EPR11098, 1:1000) and anti-Vinculin (Abcam, Cat. ab129002, clone EPR8185, 1:10000). Antibodies were selected based on the manufacturers’ validation information for the indicated species, applications and experimental conditions. For flow cytometry, antibody performance was assessed using single-stain compensation controls, fluorescence-minus-one controls and gating based on canonical marker combinations. For intracellular staining, fixation and permeabilization were performed according to the antibody-compatible staining protocol. For immunohistochemistry, staining specificity was supported by the expected tissue and cellular staining patterns and by no-primary-antibody negative controls. For western blotting, antibody specificity was evaluated based on the expected molecular weights of phospho-PDHA1, total PDHA1, and Vinculin, together with appropriate loading controls.

### Survival analysis from public datasets

To investigate the prognostic value of LDHA expression in sarcoma, survival analyses were conducted using clinical transcriptomic data from the TCGA-SARC (Sarcoma) and TARGET-OS (Osteosarcoma) datasets. Gene expression profiles and clinical follow-up data were retrieved and analyzed through the Bioinfo-ZS platform (https://www.bioinfo-zs.com/).

Patients were stratified into LDHA high-expression and low-expression groups based on the median transcript level of LDHA in each cohort. Kaplan–Meier survival curves were generated, and statistical significance was assessed using the log-rank (Mantel-Cox) test. A *p*-value < 0.05 was considered statistically significant. The survival plots were exported directly from the Bioinfo-ZS web interface. These analyses helped evaluate the correlation between LDHA expression and overall survival in both sarcoma and osteosarcoma patients.

### Preparation of NMN-loaded liposomes by microfluidic mixing

Liposomes were fabricated with a microfluidic LNP synthesizer (FluidicLab NP-S2, FluidicLab, China) equipped with a B1 staggered-herringbone micromixer. Hydrogenated soy phosphatidylcholine (HSPC; Avanti), cholesterol (Sigma–Aldrich), and mPEG-DSC (MW 2000; NOF) were dissolved in anhydrous ethanol at a molar ratio of 55: 40: 5 to give a 200 mM stock. After dissolution at 60 °C, the lipid solution was diluted to 15 mM (optimized to minimize particle size). A 300 mM ammonium sulfate buffer was prepared by dissolving 1.9812 g (NH₄)₂SO₄ (Sigma–Aldrich) in 50 mL ultrapure water. Both the lipid solution and the buffer were sterilized through 0.22 µm PTFE and MCE filters (Millipore), respectively. During microfluidic synthesis, the ethanolic lipid phase and the ammonium-sulfate phase were introduced at 4 mL min⁻¹ and 16 mL min⁻¹ (flow-rate ratio = 1: 4; total flow 20 mL min⁻¹). The freshly collected liposomal suspension was immediately incubated with 200 µM nicotinamide mononucleotide (NMN; MedChemExpress) dissolved in 0.9 % NaCl, allowing post-synthesis NMN loading under the indicated ionic-gradient-assisted incubation condition. Un-encapsulated NMN was removed by dialysis (Slide-A-Lyzer™ 20 kDa, Thermo Scientific) against 50 volumes of 0.9 % NaCl for 6 h at room temperature with one buffer exchange at 3 h.

### Isolation of endoplasmic-reticulum membranes and fabrication of ER-hybrid liposomes (EMLNPs)

ER membranes were harvested from freshly isolated primary human NK cells using an ER Isolation Kit (Sigma–Aldrich, Cat. #ER0100) according to the manufacturer’s instructions. In brief, 1 × 10⁸ NK cells were lysed under hypotonic conditions, and differential centrifugation was performed to remove nuclei, mitochondria, and debris. The resulting ER-enriched pellet was washed once in isolation buffer and resuspended in PBS. Total membrane protein was quantified with a BCA assay (Thermo Fisher, Cat. #23227). Across independent preparations, we typically recovered ~200–300 μg ER membrane protein from 1 × 10⁸ NK cells (BCA), corresponding to ~20–30 μg per 1 × 10⁷ NK cells. The input of 1 × 10⁸ NK cells per preparation was selected to match the recommended scale of the ER isolation kit and to ensure reproducible downstream fabrication. Conventional liposomes were generated exactly as described in Preparation of NMN-Loaded Liposomes (micro-fluidic mixing of HSPC: cholesterol: mPEG-DSC = 55: 40: 5, 15 mM total lipid; ethanol: buffer flow-rate ratio = 1: 4; total flow = 20 mL min⁻¹). Immediately after collection, the liposomal suspension was incubated with 10 mM dichloroacetate (DCA; Sigma-Aldrich) under the indicated ionic-gradient-assisted loading condition at 37 °C for 30 min. Un-encapsulated DCA was removed by dialysis (Slide-A-Lyzer™, 20 kDa MWCO, Thermo Scientific) against 0.9 % NaCl (50-fold volume, 6 h, single buffer exchange). Purified ER membranes were then mixed with the DCA-loaded liposomes at a protein: lipid mass ratio of 1: 2 and subjected to probe sonication (Branson Digital Sonifier; 20 % amplitude, 30 s on/30 s off, total 20 min, 4 °C) to induce membrane fusion. Based on the typical ER recovery above, one preparation (from 1 × 10⁸ NK cells) provides ~0.20–0.30 mg ER protein, sufficient to fuse with ~0.40–0.60 mg total lipid under the defined 1:2 (w/w) condition to generate a batch of EMLNPs for ex vivo cell armouring. The resulting ER-hybrid vesicles (EMLNPs) were isolated by ultracentrifugation (100,000 × *g*, 1 h, 4 °C), washed with PBS, and stored at 4 °C until further use. For ER-Guided Exo-T and ER-Guided Exo-M, ER membranes were isolated from the corresponding donor cell type, namely OT-I CD8⁺ T cells and bone marrow-derived macrophages, using the same ER-isolation and membrane-fusion workflow.

### Generation of ER-guided Exo-NK, ER-guided Exo-T and ER-guided Exo-M cells

To generate ER-Guided Exo-NK cells, purified human NK cells were washed and resuspended in serum-free or low-serum RPMI 1640 medium at 1 × 10⁸ cells mL⁻¹. For one-step dual nanoparticle loading, NMN-LNPs and DCA-EMLNPs were added simultaneously to the NK-cell suspension. NMN-LNPs were prepared using 200 μM NMN as the feed concentration, whereas DCA-EMLNPs were prepared using 10 mM DCA during active drug loading. DCA-EMLNPs were applied to cells at a nanoparticle-to-cell suspension volume ratio of 1:20, corresponding to an EMLNP suspension of approximately 100 μg lipid mL^−^¹. Cells were incubated for 2 h at 37 °C with gentle mixing, followed by three washes with PBS or complete medium to remove unbound nanoparticles. No intermediate washing step was performed between NMN-LNP and DCA-EMLNP exposure. For tumour-targeting decoration, nanoparticle-loaded NK cells were subsequently incubated with the HOS-targeting peptide under the peptide-conjugation conditions described above and washed to remove unbound peptide. The final product was designated ER-Guided Exo-NK. ER-Guided Exo-T and ER-Guided Exo-M cells were generated using the same one-step nanoparticle co-loading principle. Briefly, purified OT-I CD8⁺ T cells or bone marrow-derived macrophages were simultaneously incubated with NMN-LNPs and DCA-EMLNPs for 2 h at 37 °C, washed three times, and subsequently decorated with the corresponding MC38-homing peptide. Single-module controls were generated by incubating cells with NMN-LNPs alone or DCA-EMLNPs alone under otherwise identical conditions.

### Orthotopic tibial HOS osteosarcoma model

To establish an orthotopic osteosarcoma model, logarithmically growing HOS cells were washed and resuspended in sterile Matrigel solution. NSG mice were anaesthetized with isoflurane, and 1.5 × 10⁶ HOS cells in 10 μL Matrigel solution were injected into the right tibial medullary cavity through the proximal tibial plateau using a sterile Hamilton syringe. Sham-control mice received the same volume of vehicle alone. At the indicated endpoint, serum was collected and tumour-bearing tibiae, together with corresponding healthy control tissues, were harvested for lactate measurement. Tissues were homogenized in cold PBS or lactate assay buffer, centrifuged, and lactate concentrations were determined using a commercial lactate assay kit according to the manufacturer’s protocol.

### Verification of ER membrane hybridization via Coomassie Brilliant Blue staining

To confirm the successful hybridization of ER membranes with liposomes, Coomassie Brilliant Blue (CBB) staining was employed to detect ER-associated proteins integrated into the nanoparticle surface. Briefly, native LNPs and ER-hybridized EMLNPs were prepared at identical lipid concentrations (1 mg/mL) and centrifuged at 20,000 × *g* for 30 min to pellet the nanoparticles. The pellets were resuspended in PBS and mixed with SDS-PAGE loading buffer. Samples were subjected to SDS-PAGE on a 10% polyacrylamide gel, followed by staining with Coomassie Brilliant Blue R-250 solution (Bio-Rad) for 1 h and destaining with a methanol/acetic acid/water solution (50:10:40, v/v/v). The presence of distinct protein bands in EMLNPs—but not in unmodified LNPs—indicated successful incorporation of ER-derived membrane proteins.

### Co-culture of NK cells with tumour cells

To assess NK cell-mediated cytotoxicity, GFP-expressing HOS osteosarcoma cells were co-cultured with human NK cells in 24-well plates. HOS cells were seeded at a density of 5 × 10⁴ cells per well in 500 μL of complete DMEM and allowed to adhere overnight. The following day, NK cells were added at an effector-to-target (E:T) ratio of 1:1. Co-cultures were maintained at 37 °C in a 5% CO₂ incubator for 24 h. Tumour cell viability following co-culture was evaluated using two complementary approaches: Fluorescence Microscopy: Residual GFP-positive HOS cells were imaged using a Leica fluorescence microscope. Images were acquired from five randomly selected fields per well under fixed exposure settings. The average GFP fluorescence intensity—reflecting the remaining tumour burden—was quantified using ImageJ software and normalized to monoculture HOS controls.CCK-8 Assay: In parallel, tumour viability was also assessed using the Cell Counting Kit-8 (Dojindo). After removing NK cells via gentle PBS washes, 10 μL of CCK-8 reagent was added to each well and incubated for 1.5 h. Absorbance at 450 nm was measured using a microplate reader. Cell viability was calculated relative to untreated HOS control wells.

### Subcutaneous tumour implantation

For HOS osteosarcoma xenograft studies, NSG mice were maintained under specific pathogen-free conditions. HOS cells were harvested during logarithmic growth phase, washed twice in sterile PBS, and resuspended at 5 × 10⁶ cells per 100 μL PBS. Each mouse was injected subcutaneously into the right flank with 100 μL of the cell suspension. For colorectal tumour models, MC38 or MC38-OVA cells were implanted subcutaneously into C57BL/6J mice unless otherwise indicated. Cells were harvested during logarithmic growth phase, washed twice in sterile PBS, and injected into the right flank at 1 × 10⁶ cells in 100 μL PBS per mouse. Tumour growth was monitored every 2 days using a digital caliper, and tumour volume was calculated as 0.5 × length × width².

### Adoptive cell transfer (ACT)

Engineered immune cells (NK cells, CD8⁺ T cells, or bone marrow-derived macrophages) were freshly prepared, counted, and resuspended in sterile PBS. Prior to adoptive transfer, cells were engineered ex vivo according to the procedures described above in the “Methods”. After engineering, cells were washed to remove unbound materials and assessed for viability prior to infusion. Cells were administered via intravenous tail-vein injection under gentle warming to facilitate vasodilation. Unless otherwise indicated in the corresponding figure schematics, adoptive transfer was performed once weekly (q7d) during the indicated treatment window (e.g., starting on day 7 after tumour implantation). For each infusion, cells were delivered in a total volume of 200 μL sterile PBS at the following doses: 1 × 10⁷ NK cells per mouse, 5 × 10⁶ CD8⁺ T cells per mouse, or 5 × 10⁶ macrophages per mouse.

### Confocal imaging of ER/golgi and liposome co-localization in NK cells

ER and Golgi Probes: Purified NK cells were stained with ER-tracker™ Green (FITC channel; Thermo Fisher Scientific). Liposome Labeling: Liposomal formulations (LNPs or ER-hybrid EMLNPs) were fluorescently labeled with PKH26 Red Fluorescent Cell Linker Kit (Sigma-Aldrich). Labeled NK cells (1 × 10^6^) were mixed with PKH26-labeled liposomes at a lipid concentration of 0.2 mg/mL in complete RPMI 1640 medium. Co-incubation was performed at 37 °C for 2 h to allow initial uptake. Following this, cells were washed twice with PBS to remove free liposomes and then cultured in fresh medium. At 24, 48, and 72 h post-co-incubation, aliquots of NK cells were collected, washed with PBS, and fixed in 4% paraformaldehyde for 15 min at room temperature. Fixed cells were mounted onto glass slides using ProLong™ Gold Antifade Mountant with DAPI (Thermo Fisher Scientific). Confocal images were acquired on a Leica TCS SP8 confocal microscope equipped with 405 nm, 488 nm, and 561 nm laser lines. ER and Golgi signals were excited at 488 nm (FITC channel), and PKH26-labeled liposomes at 561 nm (TRITC channel). Z-stacks (0.5 μm steps) were collected using a 63× oil-immersion objective.

### Hematoxylin and Eosin (H&E) staining

Major organs—including heart, liver, spleen, lung, and kidney—were harvested from euthanized mice, fixed in 4% paraformaldehyde for 24 h, and embedded in paraffin. Tissue blocks were sectioned at 5-μm thickness using a rotary microtome (Leica RM2235). Sections were deparaffinized in xylene, rehydrated through graded alcohols, and stained with hematoxylin (Solarbio) for 5 min followed by eosin (Solarbio) for 2 min. After dehydration and mounting, slides were visualized using a bright-field microscope (Leica DM3000) to assess general tissue morphology and potential treatment-related toxicity.

### Immunohistochemistry (IHC)

Tumour tissues were collected and fixed in 4% paraformaldehyde, paraffin-embedded, and sectioned at 5 μm thickness. Sections were deparaffinized and rehydrated, followed by antigen retrieval using sodium citrate buffer (pH 6.0) at 95 °C for 15 min. Endogenous peroxidase activity was quenched with 3% hydrogen peroxide for 10 min. After blocking with 5% bovine serum albumin (BSA), sections were incubated overnight at 4 °C with primary antibodies against human CD56 (Abcam, ab75813, 1:200), Ki-67 (Cell Signaling Technology, #9449, 1:400), IFN-γ (Abcam, ab231036, 1:500), and phospho-PDHA1(Abcam, ab177461, 1:250). The next day, slides were washed and incubated with HRP-conjugated secondary antibodies for 30 min at room temperature. Signal was visualized using DAB substrate (Dako), and sections were counterstained with hematoxylin. Images were acquired using a Leica DM3000 microscope.

### Serum biochemistry and inflammatory marker analysis

To assess acute inflammatory responses after cell-based therapy, peripheral blood was collected from mice 24 h after the first intravenous infusion. Serum concentrations of TNF-α, IL-6, IFN-γ, IL-1β and IL-18 were measured using commercial ELISA kits according to the manufacturers’ instructions. To evaluate systemic organ toxicity after repeated treatment, terminal blood samples were collected at the experimental endpoint. Serum levels of alanine aminotransferase (ALT), aspartate aminotransferase (AST) and creatine kinase (CK) were measured using commercial colorimetric assay kits according to the manufacturers’ instructions. Major organs, including the heart, liver, spleen, lung and kidney, were harvested at the endpoint for H&E staining and histopathological assessment.

### In vitro tumour-targeting ability

To evaluate the tumour-targeting specificity of the engineered NK cells, ER-Guided Exo-NK (NMN-LNPs-DCA-EMLNPs-NK cells conjugated with tumour-targeting peptide) and non-targeted control NK cells (without peptide modification) were labeled using PKH26 Red Fluorescent Cell Linker Kit (Sigma-Aldrich) according to the manufacturer’s instructions. After labeling, cells were co-cultured with GFP-expressing HOS osteosarcoma cells (pre-seeded in 24-well plates at a density of 2 × 10⁵ cells/well) at a 1:1 ratio for 4 h in complete RPMI 1640 medium. The culture plates were imaged using a Leica DMi8 fluorescence microscope.

### In vivo tumour-targeting ability

To investigate the tumour-homing ability of ER-Guided Exo-NK in vivo, DiR/CY5.5-labeled NK cells were prepared using a DiR/CY5.5 dye (Thermo Fisher) at a final concentration of 5 μM for 15 min at 37 °C, followed by three PBS washes. Labeled ER-Guided Exo-NK cells (1 × 10^7^ cells in 200 μL PBS) or non-targeted NK cells were injected via tail vein into NSG mice bearing subcutaneous HOS tumours (established 7 days prior). Whole-animal fluorescence imaging was performed using an IVIS Spectrum imaging system (PerkinElmer) at 24, 48, 72, and 96 h post-injection to track biodistribution and tumour accumulation.

### Phage display screening for tumour-targeting peptides

Tumour-targeting peptides were screened using the Ph.D.-C7C random cyclic heptapeptide phage display library (New England Biolabs) using a cell-based biopanning strategy adapted from Lin et al.^31^, with full experimental details provided here. Screening was performed separately for HOS cells, MC38 cells, and patient-derived tumour cells from each PDX model to identify tumour-context-matched binding peptides. For each screening experiment, target tumour cells were seeded into culture dishes and grown to approximately 80% confluence before biopanning. Cells were washed with PBS and blocked with serum-containing medium to reduce nonspecific phage binding. The phage library was diluted in binding buffer and incubated with target tumour cells at 4 °C for 1 h with gentle rocking. Unbound phages were removed by repeated washing with PBS containing 0.1% Tween-20, and tumour-cell-bound phages were eluted using acidic glycine buffer and immediately neutralized with Tris-HCl buffer. The recovered phages were amplified in host bacteria according to the manufacturer’s instructions and used as the input pool for the next selection round. Three to four rounds of biopanning were performed for each tumour-cell type, with progressively increased washing stringency to enrich tumour-binding clones. After the final round, individual phage plaques were picked, amplified and sequenced to identify enriched peptide-encoding inserts. Candidate peptide sequences were ranked according to enrichment frequency and tumour-cell binding specificity. Binding of selected candidate peptides to the corresponding target tumour cells was validated by fluorescence-based cell-binding assays before use for immune-cell surface decoration. The HOS-binding peptide was used for ER-Guided Exo-NK targeting in HOS models, the MC38-binding peptide was used for ER-Guided Exo-T and ER-Guided Exo-M targeting in MC38-based models, and PDX-specific binding peptides were screened and validated using tumour cells derived from each corresponding PDX model before conjugation onto ER-Guided Exo-NK cells for PDX treatment experiments.

### Quantification of intracellular NAD^+^/NADH and NADP/NADPH

Intracellular NAD^+^/NADH ratios were quantified using an enzymatic cycling luminescence assay (NAD^+^/NADH-Glo^TM^ Assay, Promega, Cat. #G9071) following the manufacturer’s instructions. For NADP/NADPH, the NADP/NADPH-Glo^TM^ Assay (Promega, Cat. #G9081) was used. Briefly, activated human NK cells (or OT-I CD8⁺ T cells/bone-marrow-derived macrophages, as indicated) were cultured under lactate stress (20 mM sodium L-lactate) and treated with NMN-LNPs (±DCA-EMLNPs) for the indicated duration. Cells were then washed twice with pre-warmed PBS to remove extracellular materials and re-cultured in fresh medium. At each time point (0, 6, 12, 24, and 48 h), cells were harvested, counted, and lysed in assay lysis buffer. NAD(H) or NADP(H) signals were measured on a microplate reader (Synergy H1, BioTek) and converted to ratios according to the kit protocol. For correlation analyses, matched samples were analysed by flow cytometry for effector readouts (NK: CD107a MFI; T cells: Granzyme B MFI; macrophages: iNOS MFI) on the same day, and Pearson correlation coefficients were calculated.

### Pharmacological perturbation of ER-Golgi secretory trafficking

To interrogate the requirement of early secretory transport for EMLNP-enabled export, donor NK cells loaded with FITC-DG-EMLNPs were treated with inhibitors targeting ER→Golgi trafficking. Golgicide A (GCA; Sigma-Aldrich, Cat. #G0923) was used to inhibit GBF1-dependent ARF activation and Golgi trafficking; and FLI-06 (MedChemExpress, Cat. #HY-12443) was used to inhibit ER export. Inhibitors were applied at the indicated concentrations (GCA, 5–10 μM; FLI-06, 5–10 μM) with matched vehicle controls (DMSO). These concentrations are commonly used to inhibit early secretory trafficking and ER export in mammalian cells. After inhibitor treatment, FITC export was quantified using the dual-readout assay described above (supernatant fluorescence and intracellular FITC MFI).

### siRNA knockdown of COPI/COPII components in primary NK cells

For genetic perturbation of COPI/COPII trafficking, primary human NK cells were transfected with commercially available siRNAs targeting ARF1 or SEC23B. ARF1 was targeted using ARF1 siRNA (h) (Santa Cruz Biotechnology, sc-105086), and SEC23B was targeted using SEC23B siRNA (h) (Santa Cruz Biotechnology, sc-106539). A non-targeting control siRNA was used as the negative control. NK cells were transfected according to the manufacturer’s protocol and recovered in complete medium for 36–48 h before downstream assays. Subsequently, cells were loaded with FITC-DG-EMLNPs, washed, and FITC export was quantified using the dual-readout assay, including supernatant fluorescence and intracellular FITC MFI, at 24, 48, and 72 h.

### Seahorse XF mitochondrial stress test

Mitochondrial respiration was measured using the Seahorse XF Cell Mito Stress Test Kit (Agilent, Cat. #103015-100) on an Agilent Seahorse XF analyser (XF96 or XFe96). Cells (NK, OT-I CD8⁺ T cells, or macrophages) were plated on Seahorse cell culture microplates pre-coated as needed to ensure adherence. After treatment with NMN-LNPs (24 h) and washing, cells were equilibrated in Seahorse XF base medium (Agilent) supplemented with glucose, pyruvate, and glutamine, and incubated in a non-CO₂ incubator for 45–60 min before the assay. Oxygen consumption rate (OCR) was recorded at baseline and following sequential injections of oligomycin (1.5 μM), FCCP (1.0 μM), and rotenone and antimycin A (0.5 μM each), consistent with the manufacturer’s guidance. Basal respiration, ATP-linked respiration, maximal respiration and spare respiratory capacity were calculated using Seahorse Wave software.

### Patient-derived xenograft (PDX) establishment and treatment

Fresh patient-derived tumour tissues, including four osteosarcoma specimens and one metastatic squamous cell carcinoma specimen, were obtained with written informed consent under an institutional ethics-approved protocol from the Orthopedics Department of the Second Affiliated Hospital of Zhejiang University School of Medicine. Tumour fragments (~2–3 mm^3^) were implanted subcutaneously into NSG mice. When tumours reached the predefined volume range (50–150 mm^3^), mice were randomised into treatment groups and received engineered NK-cell therapies following the dosing schedule described under Adoptive Cell Transfer. Tumour volumes were measured using calipers at the indicated intervals. At endpoint, tumours were harvested for lactate quantification and flow-cytometric assessment of tumour-infiltrating NK-cell phenotypes and effector functions. Briefly, for each PDX model, tumour-binding peptides were individually screened against the corresponding patient-derived tumour cells and validated for binding before conjugation onto NK cells.

### LC–MS/MS quantification of DCA

Dichloroacetate (DCA) content was quantified by targeted ultra-performance liquid chromatography–tandem mass spectrometry (UPLC–MS/MS). A total of 24 samples were analysed, corresponding to four experimental groups with *n* = 6 independent samples per group. Samples included the fractions used to quantify DCA distribution, including intracellular DCA, extracellular DCA, passive leakage, and unrecovered DCA fractions. For sample preparation, each sample was thoroughly mixed, and 0.1 mL of sample solution was transferred into a 1.5 mL centrifuge tube, diluted with 0.9 mL ultrapure water, mixed, and subjected to ultrasonic extraction for 10 min. Samples were then centrifuged at 10,000 r.p.m. for 10 min, and the supernatants were filtered through a 0.22 µm membrane before injection. Chromatographic separation was performed on a Waters UPLC system coupled to a Waters XEVO-TQD triple-quadrupole mass spectrometer. Separation was performed using an AQ C18 column (2.1 × 100 mm, 1.7 µm). The column temperature was maintained at 30 °C, the autosampler temperature was 10 °C, the flow rate was 0.3 mL min⁻¹, and the injection volume was 10 µL. Mobile phase A was 0.1% formic acid in water, and mobile phase B was acetonitrile. DCA was detected in negative electrospray ionization mode using multiple-reaction monitoring. The main MS parameters were as follows: capillary voltage, 3 kV; cone voltage, 30 V; source temperature, 150 °C; desolvation temperature, 200 °C; desolvation gas flow, 800 L h^−^¹; and cone gas flow, 50 L h^−^¹. DCA standards were analysed under the same chromatographic and mass-spectrometric conditions to determine the retention time and to generate a calibration curve. Sample DCA content was calculated from peak-area data using the internal-standard calibration curve and the corresponding dilution factor. Extracellular and intracellular DCA fractions were calculated from culture supernatants and cell-associated extracts, respectively. Passive leakage was calculated from cell-free DCA-EMLNP samples incubated under identical conditions, and the unrecovered DCA fraction was calculated as 100% minus the extracellular and intracellular DCA fractions. Quantitative values were analysed using GraphPad Prism 9.0.0. Data are presented as mean ± SD, and statistical tests are described in the corresponding figure legends.

### Western blotting

Western blotting was performed to evaluate PDHA1 phosphorylation in tumour tissues. HOS tumour-bearing mice were treated with the indicated formulations, and tumour tissues were harvested at the defined experimental endpoint. Fresh tumour samples were washed with ice-cold PBS and homogenized in RIPA lysis buffer supplemented with protease and phosphatase inhibitor cocktails. Tissue lysates were incubated on ice and centrifuged at high speed at 4 °C to remove insoluble debris. Protein concentrations were determined using a BCA protein assay. Equal amounts of total protein were mixed with SDS loading buffer, denatured, separated by SDS–PAGE, and transferred onto PVDF membranes. After transfer, membranes were blocked with non-fat milk in TBST for 1 h at room temperature and incubated overnight at 4 °C with the primary antibody against phosphorylated PDHA1 (Abcam, ab177461, 1:1000). After washing with TBST, membranes were incubated with HRP-conjugated secondary antibody at room temperature, and p-PDHA1 bands were visualized using enhanced chemiluminescence. Because phosphorylated PDHA1 and total PDHA1 have the same expected molecular weight, total PDHA1 was detected on the same membrane after stripping and reprobing. Briefly, after p-PDHA1 imaging, the membrane was incubated with stripping buffer according to the manufacturer’s instructions to remove bound antibodies, washed thoroughly with TBST, re-blocked, and then incubated overnight at 4 °C with the primary antibody against total PDHA1 (Abcam, ab168379, 1:1000). After secondary antibody incubation, total PDHA1 bands were visualized under identical imaging conditions. Band intensities were quantified using ImageJ or equivalent densitometry software. PDHA1 phosphorylation was calculated as the ratio of p-PDHA1 to total PDHA1 from the same membrane, and Vinculin (Abcam, ab129002, 1:10000) was used to verify equal protein loading. Quantitative data were presented as mean ± SD, and statistical significance among groups was determined using one-way ANOVA with multiple-comparison correction.

### IFN-γ ELISA and intracellular flow-cytometric staining

IFN-γ secretion was quantified in culture supernatants using species-specific ELISA kits according to the manufacturer’s instructions. Human NK-cell supernatants were analysed using the Human IFN-γ ELISA MAX™ Deluxe Set (BioLegend, 430104), whereas mouse OT-I CD8⁺ T-cell supernatants were analysed using the Mouse IFN-γ ELISA MAX™ Deluxe Set (BioLegend, 430804). Briefly, supernatants from the indicated treatment groups were collected, clarified by centrifugation, and assayed in duplicate or triplicate. Absorbance was measured at 450 nm, and IFN-γ concentrations were calculated from recombinant IFN-γ standard curves.

For intracellular IFN-γ staining, cells were stimulated for 4–6 h in the presence of brefeldin A or monensin, harvested, washed, and stained with a fixable viability dye. Human NK cells were identified as live CD3⁻CD56⁺ cells, and mouse CD8⁺ T cells were identified as live CD3⁺CD8⁺ cells. Cells were then fixed, permeabilized and stained with Brilliant Violet 711 anti-human IFN-γ antibody [4S.B3] (BioLegend, 502539, 5 μL per 10⁶ cells in 100 μL staining volume) or Brilliant Violet 711 anti-mouse IFN-γ antibody [XMG1.2] (BioLegend, 505835, 5 μL per 10⁶ cells in 100 μL staining volume), respectively. Samples were acquired on a flow cytometer and analysed using FlowJo. IFN-γ production was reported as the percentage of IFN-γ⁺ cells within the indicated live immune-cell population.

### Software and code

No custom code or custom algorithms were generated for this study. Flow-cytometry data were analysed using FlowJo v10. Statistical analyses were performed using GraphPad Prism 9.0.0. Image quantification and densitometry were performed using ImageJ v1.53. RNA-seq data were processed using fastp v0.20.1, HISAT2 v2.1.0, StringTie v1.3.3b and edgeR v3.20.9. Gene Ontology and pathway enrichment analyses were performed using DAVID v6.8 and GSEA v4.3.3. Untargeted metabolomics data were processed using MS-DIAL ver. 4.9 and Python v3.8. Seahorse data were analysed using Seahorse Wave v2.6. Public survival analyses were performed using the Bioinfo-ZS web platform.

### Material availability

Newly generated materials described in this study, including MC38-OVA cells, GFP-HOS cells, tumour-binding peptide information and engineered immune-cell preparation protocols, are available from the corresponding author for academic research use, subject to institutional material-transfer agreements and applicable ethical, biosafety and regulatory requirements. Requests should be directed to the corresponding author listed in this paper.

### Statistics and reproducibility

Statistical analyses were performed using GraphPad Prism 9.0.0 unless otherwise stated. Data are presented as mean ± SD unless otherwise indicated. Exact sample sizes and experimental units are provided in the corresponding figure legends and Source Data. No statistical method was used to predetermine sample size. Sample sizes were chosen on the basis of feasibility, previous experience with similar in vitro and in vivo experiments, and commonly used sample sizes in comparable preclinical studies. No animals or biological samples were excluded from the analyses. For LC–MS-based untargeted metabolomics, ion features with >50% missing values within a group were removed as a prespecified feature-level data-processing step before downstream statistical analysis. Unless otherwise stated, endpoint measurements were obtained from distinct biological samples, including independent mice, independent cell cultures, independent patient-derived xenograft models, independently processed tumour samples, or independently prepared nanoparticle or cell-therapy batches. Longitudinal tumour-volume measurements were repeatedly obtained from the same mice over time and were analysed using two-way repeated-measures ANOVA followed by Sidak’s multiple-comparison test. For image-based quantification, multiple fields or cells from the same biological sample were averaged before statistical analysis where applicable, and the plotted experimental unit is specified in the corresponding figure legend. For in vivo therapeutic experiments, mice with established tumours were allocated to treatment groups after tumour establishment, with pretreatment tumour volumes balanced between groups where possible. For patient-derived xenograft experiments, mice were randomized into treatment groups when tumours reached the predefined volume range. The in vitro experiments were not randomized. The Investigators were not blinded to allocation during experiments and outcome assessment. Comparisons between two independent groups were performed using unpaired two-tailed Student’s *t*-test. Comparisons among multiple groups were performed using one-way ANOVA followed by Tukey’s multiple-comparison test for all pairwise comparisons or Dunnett’s multiple-comparison test when multiple treatment groups were compared with a single control group. Survival curves were estimated using the Kaplan–Meier method and compared using the two-sided log-rank Mantel–Cox test. Correlations were assessed using two-tailed Pearson correlation analysis. Normality was assessed using the Shapiro–Wilk test when sample size permitted, and equality of variance was assessed where appropriate. All hypothesis tests were two-sided unless otherwise stated. Exact *P* values are reported whenever possible, and *P* < 0.05 was considered statistically significant. Representative micrographs in Figs. 3b, c, i, j and 4b were reproduced in three independent experiments with similar results. For nanoparticle characterization, each independent experiment used an independently prepared nanoparticle batch. For cell-imaging experiments, each independent experiment used independently prepared NK-cell/nanoparticle samples processed and imaged on different experimental days.

### Reporting summary

Further information on research design is available in the Nature Portfolio Reporting Summary linked to this article.

## Supplementary information

Supplementary Information

Reporting Summary

Transparent Peer Review file

## Source data

Source Data

## Data Availability

The bulk RNA-seq data generated in this study have been deposited in the NCBI Sequence Read Archive under BioProject accession numbers PRJNA1391857, PRJNA1391541 and PRJNA1391664. PRJNA1391857 contains transcriptomes from tumour-associated NK cells and NK control cells, as well as tumour transcriptomes from in vivo treatment experiments. PRJNA1391541 contains transcriptomes from NK cells treated with NMN-LNPs. PRJNA1391664 contains transcriptomes from CD8⁺ T cells and macrophages treated with the corresponding NMN liposomal formulations. These RNA-seq datasets are publicly available under the accession codes listed above. The LC–MS-based untargeted metabolomics data generated in this study have been deposited in the BIG Sub OMIX database under accession number OMIX011385. These data are available under controlled access because they contain human biospecimen-associated omics data subject to participant consent, institutional ethics approval and China’s Human Genetic Resources regulations. The deposited files include raw mass spectrometry data, processed peak tables, sample metadata and metabolite annotation outputs used for downstream analysis. Access can be obtained by submitting a controlled-access application through the OMIX database, including a brief research proposal, the intended use of the data and evidence of ethics approval. Requests will be reviewed by the data access committee and the corresponding author to ensure consistency with the approved consent, ethical approval and applicable regulatory requirements. Data will be made available only for non-commercial academic research and may not be used for participant re-identification, redistribution or purposes outside the approved research plan. Access requests should be directed through the OMIX controlled-access portal and to the corresponding author. Requests are expected to receive an initial response within 20 working days. Once access is granted, the approved data will be available for the period specified in the approved OMIX controlled-access agreement. The targeted DCA LC–MS/MS data generated in this study have been deposited in Figshare and are publicly available at 10.6084/m9.figshare.32909318. The TCGA-SARC dataset used in this study is publicly available through The Cancer Genome Atlas via the Genomic Data Commons Data Portal. The TARGET-OS dataset used in this study is publicly available through the Therapeutically Applicable Research to Generate Effective Treatments programme via the Genomic Data Commons Data Portal. These third-party public datasets were used only for the survival analyses shown in Fig. 2h. Source data are provided with this paper. Source data underlying the quantitative panels in the main and Supplementary Figs. have also been deposited in Figshare and are publicly available at 10.6084/m9.figshare.32226321. The final Reporting Summary is provided with this paper. All relevant approvals and institutional authorizations related to China’s Human Genetic Resources regulations and the requirements of the Ministry of Science and Technology have been obtained for the generation, analysis, deposition and sharing of genetic information and materials relevant to this work. Data deposition and sharing were conducted within the approved scope of ethical approval, participant consent and institutional authorization. Source data are provided with this paper.
