# Supplementary material for: Dual compartment lipid carriers hijack exocytosis to empower natural killer cells against solid tumours
Source: Nat Commun. 2026 Jul 21;17:8906. doi: 10.1038/s41467-026-75852-6 (PMC13503815; doi:10.1038/s41467-026-75852-6)
Supplement: Supplementary file 1 [file 41467_2026_75852_MOESM1_ESM.pdf]

Supplementary Information of

Dual compartment lipid carriers hijack exocytosis to empower natural killer cells against solid tumours

Shixin Chen[1,2,3,4†], Zhenxuan Shao[1,2,3,4†], Yijia Zhou[5,6†], Yucheng Xue[1,2,3,4†], Xupeng Chai[1,2,3,4], Lingxiao Jin[1,2,3,4], Haochen Mou[1,2,3,4], Xiao Ma[1,2,3,4], Teng Ma[1,2,3,4], Liang Chen[1,2,3,4], Xiayu Hu[1,2,3,4], Kelei Wang[1,2,3,4], Senxu Lu[1,2,3,4], Fangqian Wang[1,2,3,4], Shenzhi Zhao[1,2,3,4], Minjun Yao[1,2,3,4], Chengcheng Yu[1,2,3,4], Junjie Pan[1,2,3,4], Xiaohua Yu[1,2,3,4*], Zhaoming Ye[1,2,3,4*] and Binghao Li[1,2,3,4*]*

[1]Department of Orthopedic Surgery, The Second Affiliated Hospital, Zhejiang University School of Medicine, Hangzhou, China

[2]Orthopedics Research Institute of Zhejiang University, Hangzhou, Zhejiang, China

[3]Key Laboratory of Motor System Disease Research and Precision Therapy of Zhejiang Province, Hangzhou, Zhejiang, China

[4]Clinical Research Center of Motor System Disease of Zhejiang Province, Hangzhou, Zhejiang, China

[5]Department of Obstetrics and Gynecology, The First Affiliated Hospital, Sun Yat-sen University, Guangzhou, Guangdong, China

[6]Guangdong Provincial Clinical Research Center for Obstetrical and Gynecological Diseases, Guangzhou, Guangdong, China

† These authors contributed equally to this work. * Corresponding authors.

CORRESPONDENCE

Zhenxuan Shao (zhenxuanshao@zju.edu.cn), Xiaohua Yu (Xhyu2936@gmail.com), Zhaoming Ye (yezhaoming@zju.edu.cn) and Binghao Li (libinghao@zju.edu.cn).

This PDF file includes:

Supplementary Figures 1–18

Supplementary Figure Legends

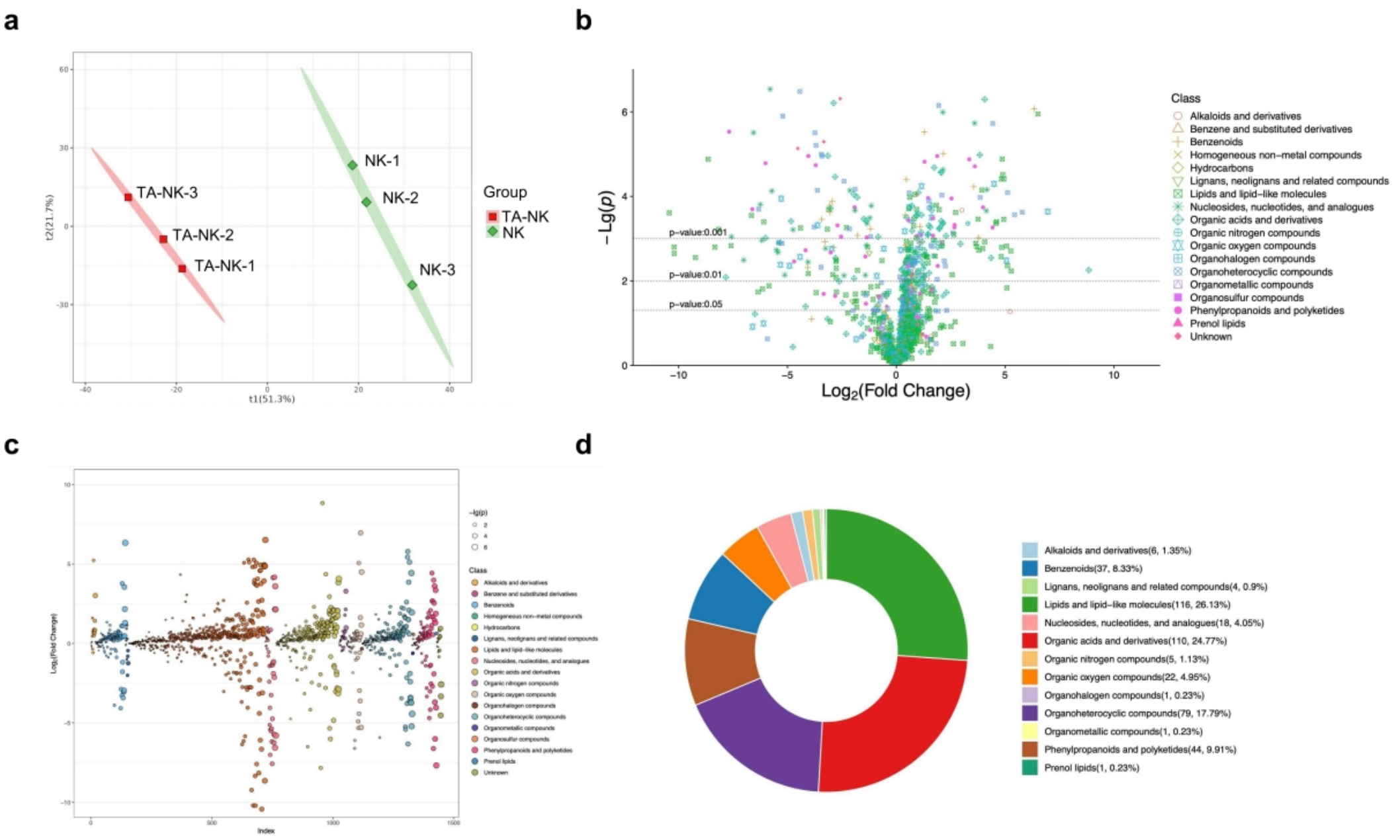

Supplementary Fig. 1 Untargeted metabolomics highlights lactate enrichment during NK-tumour interaction.

(a) Partial least-squares discriminant analysis score plot separating supernatants from NK-alone cultures and NK-tumour co-cultures; $n = 3$ independent culture supernatants per group, mean ± SD. (b) Volcano plot of significantly altered metabolites with variable importance in projection > 1 and $P < 0.05$. (c) Bubble plot showing pathway assignment and fold-change of the same metabolites. (d) Structural or functional classification of differential metabolites.

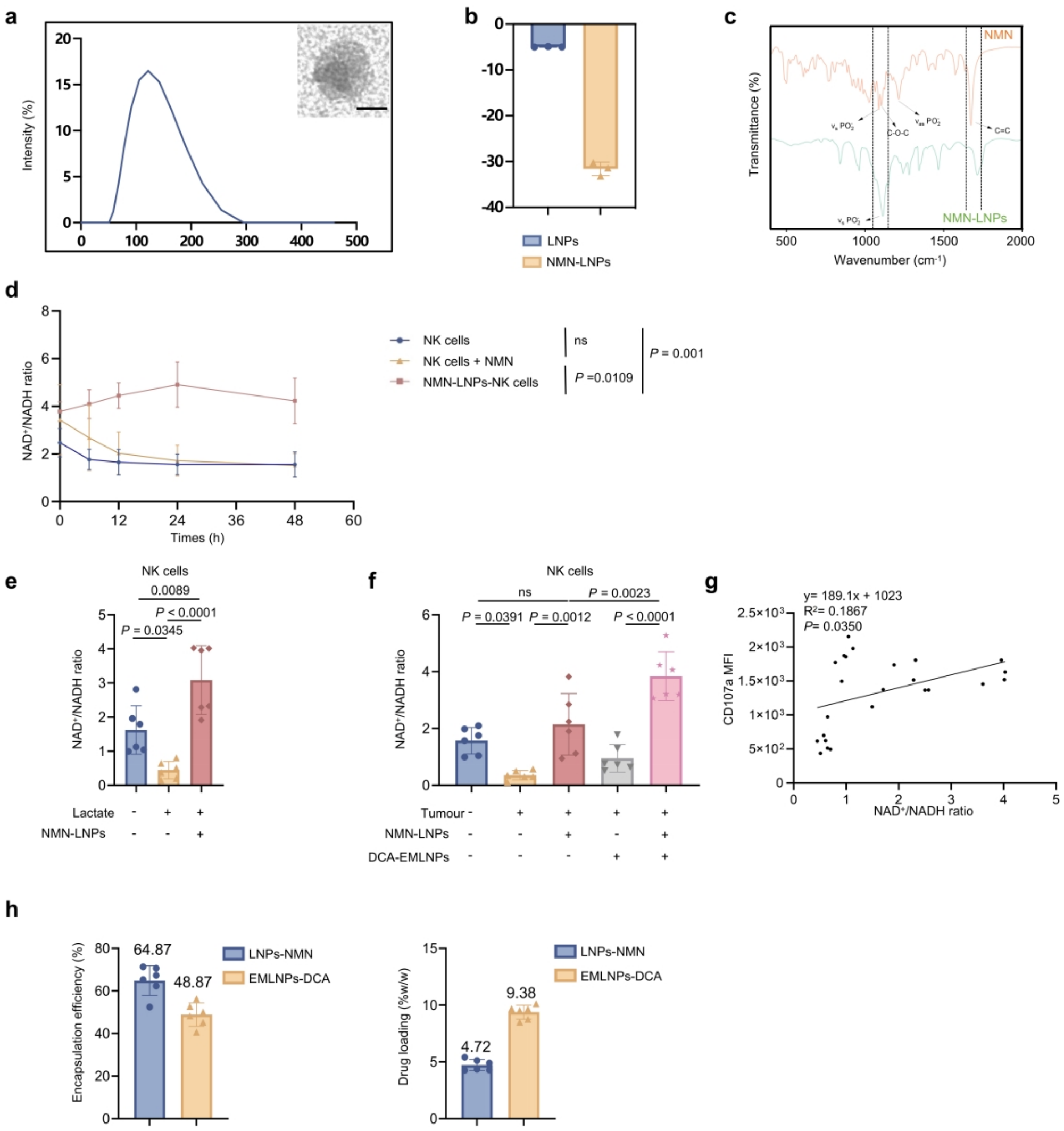

Supplementary Fig. 2 Physicochemical characterization of NMN-LNPs and NMN-LNP-mediated NAD⁺ restoration in NK cells.

(a) Dynamic light scattering size distribution of NMN-LNPs; inset, representative transmission electron microscopy image; scale bar, 50 nm. The TEM image is representative of three independently prepared NMN-LNP batches with similar results. (b) ζ-potential of blank LNPs and NMN-LNPs. (c) Fourier-transform infrared spectra of free NMN and NMN-LNPs. (d) Time-course of intracellular NAD^+/NADH ratio after free NMN or NMN-LNP treatment under identical washout handling; n = 6 independent NK-cell cultures per group measured longitudinally, mean ± SD;

two-way repeated-measures ANOVA with Sidak's test. (e) Intracellular NAD^+/NADH ratio in NK cells cultured with or without lactate and treated with NMN-LNPs; n = 6 independent NK-cell cultures per group, mean ± SD; one-way ANOVA with Tukey's test. (f) Intracellular NAD^+/NADH ratio in tumour-challenged NK cells treated with NMN-LNPs alone or with DCA-EMLNPs; n = 6 independent NK-cell cultures per group, mean ± SD; one-way ANOVA with Tukey's test. (g) Correlation between NK-cell NAD^+/NADH ratio and CD107a mean fluorescence intensity; two-tailed Pearson correlation. (h) Encapsulation efficiency and drug loading of NMN and DCA. Source data are provided as a Source Data file.

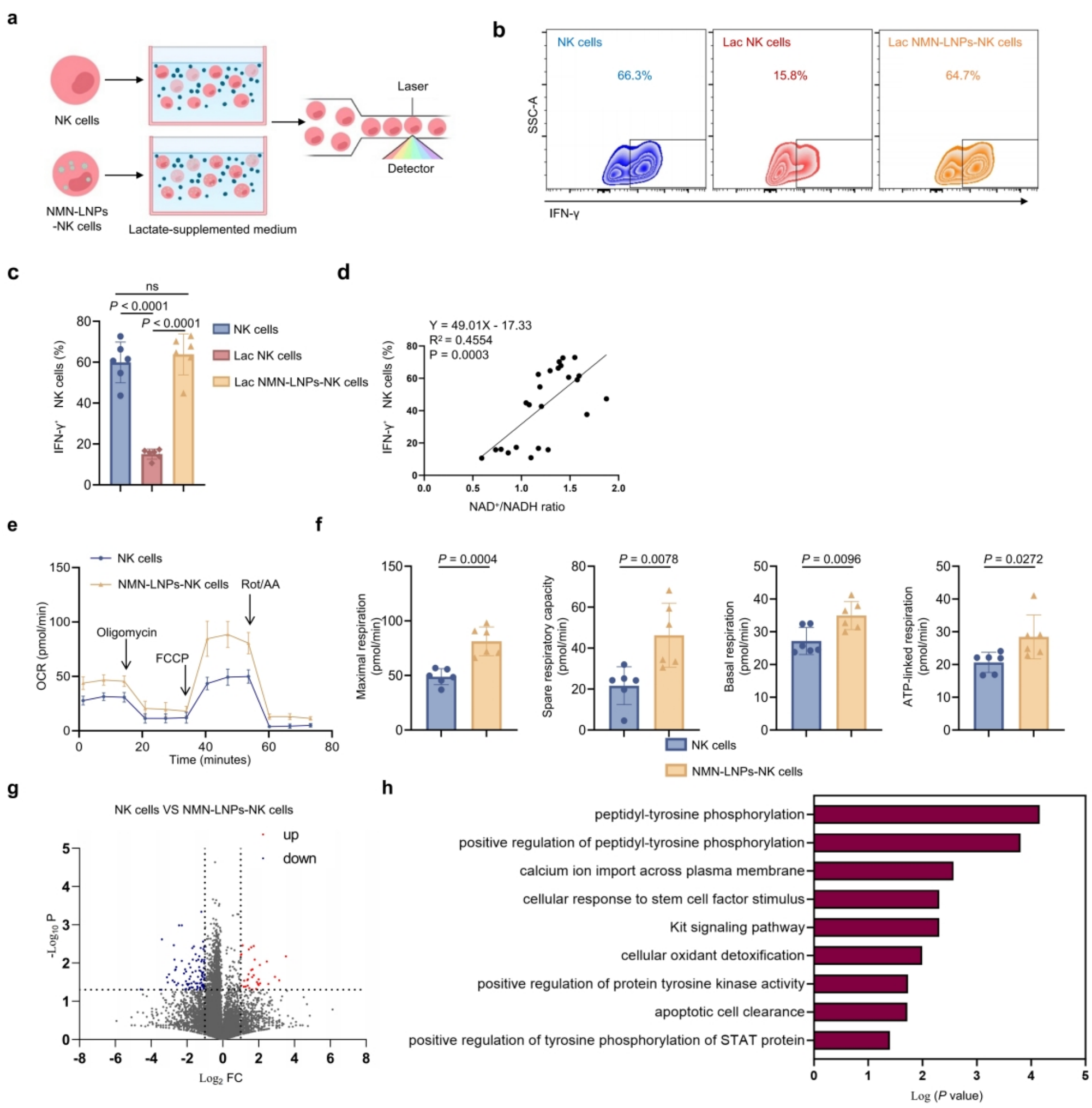

Supplementary Fig. 3 NMN-LNPs enhance NK-cell respiration and activation-associated transcriptional programmes.

(a) Experimental workflow. (b) Representative flow-cytometry plots of intracellular IFN-γ staining. (c) Quantification of IFN-γ^+ NK cells; n = 6 independent NK-cell cultures per group, mean ± SD; one-way ANOVA with Tukey's test. (d) Correlation between intracellular NAD^+/NADH ratio and IFN-γ^+ NK-cell frequency; two-tailed Pearson correlation. (e) Seahorse mitochondrial stress test showing oxygen consumption rate profiles of control and NMN-LNP-conditioned NK cells; n = 6 independent NK-cell cultures per group, mean ± SD. (f) Bioenergetic parameters derived from panel e; n = 6 independent NK-cell cultures per group, mean ± SD; two-tailed unpaired Student's t-test. (g) Volcano plot of differentially expressed genes after NMN-LNP conditioning. (h) Gene Ontology enrichment analysis of differentially expressed genes. Source data are provided as a Source Data file.

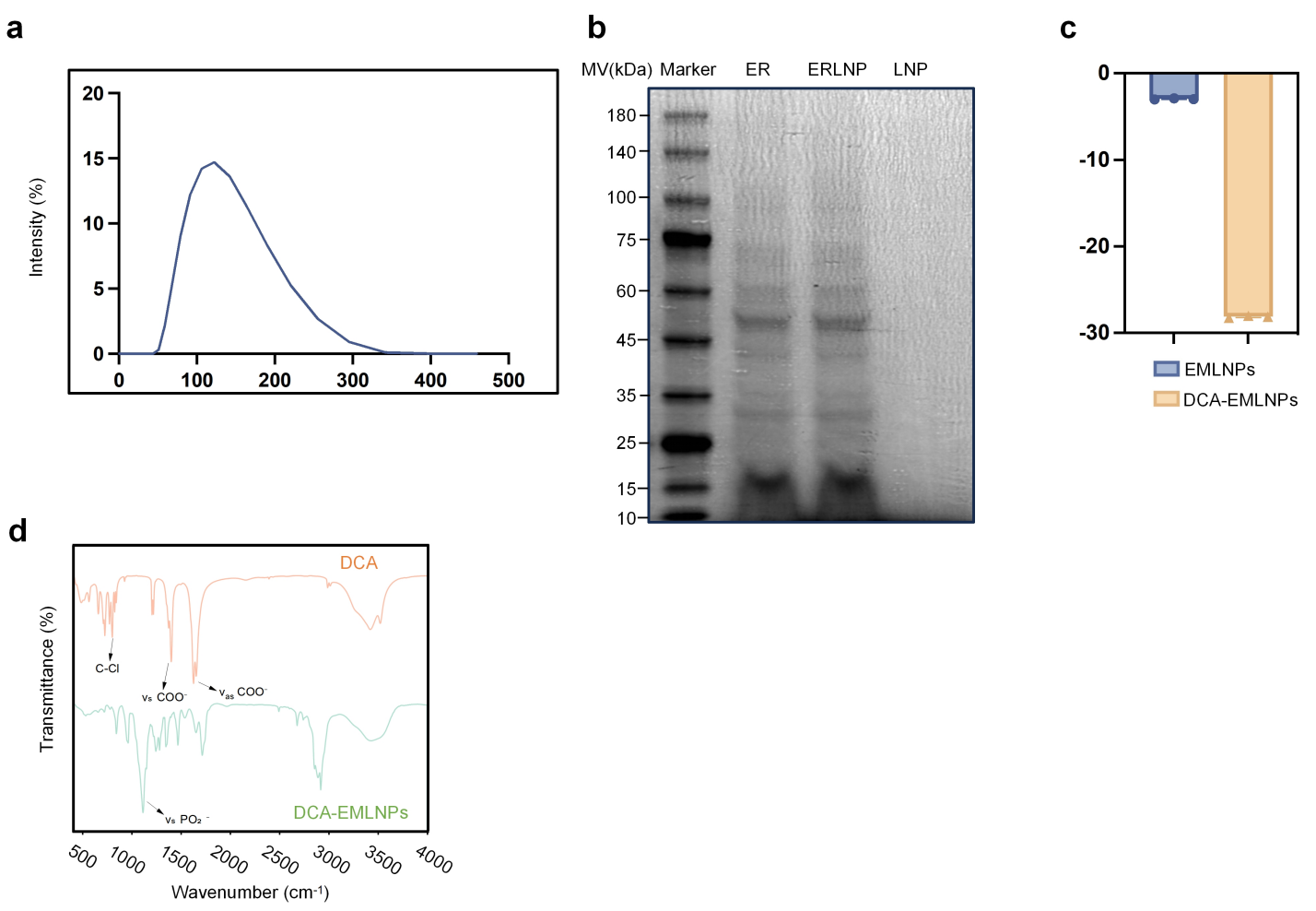

Supplementary Fig. 4 Preparation and characterisation of ER-hybrid lipid nanoparticles.

(a) Dynamic light scattering profile and transmission electron microscopy image of EMLNPs; scale bar, 100 nm. (b) Coomassie Blue gel showing ER-protein

incorporation into EMLNPs. (c) ζ-potential shift from blank EMLNPs to DCA-EMLNPs. (d) Fourier-transform infrared spectra of free DCA and DCA-EMLNPs. Source data are provided as a Source Data file.

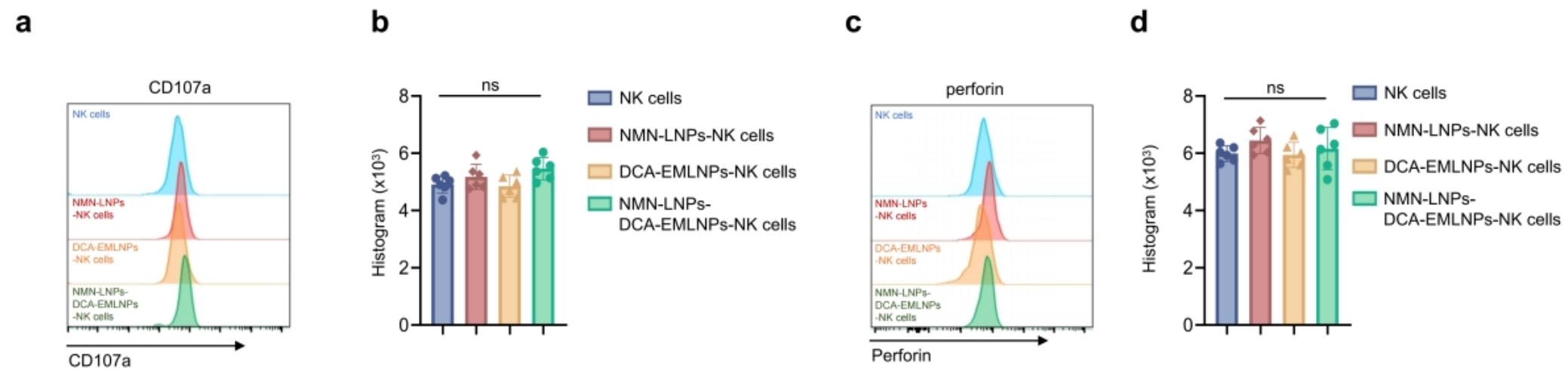

Supplementary Fig. 5 Dual-compartment loading does not perturb NK-cell cytolytic secretion.

(a,b) Representative flow-cytometry histograms and quantification of CD107a in untreated NK cells, NMN-LNPs-NK cells, DCA-EMLNPs-NK cells and NMN-LNPs-DCA-EMLNPs-NK cells. n = 6 independent NK-cell cultures per group, mean ± SD; one-way ANOVA with Tukey's test; ns, not significant. (c,d) Representative histograms and quantification of perforin in the same groups. n = 6 independent NK-cell cultures per group, mean ± SD; one-way ANOVA with Tukey's test; ns, not significant. Source data are provided as a Source Data file.

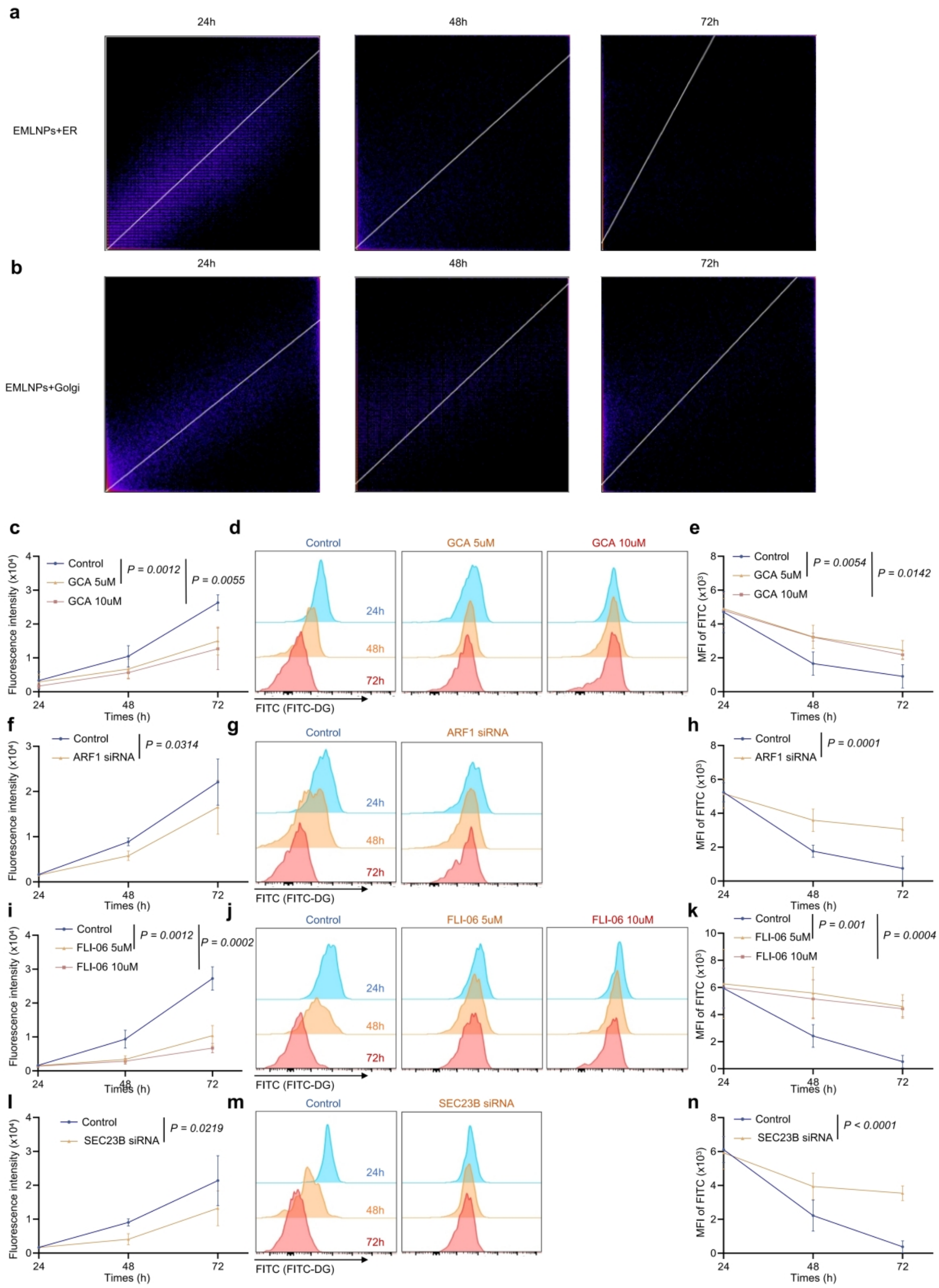

Supplementary Fig. 6 Perturbation analyses support ER-Golgi exocytic routing of EMLNP cargo.

(a,b) Pixel-correlation plots of EMLNP signal versus ER-marker or Golgi-marker fluorescence. (c-e) Golgicide A perturbation in the Transwell transfer assay; n = 6 independent NK-HOS Transwell co-cultures per group, mean ± SD for quantified

panels; two-way repeated-measures ANOVA with Sidak's test. (f-h) Coat protein complex I perturbation by ARF1 knockdown; n = 6 independent NK-HOS Transwell co-cultures per group, mean ± SD; two-way repeated-measures ANOVA with Sidak's test. (i-k) ER-exit blockade with FLI-06, with golgicide A as reference; n = 6 independent NK-HOS Transwell co-cultures per group, mean ± SD; two-way repeated-measures ANOVA with Sidak's test. (l-n) Coat protein complex II perturbation by SEC23B knockdown; n = 6 independent NK-HOS Transwell co-cultures per group, mean ± SD; two-way repeated-measures ANOVA with Sidak's test. Source data are provided as a Source Data file.

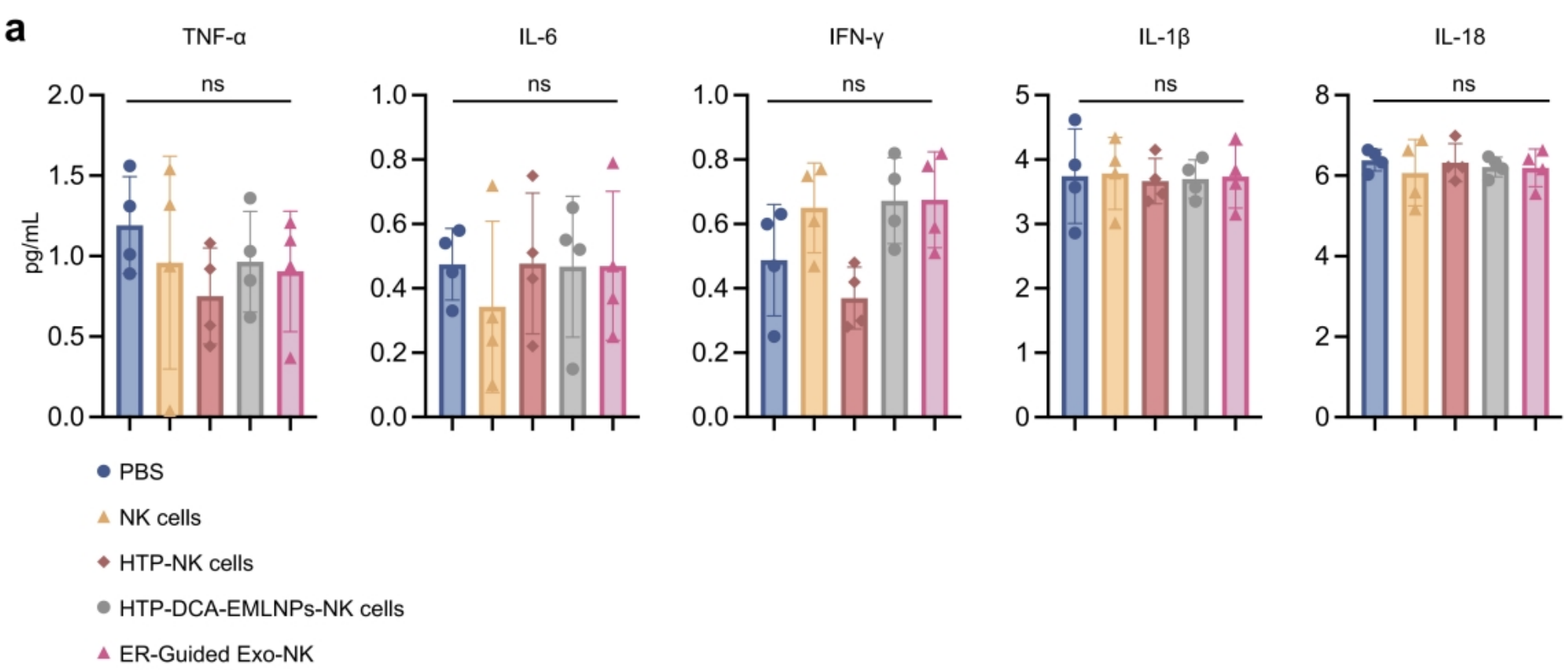

Supplementary Fig. 7 Serum cytokines after NK-cell therapies.

(a) Serum tumour necrosis factor-α, interleukin-6, IFN-γ, interleukin-1β and interleukin-18 concentrations 24 h after infusion of PBS, native NK, HTP-NK, HTP-DCA-EMLNPs-NK or ER-Guided Exo-NK; n = 4 mice per group, mean ± SD; one-way ANOVA with Tukey's test; ns, not significant. Source data are provided as a Source Data file.

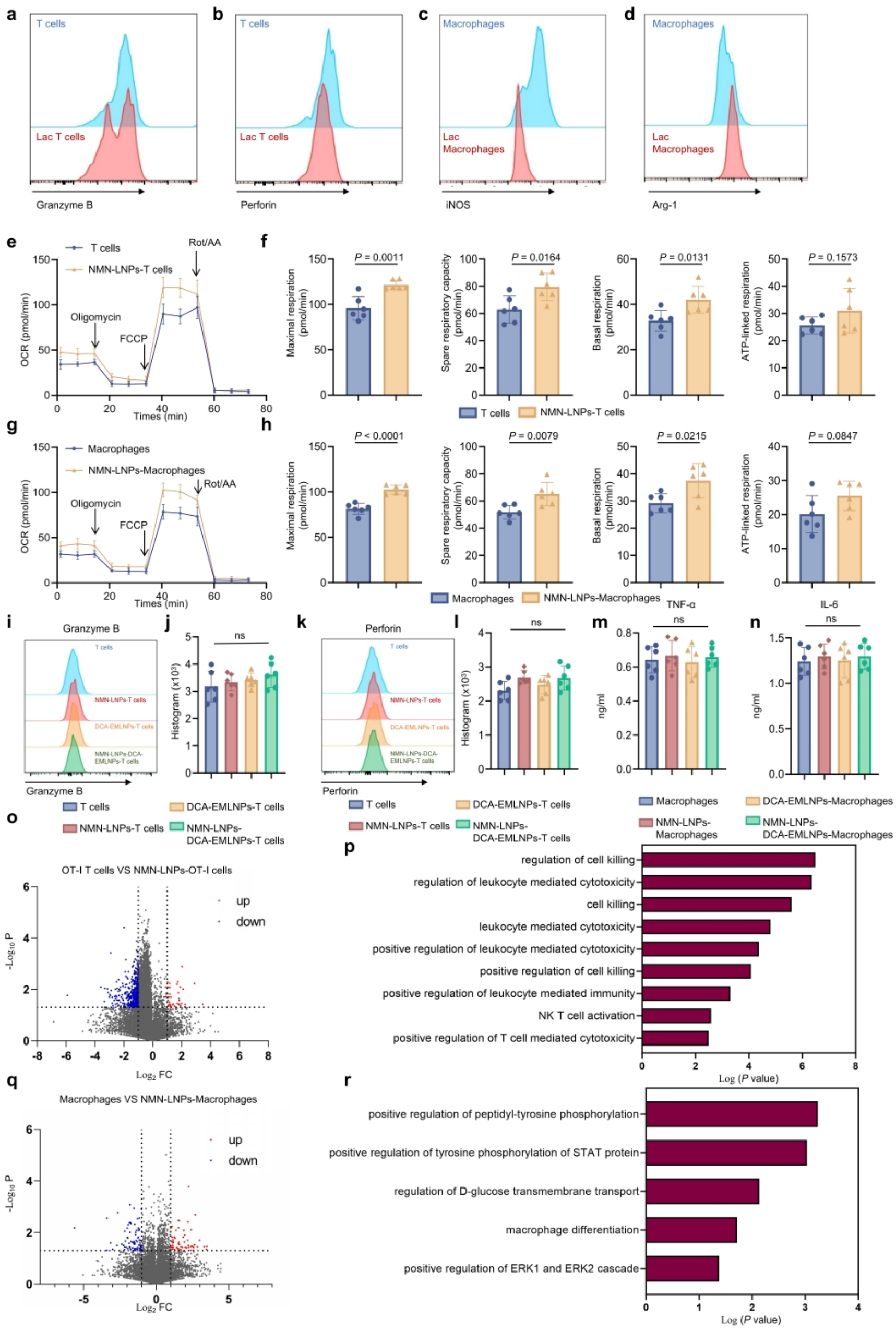

Supplementary Fig. 8 Lactate suppresses T-cell and macrophage effector programmes, while NMN-LNPs improve bioenergetic fitness.

(a-d) Representative histograms of Granzyme B, perforin, inducible nitric oxide synthase and arginase-1. In a-d, blue denotes standard medium and red denotes lactate-supplemented medium. (e) Oxygen consumption rate profiles of OT-I T cells with or without NMN-LNP conditioning; *n* = 6 independent OT-I $CD8^+$ T-cell cultures per group, mean ± SD. (f) Bioenergetic parameters from panel e; *n* = 6

independent OT-I CD8+ T-cell cultures per group, mean ± SD; two-tailed unpaired Student's t-test. (g) Oxygen consumption rate profiles of macrophages with or without NMN-LNP conditioning; n = 6 independent macrophage cultures per group, mean ± SD. (h) Bioenergetic parameters from panel g; n = 6 independent macrophage cultures per group, mean ± SD; two-tailed unpaired Student's t-test. (i-l) Granzyme B and perforin in untreated T cells, NMN-LNPs-T cells, DCA-EMLNPs-T cells and NMN-LNPs-DCA-EMLNPs-T cells; n = 6 independent OT-I CD8+ T-cell cultures per group, mean ± SD for quantified panels. (m,n) Tumour necrosis factor-α and interleukin-6 secretion from untreated macrophages, NMN-LNPs-macrophages, DCA-EMLNPs-macrophages and NMN-LNPs-DCA-EMLNPs-macrophages; n = 6 independent macrophage cultures per group, mean ± SD. Statistics for j, l, m and n: one-way ANOVA with Tukey's test; ns, not significant. (o-r) RNA sequencing volcano plots and Gene Ontology enrichment analyses after NMN-LNP conditioning. Source data are provided as a Source Data file.

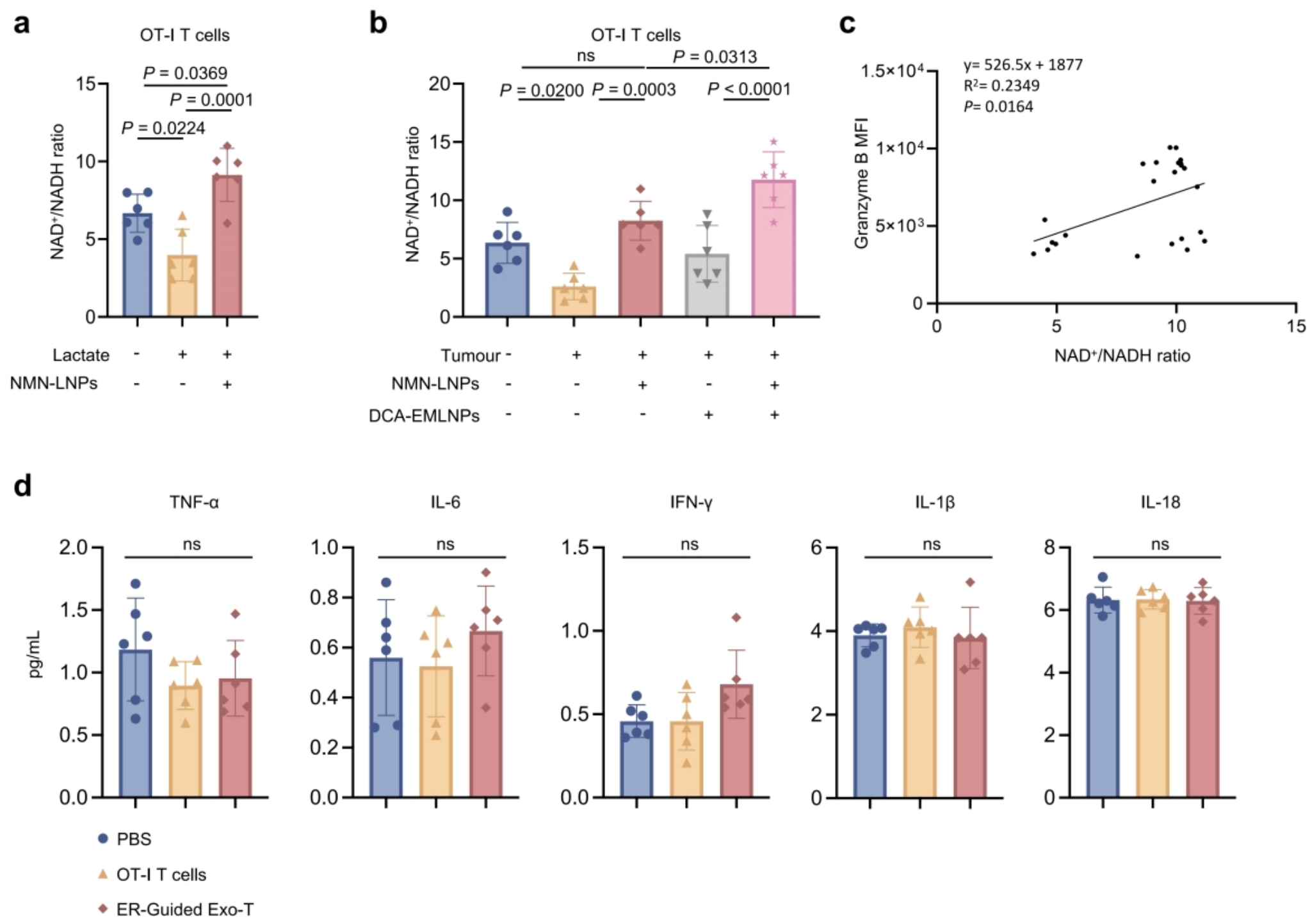

Supplementary Fig. 9 Serum cytokine profile after T-cell therapies.

(a) Intracellular NAD$^+$/NADH ratio in OT-I CD8$^+$ T cells cultured with or without lactate and treated with NMN-LNPs; n = 6 independent OT-I CD8$^+$ T-cell cultures per group, mean ± SD; one-way ANOVA with Tukey's test. (b) Intracellular NAD$^+$/NADH ratio in tumour-challenged OT-I T cells treated with NMN-LNPs alone or with DCA-EMLNPs; n = 6 independent OT-I CD8$^+$ T-cell cultures per group, mean ± SD; one-way ANOVA with Tukey's test. (c) Correlation between OT-I T-cell NAD$^+$/NADH ratio and Granzyme B mean fluorescence intensity; two-tailed Pearson correlation. (d) Serum tumour necrosis factor-α, interleukin-6, IFN-γ, interleukin-1β and interleukin-18 concentrations 24 h after PBS, OT-I T-cell or ER-Guided Exo-T infusion; n = 6 mice per group, mean ± SD; one-way ANOVA with Tukey's test; ns, not significant. Source data are provided as a Source Data file.

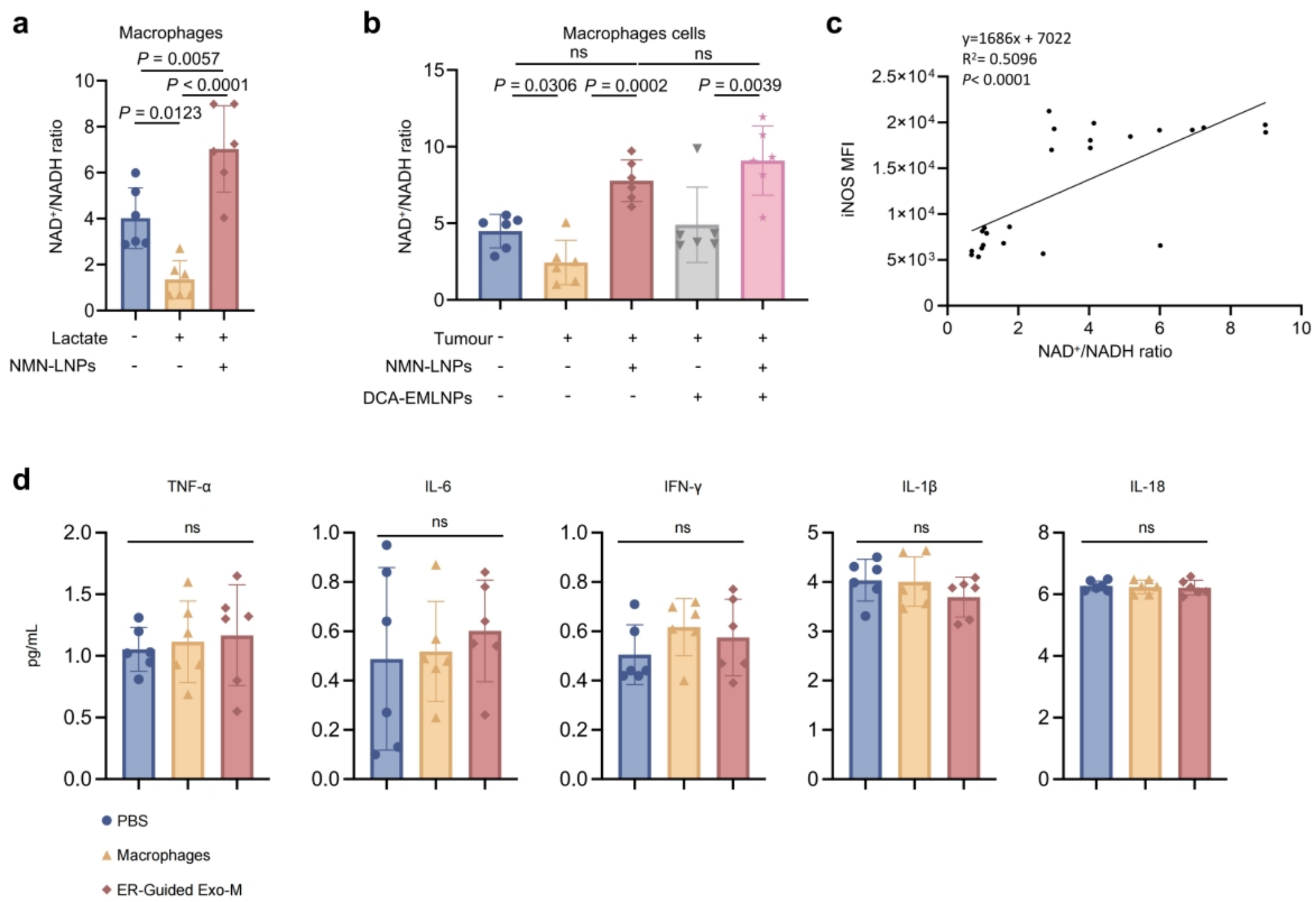

Supplementary Fig. 10 Serum cytokine profile after macrophage therapies.

(a) Intracellular NAD^+/NADH ratio in macrophages cultured with or without lactate and treated with NMN-LNPs; $n = 6$ independent macrophage cultures per group, mean ± SD; one-way ANOVA with Tukey's test. (b) Intracellular NAD^+/NADH ratio in tumour-challenged macrophages treated with NMN-LNPs alone or with DCA-EMLNPs; $n = 6$ independent macrophage cultures per group, mean ± SD; one-way ANOVA with Tukey's test. (c) Correlation between macrophage NAD^+/NADH ratio and inducible nitric oxide synthase mean fluorescence intensity; two-tailed Pearson correlation. (d) Serum tumour necrosis factor-α, interleukin-6, IFN-γ, interleukin-1β and interleukin-18 concentrations 24 h after PBS, macrophage or ER-Guided Exo-M infusion; $n = 6$ mice per group, mean ± SD; one-way ANOVA with Tukey's test; ns, not significant. Source data are provided as a Source Data file.

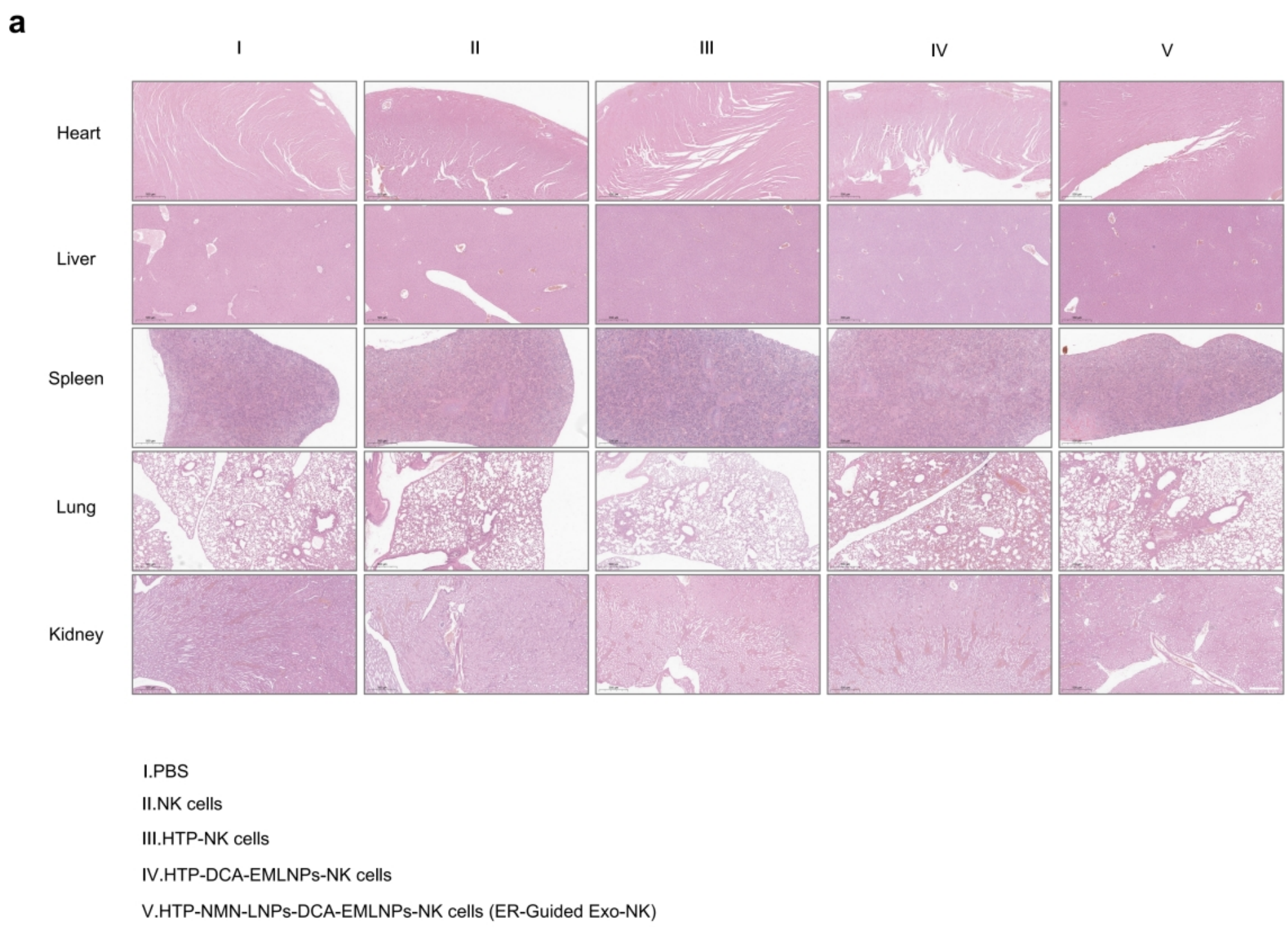

Supplementary Fig. 11 Histopathology of major organs after NK-cell therapies.

(a) Haematoxylin and eosin sections of heart, liver, spleen, lung and kidney from mice in the NK-cell therapy safety cohort shown in Supplementary Fig. 7. Scale bar, 500

μm. Representative H&E-stained sections are shown. Similar histopathological patterns were observed in n = 4 mice per group.

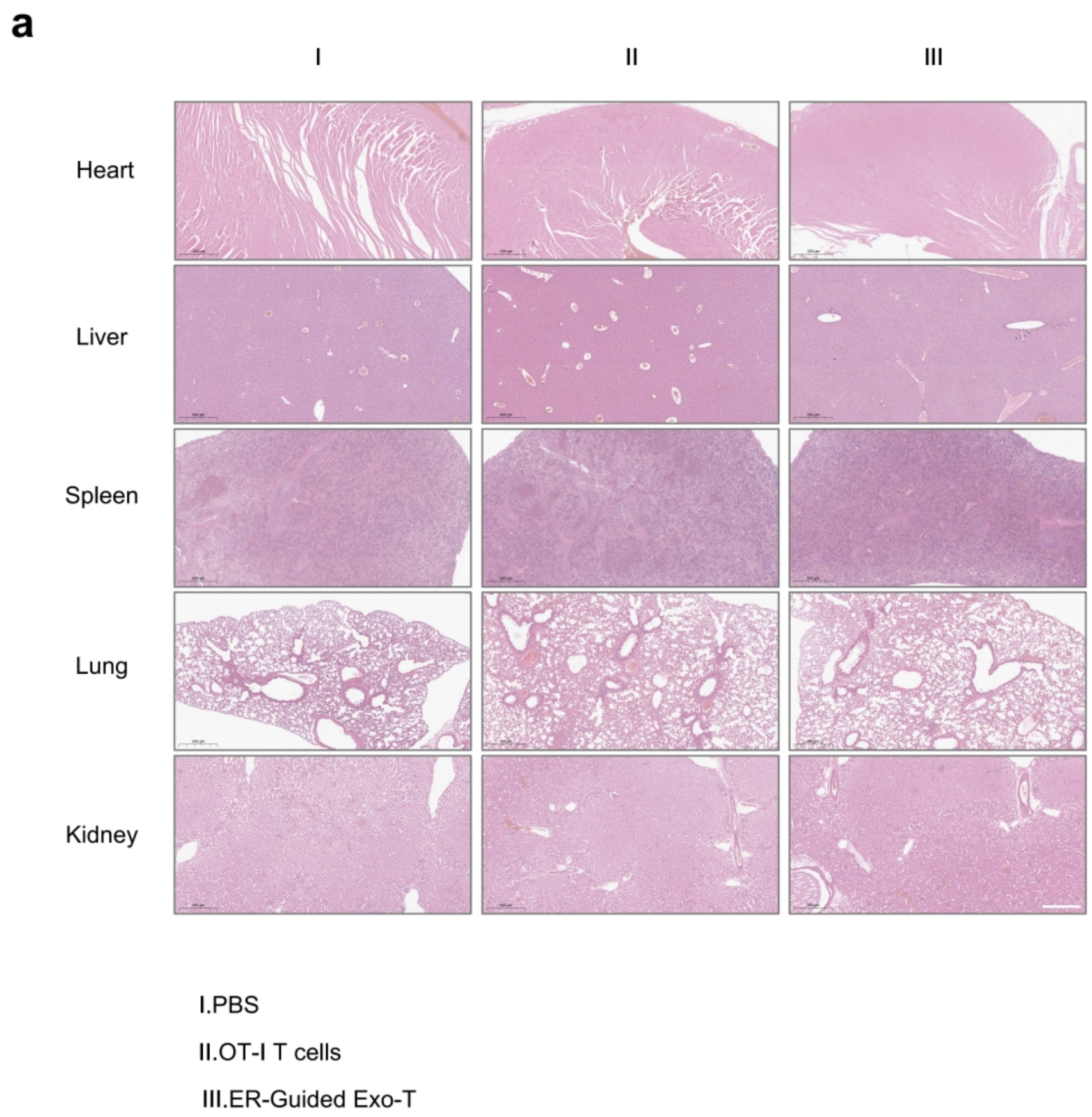

Supplementary Fig. 12 Histopathology of major organs after T-cell therapies.

(a) Haematoxylin and eosin sections of heart, liver, spleen, lung and kidney from mice in the T-cell therapy safety cohort shown in Supplementary Fig. 9d. Scale bar, 500 μm. Representative H&E-stained sections are shown. Similar histopathological patterns were observed in n = 6 mice per group.

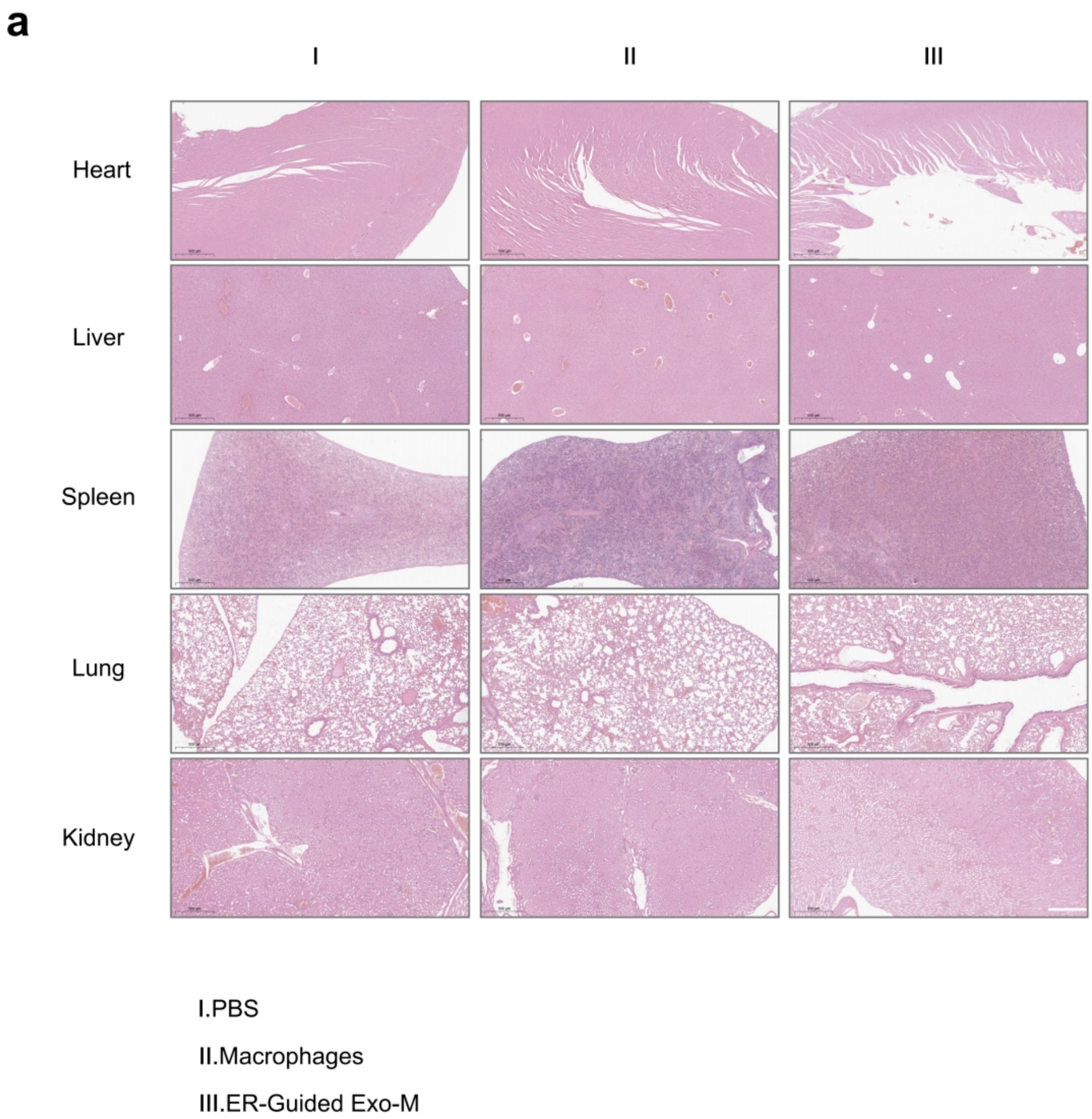

Supplementary Fig. 13 Histopathology of major organs after macrophage therapies.

(a) Haematoxylin and eosin sections of heart, liver, spleen, lung and kidney from mice in the macrophage therapy safety cohort shown in Supplementary Fig. 10d. Scale bar, 500 µm. Representative H&E-stained sections are shown. Similar histopathological patterns were observed in n = 6 mice per group.

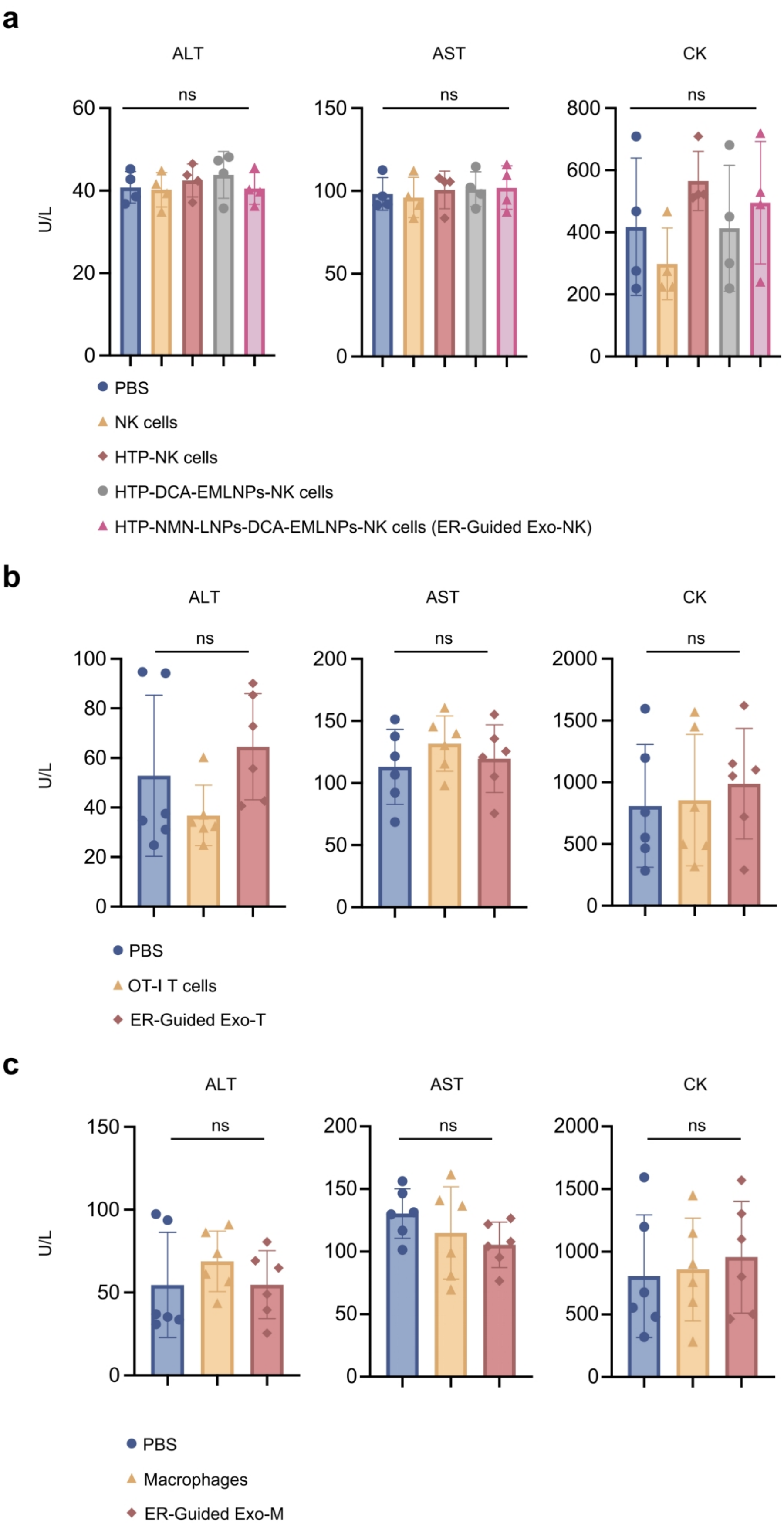
a
ALT
AST
CK
ns
U/L
60
40
20
0
150
100
50
800
600
400
200
PBS
NK cells
HTP-NK cells
HTP-DCA-EMLNPs-NK cells
HTP-NMN-LNPs-DCA-EMLNPs-NK cells (ER-Guided Exo-NK)
b
ALT
AST
CK
ns
U/L
100
80
60
40
20
0
200
150
100
50
2000
1500
1000
500
PBS
OT-I T cells
ER-Guided Exo-T
c
ALT
AST
CK
ns
U/L
150
100
50
0
200
2000
1500
1000
500
PBS
Macrophages
ER-Guided Exo-M

Supplementary Fig. 14 Serum chemistry following adoptive-cell treatments.

(a) Alanine aminotransferase, aspartate aminotransferase and creatine kinase after NK-cell therapies; n = 4 mice per group, mean ± SD; one-way ANOVA with Tukey's test; ns, not significant. (b) Alanine aminotransferase, aspartate aminotransferase and creatine kinase after T-cell therapies; n = 6 mice per group, mean ± SD; one-way ANOVA with Tukey's test; ns, not significant. (c) Alanine aminotransferase, aspartate aminotransferase and creatine kinase after macrophage therapies; n = 6 mice per group, mean ± SD; one-way ANOVA with Tukey's test; ns, not significant. Source data are provided as a Source Data file.

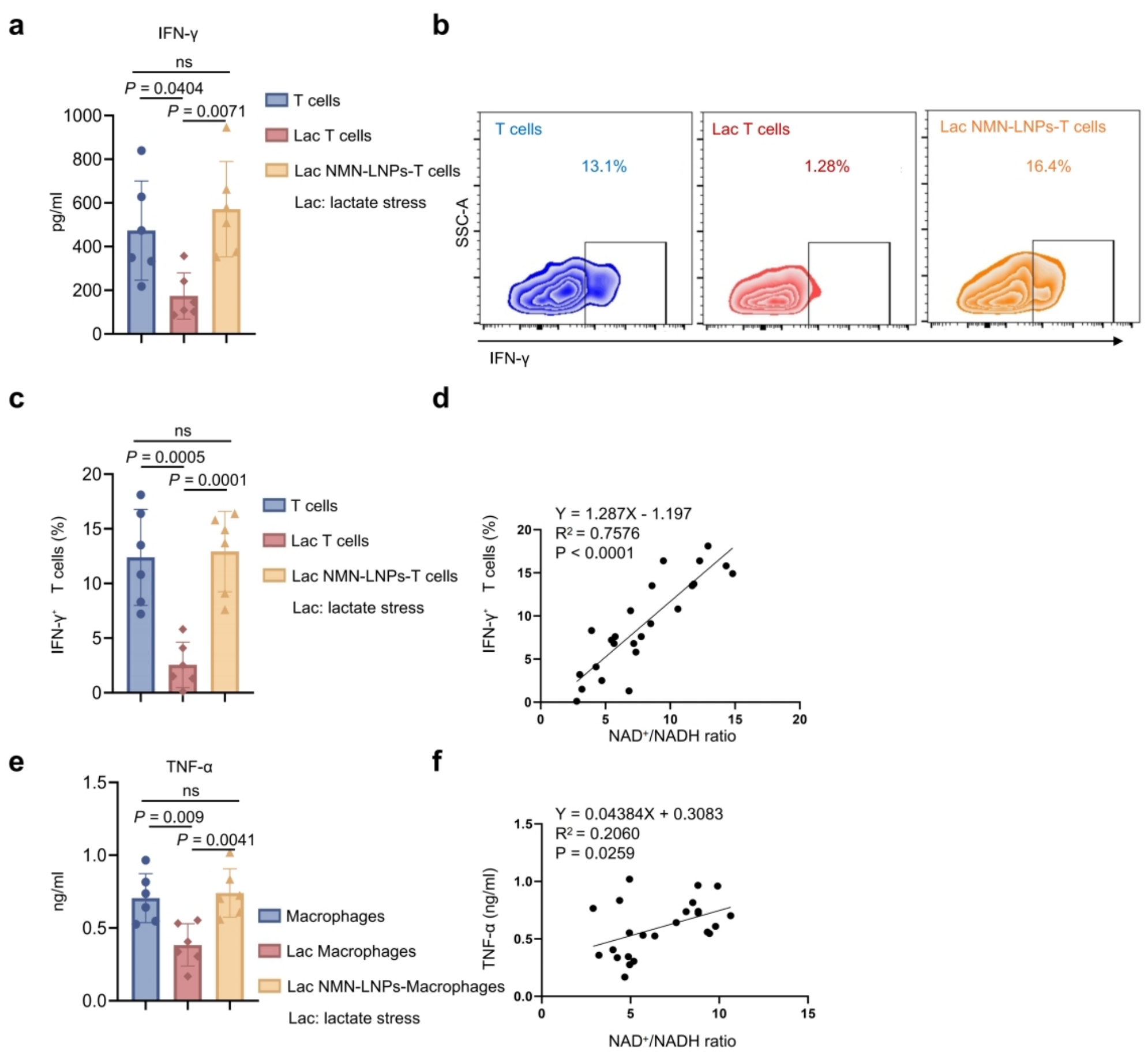

Supplementary Fig. 15 NMN-LNP-mediated NAD⁺ restoration improves T-cell IFN-γ and macrophage tumour necrosis factor-α output under lactate stress.

(a) IFN-γ in T-cell supernatants under control, lactate stress or lactate plus NMN-LNP treatment; $n = 6$ independent OT-I $CD8^+$ T-cell cultures per group, mean ± SD; one-way ANOVA with Tukey's test. (b) Representative flow-cytometry plots showing intracellular IFN-γ staining in T cells. (c) Quantification of IFN-γ^+ T cells; $n = 6$ independent OT-I $CD8^+$ T-cell cultures per group, mean ± SD; one-way ANOVA with Tukey's test. (d) Correlation between intracellular NAD^+/NADH ratio and IFN-γ^+ T-cell frequency; $n = 18$ independent culture-level observations pooled from three groups; two-tailed Pearson correlation. (e) Tumour necrosis factor-α in macrophage supernatants under control, lactate stress or lactate plus NMN-LNP treatment; $n = 6$ independent macrophage cultures per group, mean ± SD; one-way ANOVA with Tukey's test. (f) Correlation between intracellular NAD^+/NADH ratio and tumour necrosis factor-α concentration in macrophages; $n = 18$ independent culture-level observations pooled from three groups; two-tailed Pearson correlation; ns, not significant. Source data are provided as a Source Data file.

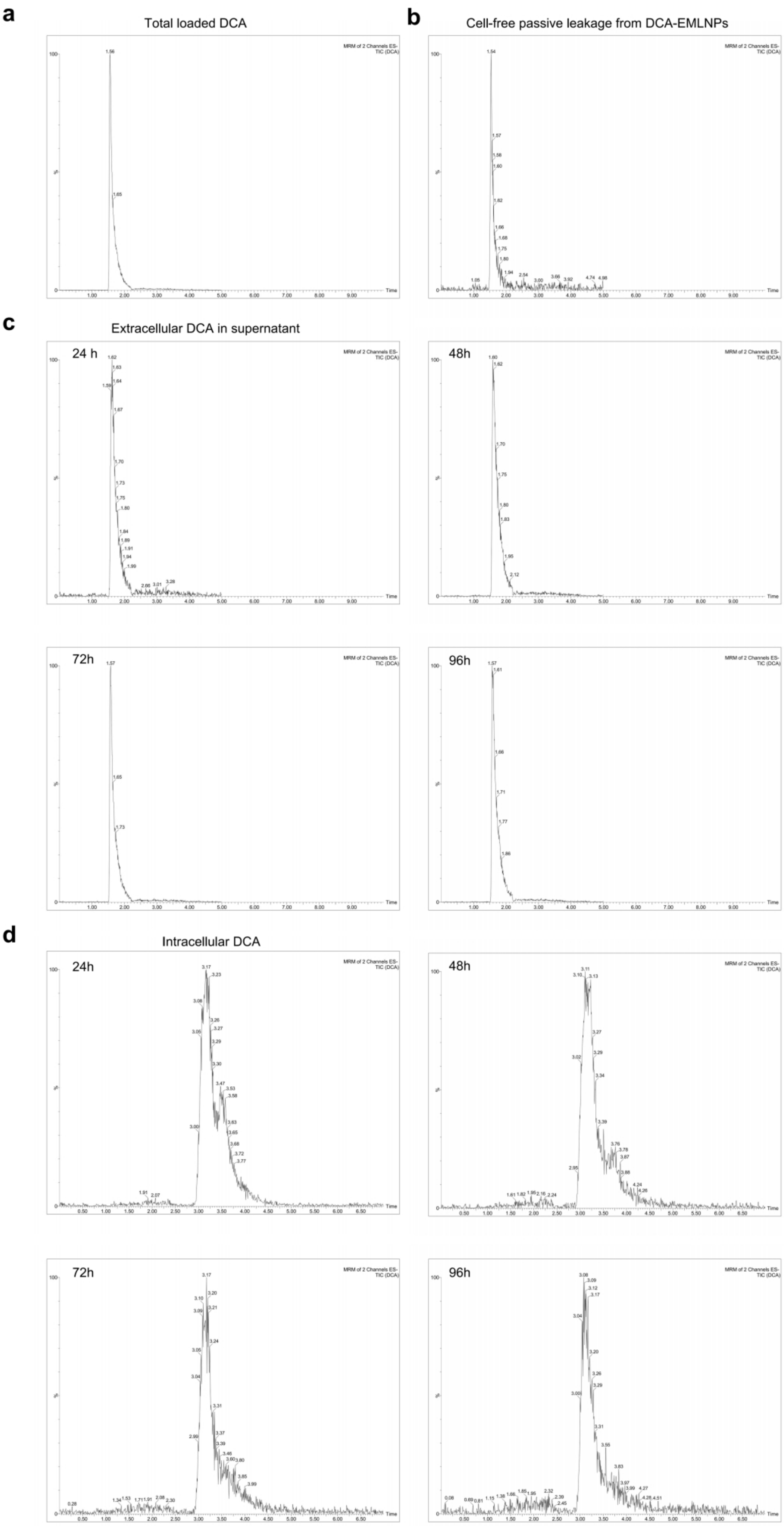
a
Total loaded DCA
b
Cell-free passive leakage from DCA-EMLNPs
c
Extracellular DCA in supernatant
24 h
48h
72h
96h
d
Intracellular DCA
24h
48h
72h
96h

Supplementary Fig. 16 LC-MS/MS validation of DCA distribution after DCA-EMLNP loading.

(a) Representative liquid chromatography-tandem mass spectrometry chromatogram of total loaded DCA after complete extraction from DCA-EMLNPs. (b) Representative chromatogram of DCA detected in supernatants from cell-free DCA-EMLNPs under passive-leakage conditions. (c) Representative chromatograms of extracellular DCA in culture supernatants from ER-Guided Exo-NK cells at 24, 48, 72 and 96 h. (d) Representative chromatograms of intracellular DCA in NK-cell lysates from ER-Guided Exo-NK cells at 24, 48, 72 and 96 h. Quantitative summaries are shown in Fig. 4i,j; *n* = 6 independent NK-cell cultures per time point for cell-associated measurements and *n* = 6 independent cell-free DCA-EMLNP incubations per time point for passive leakage. Source data are provided as a Source Data file.

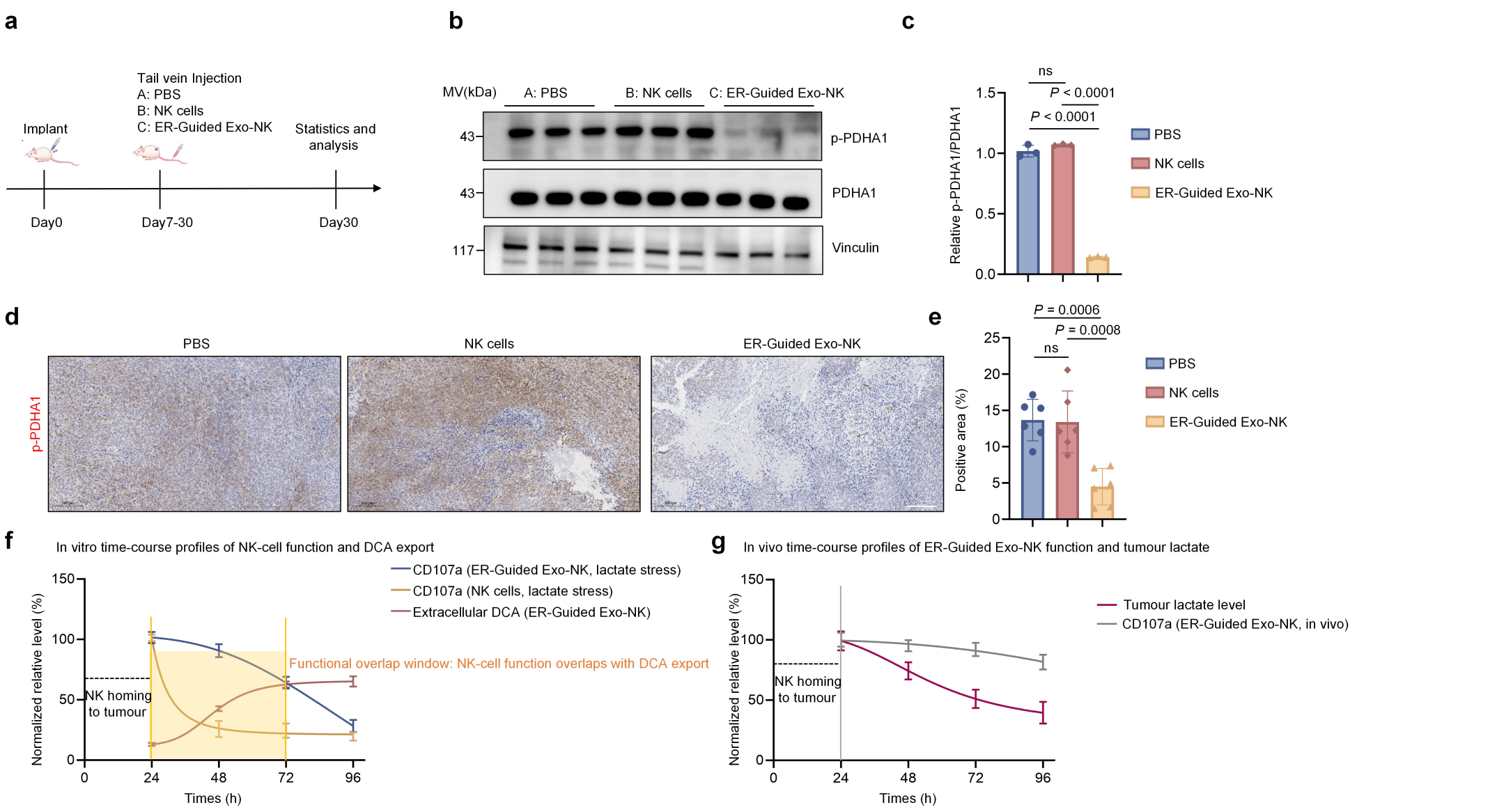

Supplementary Fig. 17 ER-Guided Exo-NK suppresses PDHA1 phosphorylation and temporally coordinates DCA export with NK-cell function.

(a) Workflow for in vivo assessment of pyruvate dehydrogenase E1α subunit phosphorylation after PBS, NK-cell or ER-Guided Exo-NK treatment in HOS tumour-bearing mice. (b) Western blot analysis of phosphorylated PDHA1 and total PDHA1; vinculin was the loading control. The western blot in b is representative of three tumour samples per group with similar results. (c) Quantification of phosphorylated PDHA1/total PDHA1 ratio; n = 3 tumours per group, mean ± SD; one-way ANOVA with Tukey's test; ns, not significant. (d) Representative phosphorylated PDHA1 immunohistochemistry in tumour sections; scale bar, 200 µm. The p-PDHA1 immunohistochemistry images in d are representative of n = 6 tumours per group with similar results. (e) Quantification of phosphorylated PDHA1-positive area; n = 6 tumours from 6 mice per group, mean ± SD; one-way ANOVA with Tukey's test; ns, not significant. (f) In vitro time-course profiles of NK-cell function and DCA export; CD107a profiles were derived from n = 4 independent NK-cell cultures per time point, and extracellular DCA measurements from n = 6 independent NK-cell cultures per time point; data are mean ± SD after normalization. (g) In vivo time-course profiles of ER-Guided Exo-NK function and tumour lactate; n = 6 mice per time point, mean ± SD after normalization. Source data are provided as a Source Data file.

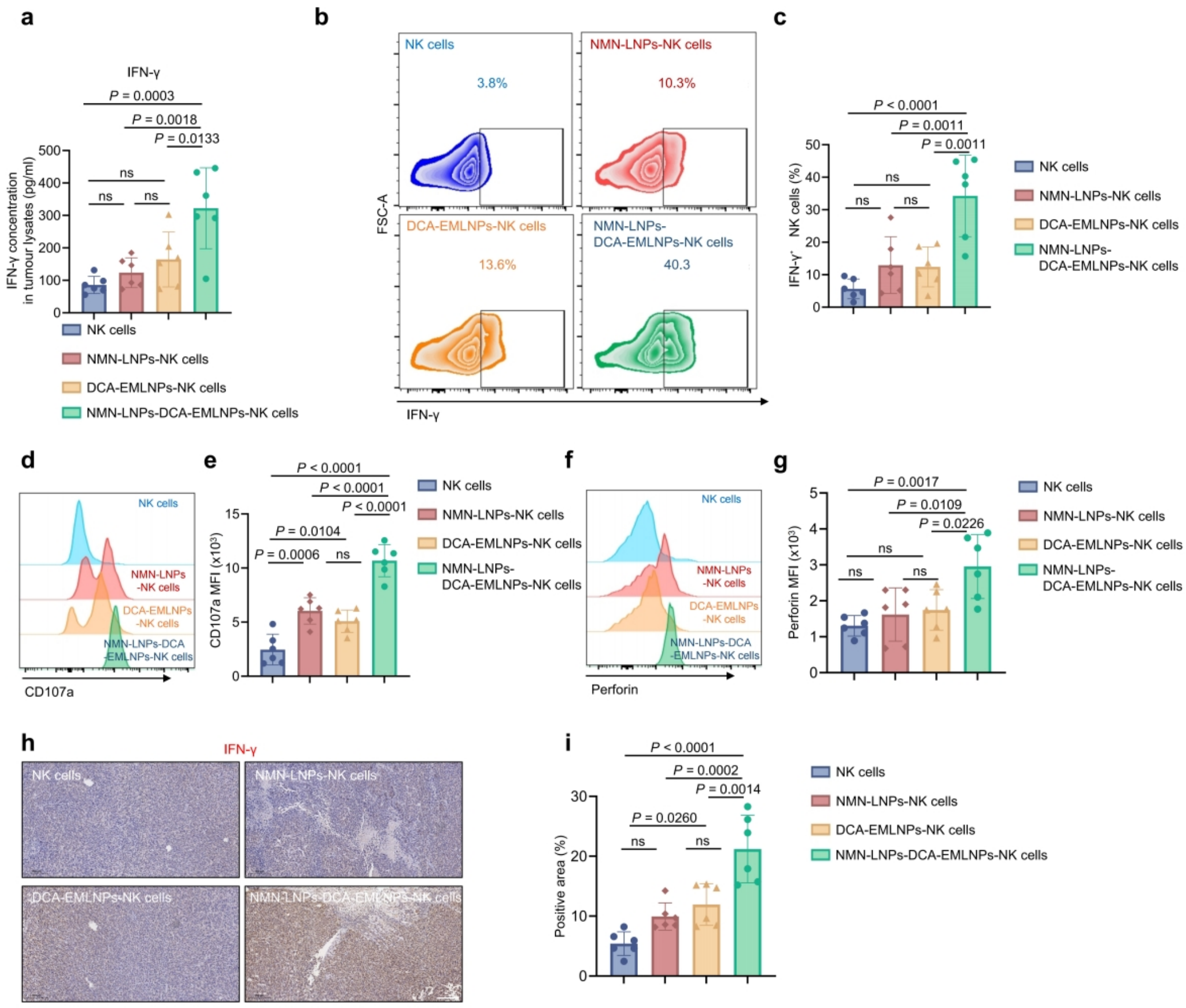

Supplementary Fig. 18 Component-dissection analysis of NMN-LNPs and DCA-EMLNPs in NK-cell therapy.

(a) IFN-γ concentration in tumour lysates from mice receiving the indicated NK-cell products; *n* = 6 tumours per group, mean ± SD; one-way ANOVA with Tukey's test; ns, not significant. (b) Representative flow-cytometry plots showing intracellular IFN-γ staining in NK cells. (c) IFN-γ^+ NK-cell quantification; *n* = 6 tumours per group, mean ± SD; one-way ANOVA with Tukey's test; ns, not significant. (d,e) Representative CD107a histograms and quantification; *n* = 6 tumours per group, mean ± SD; one-way ANOVA with Tukey's test; ns, not significant. (f,g) Representative perforin histograms and quantification; *n* = 6 tumours per group, mean ± SD; one-way ANOVA with Tukey's test; ns, not significant. (h) Representative IFN-γ immunohistochemistry in tumour sections; scale bar, 200 μm. (i)

Quantification of IFN-γ-positive area; $n = 6$ tumours from 6 mice per group, mean ± SD; one-way ANOVA with Tukey's test; ns, not significant. Source data are provided as a Source Data file.
