# Supplementary material for: Dual compartment lipid carriers hijack exocytosis to empower natural killer cells against solid tumours
Source: Nat Commun. 2026 Jul 21;17:8906. doi: 10.1038/s41467-026-75852-6 (PMC13503815; doi:10.1038/s41467-026-75852-6)
Supplement: Supplementary file 2 [file 41467_2026_75852_MOESM2_ESM.pdf]

nature portfolio

Corresponding author(s): Binghao Li

Last updated by author(s): May 8, 2026

Reporting Summary

Nature Portfolio wishes to improve the reproducibility of the work that we publish. This form provides structure for consistency and transparency in reporting. For further information on Nature Portfolio policies, see our Editorial Policies and the Editorial Policy Checklist.

Statistics

For all statistical analyses, confirm that the following items are present in the figure legend, table legend, main text, or Methods section.

n/a	Confirmed	
☐	☒	The exact sample size (*n*) for each experimental group/condition, given as a discrete number and unit of measurement
☐	☒	A statement on whether measurements were taken from distinct samples or whether the same sample was measured repeatedly
☐	☒	The statistical test(s) used AND whether they are one- or two-sided<br>*Only common tests should be described solely by name; describe more complex techniques in the Methods section.*
☒	☐	A description of all covariates tested
☐	☒	A description of any assumptions or corrections, such as tests of normality and adjustment for multiple comparisons
☐	☒	A full description of the statistical parameters including central tendency (e.g. means) or other basic estimates (e.g. regression coefficient) AND variation (e.g. standard deviation) or associated estimates of uncertainty (e.g. confidence intervals)
☐	☒	For null hypothesis testing, the test statistic (e.g. *F*, *t*, *r*) with confidence intervals, effect sizes, degrees of freedom and *P* value noted<br>*Give P values as exact values whenever suitable.*
☒	☐	For Bayesian analysis, information on the choice of priors and Markov chain Monte Carlo settings
☐	☒	For hierarchical and complex designs, identification of the appropriate level for tests and full reporting of outcomes
☒	☐	Estimates of effect sizes (e.g. Cohen's *d*, Pearson's *r*), indicating how they were calculated

Our web collection on statistics for biologists contains articles on many of the points above.

Software and code

Policy information about availability of computer code

Data collection	Data collection: Flow-cytometry data were collected using a Beckman Coulter CytoFLEX LX flow cytometer. Fluorescence microscopy images were acquired using Leica fluorescence microscopes, confocal images were acquired using a Leica TCS SP8 confocal microscope, whole-animal fluorescence images were acquired using an IVIS Spectrum imaging system (PerkinElmer), Seahorse metabolic data were collected using an Agilent Seahorse XF analyser, and LC-MS/MS data were acquired using instrument-control software associated with the Waters UPLC I-class/ AB Sciex TripleTOF 5600+ and Waters UPLC/XEVO-TQD systems. No custom data-collection software was used.
Data analysis	Data analysis: Flow-cytometry data were analysed using FlowJo v10. Statistical analyses were performed using GraphPad Prism 9.0.0. Image quantification and densitometry were performed using ImageJ v1.53. RNA-seq data were processed using fastp v0.20.1, HISAT2 v2.1.0, StringTie v1.3.3b and edgeR v3.20.9. Gene Ontology and pathway enrichment analyses were performed using DAVID v6.8 and GSEA v4.3.3. Untargeted metabolomics data were processed using MS-DIAL ver. 4.9 and Python v3.8. Seahorse data were analysed using Seahorse Wave v2.6. Public survival analyses were performed using the Bioinfo-ZS web platform. No custom code or custom algorithms were used in this study.

For manuscripts utilizing custom algorithms or software that are central to the research but not yet described in published literature, software must be made available to editors and reviewers. We strongly encourage code deposition in a community repository (e.g. GitHub). See the Nature Portfolio guidelines for submitting code & software for further information.

Data

Policy information about availability of data

All manuscripts must include a data availability statement. This statement should provide the following information, where applicable:

- Accession codes, unique identifiers, or web links for publicly available datasets
- A description of any restrictions on data availability
- For clinical datasets or third party data, please ensure that the statement adheres to our policy

The bulk RNA-seq datasets generated in this study have been deposited in the NCBI Sequence Read Archive under BioProject accession codes PRJNA1391857 [https://www.ncbi.nlm.nih.gov/sra/?term=PRJNA1391857], PRJNA1391541 [https://www.ncbi.nlm.nih.gov/sra/?term=PRJNA1391541] and PRJNA1391664 [https://www.ncbi.nlm.nih.gov/sra/?term=PRJNA1391664]. The LC-MS-based untargeted metabolomics dataset has been deposited in BIG Sub OMIX under accession code OMIX011385 [https://ngdc.cncb.ac.cn/omix/release/OMIX011385]. OMIX011385 is available under controlled access because it contains human biospecimen-associated omics data subject to participant consent, institutional ethics approval and China's Human Genetic Resources regulations. Access can be requested through the OMIX controlled-access portal. The TCGA-SARC [https://portal.gdc.cancer.gov/projects/TCGA-SARC] and TARGET-OS [https://portal.gdc.cancer.gov/projects/TARGET-OS] datasets used for survival analyses are publicly available through the Genomic Data Commons Data Portal. The full Data Availability statement is provided in the manuscript.

Research involving human participants, their data, or biological material

Policy information about studies with human participants or human data. See also policy information about sex, gender (identity/presentation), and sexual orientation and race, ethnicity and racism.

Reporting on sex and gender	Sex and gender were not used as grouping variables in this study. For PBMC-derived biospecimens provided as de-identified materials, donor sex and gender information was not available to the investigators. For patient-derived tumour tissues used to establish PDX models, available clinical sex information was obtained from clinical records. The five PDX donor participants were male. Gender identity was not collected. Sex- and gender-based analyses were not performed because the study was designed to evaluate engineered immune-cell function and antitumour efficacy rather than sex- or gender-specific effects.
Reporting on race, ethnicity, or other socially relevant groupings	Race, ethnicity, and other socially relevant groupings were not collected and were not used in analyses.
Population characteristics	Human biospecimens consisted of de-identified peripheral blood-derived PBMCs used for NK-cell isolation and patient-derived tumour tissues used for PDX establishment. Participant-level demographic information for PBMC donors, including age, sex and gender, was not available to the investigators. Patient-derived tumour tissues used for PDX establishment were obtained from five participants, including four participants with osteosarcoma and one participant with metastatic squamous cell carcinoma. The five PDX donor participants were male, with ages ranging from 12 to 60 years. Age, sex, gender, race and ethnicity were not used as grouping variables or covariates because the study was designed to evaluate engineered immune-cell function and antitumour efficacy rather than participant-level covariate effects.
Recruitment	PBMC biospecimens were obtained as de-identified samples provided by Sunray Biotechnology. For PDX establishment, patient-derived tumour tissues were collected from participants recruited at the Department of Orthopaedics, The Second Affiliated Hospital, Zhejiang University School of Medicine, under institutional ethics approval and with written informed consent. The PDX cohort included four osteosarcoma specimens and one metastatic squamous cell carcinoma specimen. No compensation or inducements were provided by the investigators.
Ethics oversight	The Second Affiliated Hospital, Zhejiang University School of Medicine (Approval (2024) Ethics Review (Research) No. (0735)). Collection and use of patient tumour tissues for PDX establishment were approved by the same institution (Approval (2021) Ethics Review (Research) No. (0977)). Written informed consent was obtained from all participants (or from their legal guardians) prior to sample collection. All human materials were handled as de-identified samples, and no identifiable private information was accessed by the investigators.This study was conducted in accordance with the Declaration of Helsinki

Note that full information on the approval of the study protocol must also be provided in the manuscript.

Field-specific reporting

Please select the one below that is the best fit for your research. If you are not sure, read the appropriate sections before making your selection.

☒ Life sciences ☐ Behavioural & social sciences ☐ Ecological, evolutionary & environmental sciences

For a reference copy of the document with all sections, see nature.com/documents/nr-reporting-summary-flat.pdf

Life sciences study design

All studies must disclose on these points even when the disclosure is negative.

Sample size	No statistical method was used to predetermine sample size. Sample sizes were selected based on commonly used experimental designs in related preclinical nanomedicine and tumour-immunotherapy studies, while taking into account experimental feasibility, animal ethics and expected biological effect size. Exact sample sizes are provided in the corresponding figure legends and Source Data.
Data exclusions	No animals or biological samples were excluded from the analyses. All collected biological replicates were included as reported. For LC-MS-

based untargeted metabolomics, ion features with >50% missing values within a group were removed as a prespecified feature-level data-processing step before downstream statistical analysis.

Replication

Key experimental findings were verified by independent replication and showed consistent results across repeats. In vitro experiments were performed as independent biological replicates on different days using independent cell preparations, with technical replicates averaged within each experiment before statistical analysis. In vivo experiments were reproduced in independent cohorts of mice where applicable. Exact replicate numbers and sample sizes are provided in the corresponding figure legends, Methods and Source Data.

Randomization

For in vivo therapeutic experiments, animals were allocated to experimental groups after tumour establishment. Once tumours reached comparable sizes, mice were allocated using a random-number table or randomized with pretreatment tumour volumes balanced between groups where possible. For patient-derived xenograft experiments, mice were randomized into treatment groups when tumours reached the predefined volume range. In vitro samples were processed in parallel under identical conditions, and where feasible, the handling order was alternated to minimize potential batch effects. Human biospecimens were not allocated to interventional groups; de-identified PBMC samples and patient-derived tumour tissues were used according to sample availability and analysed under predefined experimental conditions.

Blinding

Blinding was not performed. Most endpoints were objective or instrument-based, including tumour-volume measurements, flow cytometry using predefined gating strategies and controls, LC-MS/MS quantification, luminescence or absorbance-based metabolic assays and automated or predefined image quantification. Analyses were conducted using predefined criteria to minimize potential bias.

Reporting for specific materials, systems and methods

We require information from authors about some types of materials, experimental systems and methods used in many studies. Here, indicate whether each material, system or method listed is relevant to your study. If you are not sure if a list item applies to your research, read the appropriate section before selecting a response.

Materials & experimental systems

n/a	Involved in the study	
	☒	Antibodies
	☒	Eukaryotic cell lines
☒		Palaeontology and archaeology
	☒	Animals and other organisms
	☒	Clinical data
☒		Dual use research of concern
☒		Plants

Methods

n/a	Involved in the study	
☒		ChIP-seq
	☒	Flow cytometry
☒		MRI-based neuroimaging

Antibodies

Antibodies used

BioLegend, Brilliant Violet 510 anti-mouse CD45, Cat# 103137, clone 30-F11
BioLegend, APC anti-mouse NK-1.1, Cat# 108709, clone PK136
BioLegend, FITC anti-mouse CD107a (LAMP-1), Cat# 121605, clone 1D4B
BioLegend, APC/Fire 750 anti-mouse Perforin, Cat# 154317, clone S16009A
BioLegend, PerCP anti-mouse/human CD11b, Cat# 101229, clone M1/70
BioLegend, FITC anti-mouse F4/80, Cat# 123107, clone BM8
BioLegend, PE/Cyanine7 anti-Nos2 (iNOS), Cat# 696813, clone W16030C
BioLegend, PE anti-mouse Arginase 1, Cat# 165803, clone W21047I
BioLegend, APC anti-mouse CD8a, Cat# 162305, clone S18018E
BioLegend, Brilliant Violet 421 anti-human CD3, Cat# 317343, clone OKT3
BioLegend, PE anti-human CD56, Cat# 318306, clone HCD56
BioLegend, Brilliant Violet 510 anti-human CD45, Cat# 368525, clone 2D1
BioLegend, FITC anti-human CD107a, Cat# 328605, clone H4A3
BioLegend, Alexa Fluor 647 anti-human/mouse Granzyme B, Cat# 515405, clone GB11
BioLegend, APC/Cyanine7 anti-human Perforin, Cat# 308127, clone dG9
Abcam, anti-human CD56, Cat# ab75813, clone EP2567Y
Cell Signaling Technology, Ki-67, Cat# 9449, clone 8D5
BioLegend, Brilliant Violet 711 anti-human IFN-γ, Cat# 502539, clone 4S.B3
BioLegend, Brilliant Violet 711 anti-mouse IFN-γ, Cat# 505835, clone XMG1.2
Abcam, anti-phospho-PDHA1, Cat# ab177461, clone EPR12200
Abcam, anti-PDHA1, Cat# ab168379, clone EPR11098
Abcam, anti-Vinculin, Cat# ab129002, clone EPR8185
Abcam, anti-IFN-γ, Cat# ab231036, clone EPR21704

Validation

Antibodies were used for the indicated species and applications based on the manufacturers' validation information provided in the product datasheets and supported by in-study controls. For flow cytometry, antibody performance was assessed using single-stain compensation controls and fluorescence-minus-one controls, and gating was based on canonical marker combinations. Intracellular staining was performed after fixation and permeabilization according to the manufacturers' instructions, with appropriate negative controls. For immunohistochemistry, including CD56/NCAM1, Ki-67, IFN-γ and phospho-PDHA1 staining, antibody specificity was supported by the expected staining pattern and no-primary-antibody negative controls. For western blotting, phospho-PDHA1, total

PDHA1 and Vinculin antibodies were used according to the manufacturers' validated applications, and bands were interpreted based on the expected molecular weights.

Eukaryotic cell lines

Policy information about cell lines and Sex and Gender in Research

Cell line source(s)	HOS human osteosarcoma cells were obtained from Procell Life Science & Technology (CL-0360). MC38 murine colon adenocarcinoma cells were obtained from the Cell Bank of the Chinese Academy of Sciences (Shanghai, China; SCSP-5431). HEK293T cells used for lentiviral packaging were obtained from the Cell Bank of the Chinese Academy of Sciences (Shanghai, China; SCSP-502). MC38-OVA cells were generated from parental MC38 cells by lentiviral transduction and puromycin selection as described in the Methods. GFP-HOS cells were generated from parental HOS cells by lentiviral transduction with a GFP reporter vector, followed by antibiotic selection or fluorescence-based enrichment.
Authentication	All cell lines used in this study, including HOS, MC38 and HEK293T cells, were authenticated by Shanghai Jinyuan Biotechnology Co., Ltd. before use. Cell identity was further monitored during routine culture based on morphology and growth characteristics, and all cell lines were regularly tested to confirm the absence of mycoplasma contamination.
Mycoplasma contamination	Mycoplasma contamination: Cell lines were regularly tested and confirmed negative for mycoplasma contamination.
Commonly misidentified lines (See ICLAC register)	Commonly misidentified lines: None of the cell lines used in this study are known to be commonly misidentified (to the best of our knowledge.

Animals and other research organisms

Policy information about studies involving animals; ARRIVE guidelines recommended for reporting animal research, and Sex and Gender in Research

Laboratory animals	Mus musculus. Male C57BL/6J mice and male NSG mice aged 6–8 weeks were used in this study. C57BL/6J mice (Strain No. N000013) were purchased from GemPharmatech (Nanjing, China), and NSG mice (Strain No. NM-NSG-001) were obtained from Shanghai Model Organisms Center (Shanghai, China). Mice were housed under specific pathogen-free conditions at 22–24 °C with 55–60% humidity on a 12 h light and dark cycle, with ad libitum access to autoclaved food and water.
Wild animals	This study did not involve wild animals.
Reporting on sex	Sex was considered at the study-design stage for vertebrate animal experiments. Male mice were used to control experimental variables and reduce biological variability in preclinical efficacy testing. Sex was not used as a grouping variable, and sex-based analyses were not performed because only male mice were included and the study was designed to evaluate therapeutic mechanism and antitumour efficacy rather than sex-specific effects.
Field-collected samples	This study did not involve field-collected samples.
Ethics oversight	All animal procedures were conducted in accordance with protocols approved by the Institutional Animal Care and Use Committee (IACUC) of Deruikang Biotechnology Co., Ltd. (Approval No. DRK20240601001). All surgical interventions were performed under anaesthesia via intraperitoneal injection of 1% pentobarbital sodium, and all efforts were made to minimize animal suffering. Animals were euthanized by an overdose of pentobarbital sodium, followed by confirmation of death.

Note that full information on the approval of the study protocol must also be provided in the manuscript.

Clinical data

Policy information about clinical studies

All manuscripts should comply with the ICMJE guidelines for publication of clinical research and a completed CONSORT checklist must be included with all submissions.

Clinical trial registration	Not applicable.
Study protocol	Not applicable.
Data collection	Not applicable.
Outcomes	Not applicable.

Plants

Seed stocks	Not applicable.
Novel plant genotypes	Not applicable.
Authentication	Not applicable.

Flow Cytometry

Plots

Confirm that:

- [ ] The axis labels state the marker and fluorochrome used (e.g. CD4-FITC).
- [ ] The axis scales are clearly visible. Include numbers along axes only for bottom left plot of group (a 'group' is an analysis of identical markers).
- [ ] All plots are contour plots with outliers or pseudocolor plots.
- [x] A numerical value for number of cells or percentage (with statistics) is provided.

Methodology

Sample preparation	Tumour tissues were harvested at the indicated time points and processed into single-cell suspensions for flow cytometry. Tissues were mechanically dissociated and enzymatically digested, followed by filtration through a cell strainer to remove debris. Red blood cells were lysed when required. Cells were washed, counted, and stained in staining buffer. For PBMC-derived human NK cell assays, PBMCs were obtained as de-identified biospecimens and processed according to standard protocols prior to antibody staining. For intracellular staining, cells were fixed and permeabilized using a commercial fixation and permeabilization kit following the manufacturer's instructions.
Instrument	Flow cytometry data were collected using a Beckman Coulter flow cytometer
Software	Data were acquired using the Beckman Coulter instrument acquisition software and analysed using FlowJo v10.8.1. Statistical analysis and graphing were performed using GraphPad Prism.
Cell population abundance	Human NK cells were purified from PBMCs by negative selection using the EasySep Human NK Cell Isolation Kit, and NK-cell purity was verified by flow cytometry as live $CD3^{-}CD56^{+}$ cells before downstream experiments. Mouse OT-I $CD8^{+}$ T cells were isolated from splenocytes using CD8a MicroBeads and MS columns placed in a magnetic separator; the labelled $CD8^{+}$ T cells were collected as the positive fraction, and purity was confirmed by flow cytometry, typically exceeding 90%. Bone marrow-derived macrophages were generated by differentiation culture and adherent-cell recovery rather than fluorescence-activated cell sorting.
Gating strategy	Flow-cytometry data were analysed using FlowJo. Cells were first gated by FSC and SSC to exclude debris, followed by live-cell gating using a fixable viability dye. Human NK cells were identified as live $CD3^{-}CD56^{+}$ cells, and mouse $CD8^{+}$ T cells were identified as live $CD3^{+}CD8^{+}$ cells where applicable. Effector readouts were quantified within the indicated live immune-cell populations, including CD107a, perforin, granzyme B and IFN-γ. Fluorescence compensation was performed using single-stain controls, and positive and negative staining boundaries were defined using fluorescence-minus-one controls where appropriate.

- [ ] Tick this box to confirm that a figure exemplifying the gating strategy is provided in the Supplementary Information.
