# Supplementary material for: Dual compartment lipid carriers hijack exocytosis to empower natural killer cells against solid tumours
Source: Nat Commun. 2026 Jul 21;17:8906. doi: 10.1038/s41467-026-75852-6 (PMC13503815; doi:10.1038/s41467-026-75852-6)
Supplement: Supplementary file 3 [file 41467_2026_75852_MOESM3_ESM.pdf]

nature portfolio

Peer Review File

Dual compartment lipid carriers hijack exocytosis to empower natural killer cells against solid tumours

Corresponding Author: Professor Binghao Li

This manuscript has been previously reviewed at another journal. This document only contains information relating to versions considered at Nature Communications.

This file contains all reviewer reports in order by version, followed by all author rebuttals in order by version.

Version 0:

Reviewer comments:

Reviewer #2

(Remarks to the Author)
This study reports a nanomaterial-based strategy to overcome immune suppression induced tumor metabolism. LNPs were designed to deliver NMN (nicotinamide mononucleotide) to replenish the intracellular NAD+, showing transient improvement in the NK cell anti-tumor responses and ER-hybrid nanoparticles were generated by fusing ER membranes isolated from human NK cells with lipid nanoparticles and then incorporating DCA. This strategy of using ER-hybrid nanoparticles is not new as has been previously described in refences 29 and 30 though in the current study incorporation of metabolites and using in combination of NK cells is new. The major strength of this study is use of multiple in vivo models including PDX bearing mice and its broad application across NK, T and macrophages. However, I have following major comments for the the authors to address:

This study reports a nanomaterial-based strategy to overcome immune suppression induced tumor metabolism. LNPs were designed to deliver NMN (nicotinamide mononucleotide) to replenish the intracellular NAD+, showing transient improvement in the NK cell anti-tumor responses and ER-hybrid nanoparticles were generated by fusing ER membranes isolated from human NK cells with lipid nanoparticles and then incorporating DCA. This strategy of using ER-hybrid nanoparticles is not new as has been previously described in refences 29 and 30 though in the current study incorporation of metabolites and using in combination of NK cells is new. The major strength of this study is use of multiple in vivo models including PDX bearing mice and its broad application across NK, T and macrophages. However, I have following comments for the the authors to address:

1. NMN@LNP loaded NK cells were used to replenish NAD in NK cells – Why are the NK cells treated with the NPs prior to their exposure to tumor cells? Is there a benefit of prior nanoparticle loading of NK cells as opposed to adding the NPs to the NK+tumor cell co-culture?

2. The ER-guided strategy was successfully demonstrated in T cells and macrophages. The practical application of the strategy is still in question due to the high number of immune cells required.

Reviewer #3

(Remarks to the Author)
In this manuscript, the authors designed a dual-compartment system where lipid nanoparticles deliver nicotinamide mononucleotide (NMN) to rescue intracellular NAD^+ reserves while ER-hybrid lipid nanoparticles encapsulating dichloroacetate (DCA) hijack the secretory axis to achieve DCA release at the cell surface. They proved the platform could restores immunometabolic competence and re-establishes CAR-NK efficacy against refractory solid tumours. However, the proposed "dual-compartment nanocarrier + exocytosis modulation" strategy essentially represents a simple combination of two well-characterized molecules (NMN for NAD^+ replenishment and DCA for lactate inhibition) via previously reported delivery technologies. This design suffers from a lack of technical novelty and fails to present innovative advances in nanocarrier engineering or delivery mechanisms. Several concerns need to be addressed.
(1) Although the authors claim to have achieved stimulus-responsive DCA exocytosis, they provide only a vague definition

of stimulus, failing to clearly specify what exactly triggers the response. This ambiguity renders the claimed core advantage of "spatiotemporal precision" unsubstantiated. In addition, the term "spatiotemporal metabolic reprogramming" is used repeatedly but never defined.
(2) Whether the sustained, cytosolic release of NMN temporally with the stimulus-responsive exocytosis of DCA. How this temporal relationship is characterized and optimized to ensure that NAD^+ replenishment and lactate clearance support each other continuously during critical windows of immune cell activity, rather than presenting a mismatched or sequential action that could limit therapeutic efficacy?
(3) How much of the encapsulated DCA is actually secreted extracellularly via exocytosis, and what fraction remains intracellular or is lost due to passive diffusion or degradation? Is the extracellular DCA concentration resulting from exocytosis sufficient to inhibit lactate production in the local tumor microenvironment?
(4) While the authors show that DCA-loaded ER-hybrid liposomes do not impair normal secretory functions in NK cells, key data for the full dual-compartment system are currently lacking.
(5) In Figure S5c-f, the x-axis labels are missing.

Reviewer #4

(Remarks to the Author)
This manuscript describes a nanocarrier-based strategy to enhance the efficacy of adoptive cellular therapies in solid tumors by metabolically reprogramming engineered immune cells. The authors design a dual-compartment nanoparticle system that targets two intracellular locations. Cytosolic lipid nanoparticles deliver nicotinamide mononucleotide (NMN) to restore intracellular NAD^+ levels that are depleted in the lactate-rich tumor microenvironment. In parallel, endoplasmic reticulum–targeted nanocarriers exploit the ER–Golgi trafficking pathway to enable stimulus-responsive exocytosis of dichloroacetate (DCA), with the aim of reducing extracellular lactate locally. Together, this approach is proposed to counteract lactate-driven immunosuppression and improve persistence and cytotoxic activity of CAR-NK cells (also in patient-derived xenograft (PDX) models, Figure 6). The authors report enhanced tumor control in experimental models and further show that this strategy may be applicable to other adoptive cell therapies, including T cells and macrophages (Figure 7).

Major comments.
1. Could the authors provide more direct evidence linking NAD^+ restoration via NMN delivery to potential improvements in IFN-γ production in CAR-NK, T and/or macrophage cells?
2. Both NMN and DCA delivery appear to be required for the observed therapeutic effects, but it remains unclear whether one component dominates the overall outcome. A clearer comparison of NMN alone, DCA alone, and the combined NMN+DCA treatment, focusing on functional readouts such as lactate production, IFN-γ production, and tumor control, would strengthen the conclusions.

Minor:
1. The manuscript would benefit from a clearer description of the different experimental groups in both the text and figures.
2.The @ usage is not wrong, but field-dependent. For Nature Comm is more appropriate to use e.g. NMN-LNPs or NMN-loaded LNPs instead of NMN@LNPs.

Version 1:

Reviewer comments:

Reviewer #2

(Remarks to the Author)
The authors have done a nice job in addressing my comments/questions.

Reviewer #3

(Remarks to the Author)
The authors addressed well all my concerns and made new experiments to support their claims. Now I can recommend this manuscript for publication in the present form.

Reviewer #4

(Remarks to the Author)
The authors have addressed the reviewers' comments thoroughly, and the revised manuscript has improved substantially. The newly included data are of high quality and strengthen the conclusions of the study. In particular, the additional experiments presented in Figs. S3, S5, and S18 are valuable and could further enhance the manuscript if at least some of these data were incorporated into the main figures rather than remaining solely in the supplementary material.

Open Access This Peer Review File is licensed under a Creative Commons Attribution 4.0 International License, which permits use, sharing, adaptation, distribution and reproduction in any medium or format, as long as you give appropriate credit to the original author(s) and the source, provide a link to the Creative Commons license, and indicate if changes were made.
In cases where reviewers are anonymous, credit should be given to 'Anonymous Referee' and the source.
The images or other third party material in this Peer Review File are included in the article's Creative Commons license, unless indicated otherwise in a credit line to the material. If material is not included in the article's Creative Commons license and your intended use is not permitted by statutory regulation or exceeds the permitted use, you will need to obtain permission directly from the copyright holder.
To view a copy of this license, visit https://creativecommons.org/licenses/by/4.0/

Reviewer #2 (original, NK cells, immunotherapy):

This study reports a nanomaterial-based strategy to overcome immune suppression induced tumor metabolism. LNPs were designed to deliver NMN (nicotinamide mononucleotide) to replenish the intracellular NAD+, showing transient improvement in the NK cell anti-tumor responses and ER-hybrid nanoparticles were generated by fusing ER membranes isolated from human NK cells with lipid nanoparticles and then incorporating DCA. This strategy of using ER-hybrid nanoparticles is not new as has been previously described in refences 29 and 30 though in the current study incorporation of metabolites and using in combination of NK cells is new. The major strength of this study is use of multiple in vivo models including PDX bearing mice and its broad application across NK, T and macrophages. However, I have following major comments for the the authors to address:

1. NMN@LNP loaded NK cells were used to replenish NAD in NK cells – Why are the NK cells treated with the NPs prior to their exposure to tumor cells? Is there a benefit of prior nanoparticle loading of NK cells as opposed to adding the NPs to the NK+tumor cell co-culture?

Response:

We sincerely thank the reviewer for raising this important and practical question. We agree that the rationale for preloading NK cells with NMN-LNPs before tumour exposure should be clarified more explicitly. To address this point, we performed a direct comparison between prior intracellular NMN-LNP loading and direct NMN-LNP addition during NK–tumour co-culture. These data show that prior loading provides a measurable early functional advantage over direct addition and allows cleaner assessment of NK-cell-intrinsic NAD^+ replenishment.

The NMN-LNP preloading procedure was designed to model an *ex vivo* engineered adoptive NK-cell therapy workflow, in which NK cells are metabolically conditioned ex vivo, unbound nanoparticles are removed through washing, and the resulting engineered NK-cell product is subsequently evaluated in tumour co-culture systems or administered *in vivo*. Therefore, prior NMN-LNP loading is not simply a convenient in vitro treatment sequence, but part of the intended therapeutic format. We compared three conditions: G1, NK cells + HOS-GFP tumour cells; G2, NK cells + HOS-GFP tumour cells with NMN-LNPs added at the onset of co-culture; and G3, NMN-LNP-preloaded and washed NK cells + HOS-GFP tumour cells **(Fig. R1a)**.

This comparison showed that prior NMN-LNP loading provided an early functional advantage. At

4 h, NMN-LNP-preloaded NK cells showed significantly higher CD107a expression than both untreated NK cells and NK cells receiving NMN-LNPs only at the start of co-culture, whereas direct NMN-LNP addition did not significantly improve CD107a expression compared with untreated NK cells **(Fig. R1b,c)**. A similar pattern was observed for perforin expression, supporting a stronger early recovery of cytotoxic effector function in the preloaded group **(Fig. R1d,e)**. By 12–24 h, the direct-addition group showed partial recovery of CD107a and perforin, which is consistent with the time required for nanoparticle uptake and intracellular processing (Fig. R1b–e). Functionally, residual HOS-GFP fluorescence at 24 h was lowest in the preloaded group, indicating stronger tumour-cell elimination by NMN-LNP-preloaded NK cells than by either untreated NK cells or NK cells receiving NMN-LNPs only during co-cultur**e (Fig. R1f,g)**.

The reviewer's question helped us clarify another important reason for using the preloading-and-wash design. If NMN-LNPs are added directly into the NK–tumour co-culture, free nanoparticles may be taken up by both NK cells and tumour cells through cellular uptake pathways. This would make the result harder to interpret, because tumour-cell NAD^+ metabolism can influence tumour survival, metabolic adaptation and immune-evasive phenotypes such as IFNγ-induced PD-L1 expression[1,2]. Therefore, functional changes observed after direct NMN-LNP addition could reflect both NK-cell NAD^+ replenishment and direct metabolic modulation of tumour cells, rather than NK-cell-intrinsic rescue alone. Taken together, our data support the use of the preloading-and-wash strategy. This approach better reflects the intended ex vivo adoptive NK-cell therapy workflow, provides NK cells with metabolic support before the earliest phase of tumour encounter, and reduces the confounding effect caused by free nanoparticles in a mixed NK–tumour system. Thus, we believe that prior NMN-LNP loading is more consistent with the intended *ex vivo* adoptive NK-cell therapy workflow.

Response Figure 1

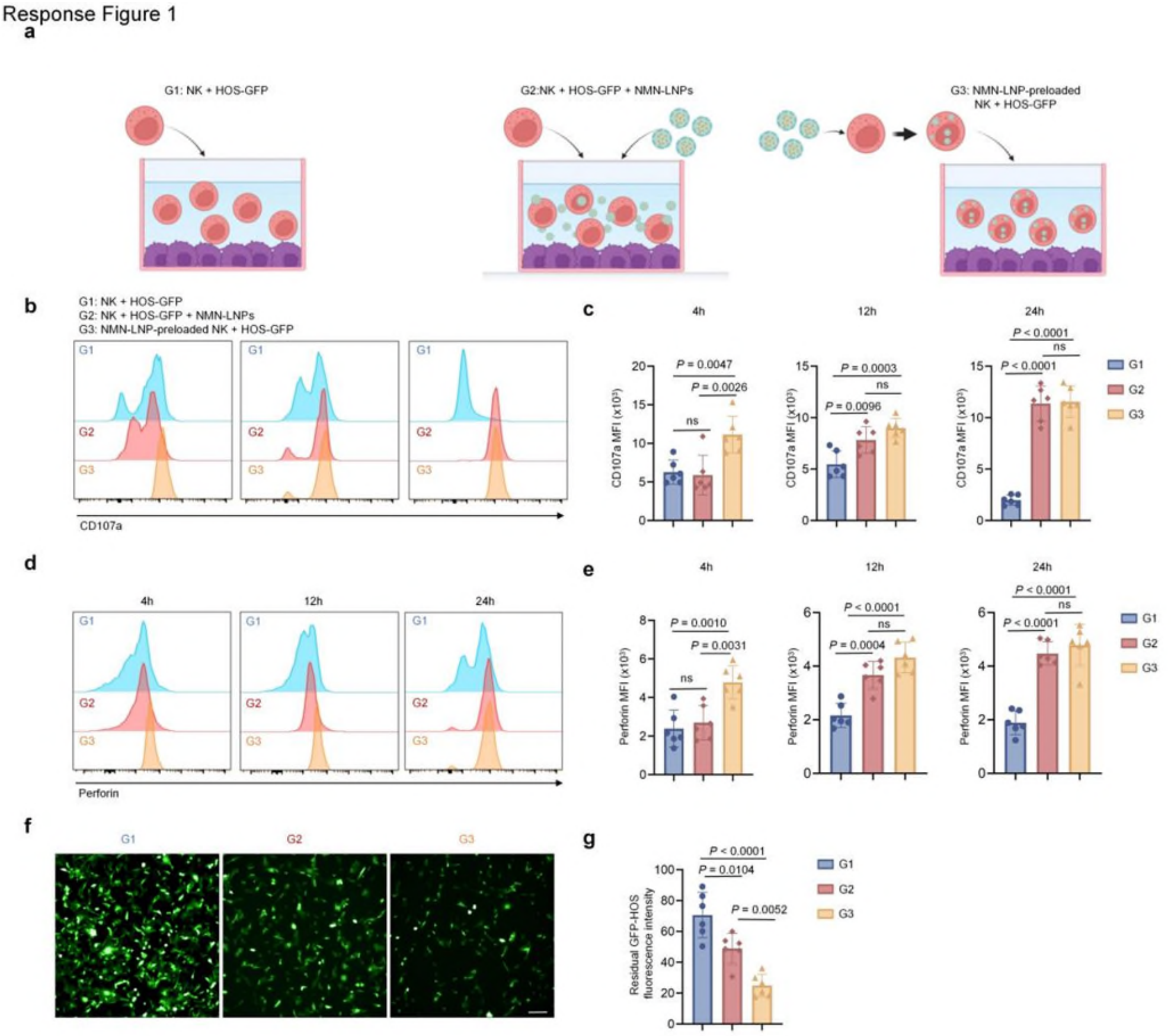

Fig. R1. Prior NMN-LNP preloading provides an early functional advantage over direct NMN-LNP addition during NK–tumour co-culture.
(a) Schematic of the three experimental conditions used to compare prior NMN-LNP loading with direct NMN-LNP addition during NK–tumour co-culture. G1, NK cells co-cultured with HOS-GFP tumour cells; G2, NK cells co-cultured with HOS-GFP tumour cells with NMN-LNPs added at the onset of co-culture; G3, NMN-LNP-preloaded and washed NK cells co-cultured with HOS-GFP tumour cells. (b) Representative flow-cytometry histograms showing CD107a expression in NK cells at 4, 12 and 24 h after co-culture under the indicated conditions. (c) Quantification of CD107a mean fluorescence intensity (MFI) at 4, 12 and 24 h. (d) Representative flow-cytometry histograms showing perforin expression in NK cells at 4, 12 and 24 h after co-culture under the indicated conditions. (e) Quantification of perforin MFI at 4, 12 and 24 h. (f) Representative fluorescence images of residual HOS-GFP tumour cells after 24 h of co-culture with the indicated NK-cell formulations. Reduced green fluorescence indicates enhanced tumour-cell killing. Scale bar, 100 µm. (g) Quantification of residual HOS-GFP fluorescence intensity at 24 h. Data are presented as mean ± SD. Statistical significance was determined by one-way ANOVA with multiple-comparison correction at each time point; ns, not significant.

2. The ER-guided strategy was successfully demonstrated in T cells and macrophages. The practical application of the strategy is still in question due to the high number of immune cells required.

Response:

We sincerely thank the reviewer for raising this important practical concern. We agree that the requirement for large numbers of immune cells is a key translational issue for adoptive cell therapy. In this study, ER-Guided Exo-T and ER-Guided Exo-M were used to test whether the ER-guided exocytic delivery principle could be extended beyond NK cells. The key practical question is whether the ER-guided strategy requires cell numbers beyond the range that is already clinically manufacturable for current ACT platforms.

Existing ACT products and clinical-stage platforms have shown that therapeutic immune-cell doses can be generated under controlled manufacturing conditions. In pivotal CAR-T studies, tisagenlecleucel was administered at a median dose of 3.1×10^6 transduced viable T cells/kg, and axicabtagene ciloleucel at 2×10^6 CAR-T cells/kg. Clinical CAR-NK studies have also tested dose levels from 1×10^5 to 1×10^7 CAR-NK cells/kg, while the first-in-human CT-0508 CAR-macrophage trial supports the clinical manufacturability of macrophage-based ACT products. Collectively, these clinical precedents indicate that ACT-relevant cell numbers are technically achievable with current manufacturing platforms and can be produced under controlled quality and cost-management frameworks.

In addition, the ER-derived component is not expected to impose a substantial donor-cell burden. As described in the Methods, ER isolation from 1×10^8 donor cells yields approximately 200–300 μg ER membrane protein, which is sufficient to prepare EMLNPs for arming a much larger number of therapeutic cells under our current loading conditions. Based on our formulation ratio, the additional ER-donor cell input would account for only approximately 0.8–1.25% of the therapeutic-cell number, even under a conservative same-lineage sourcing assumption. This further supports the scalability and practical manufacturability of the ER-guided strategy.

Reviewer #3 (new, nanomedicine):

In this manuscript, the authors designed a dual-compartment system where lipid nanoparticles deliver nicotinamide mononucleotide (NMN) to rescue intracellular NAD^{+} reserves while ER-hybrid lipid nanoparticles encapsulating dichloroacetate (DCA) hijack the secretory axis to achieve DCA release at the cell surface. They proved the platform could restores immunometabolic competence and re-establishes CAR-NK efficacy against refractory solid tumours. However, the proposed "dual-compartment nanocarrier + exocytosis modulation" strategy essentially represents a simple combination of two well-characterized molecules (NMN for NAD^{+} replenishment and DCA for lactate inhibition) via previously reported delivery technologies. This design suffers from a lack of technical novelty and fails to present innovative advances in nanocarrier engineering or delivery mechanisms. Several concerns need to be addressed.

Response:

We sincerely thank the reviewer for raising this important concern regarding the conceptual and technical novelty of our platform. We agree that NMN and DCA are individually well-characterized metabolic modulators, and we also acknowledge that ER-derived membrane-assisted nanocarrier systems have been explored in previous studies. We have therefore revised the manuscript to clarify that the novelty of this work does not lie in the chemical identity of NMN or DCA, nor in simply combining two known metabolic agents. Rather, the central advance of our study is the conversion of a conventional inward nanoparticle-delivery concept into an outward, exocytosis-oriented delivery mechanism operated by living immune cells. Conventional liposomes or LNPs are typically designed to enter target cells and release their payload intracellularly. In that setting, the nanocarrier functions mainly as an inward delivery vehicle, and the therapeutic action is expected to occur within the recipient cell after uptake and intracellular release.

Our ER-guided DCA-EMLNP module is conceptually different. Here, the nanocarrier is first loaded into NK cells ex vivo, and the ER-derived membrane is not used simply as a passive coating or uptake-enhancing shell. Instead, it provides an organelle-originated trafficking identity that biases the intracellular fate of the DCA-loaded nanocarrier toward the ER–Golgi secretory route. As a result, DCA is not intended to be released mainly inside the carrier NK cell; rather, the engineered NK cell functions as a living exocytotic delivery unit that transports and exports DCA toward the tumour-facing pericellular space. Therefore, the key technical distinction is a reversal of delivery

direction: from conventional endocytosis-dependent inward delivery into target cells to ER–Golgi trafficking-directed outward delivery from engineered immune cells. This exocytosis-oriented design enables DCA to act locally at the NK–tumour interface to attenuate lactate accumulation in the surrounding tumour microenvironment, while NMN-LNPs separately maintain NK-cell-intrinsic NAD^+/NADH balance. Thus, the platform is not a simple NMN+DCA co-formulation, but a dual-route nanocarrier system in which one module supports intracellular immune-cell metabolism and the other uses organelle-guided exocytic export to remodel the extracellular metabolic niche.
To strengthen and clarify this distinction, we performed several additional experiments and made corresponding revisions in the manuscript. First, we revised the terminology throughout the manuscript by replacing the potentially misleading phrases "stimulus-responsive DCA exocytosis" and "stimulus-triggered DCA release" with "ER–Golgi trafficking-directed exocytic DCA export". This change more accurately reflects the mechanism supported by our data and avoids overstatement of a classical physicochemical stimulus-responsive release process. We also added a conceptual model to define subcellular spatiotemporal delivery in our system, in which NK-cell tumour homing and ER-guided intracellular trafficking jointly localize DCA export to the tumour-facing pericellular space during the relevant post-infusion functional window (Fig. R2).

Second, we established an LC–MS/MS-based DCA mass-balance analysis to quantify the fate of encapsulated DCA after DCA-EMLNP loading. This analysis showed progressive extracellular DCA accumulation over time, reciprocal intracellular DCA loss, a limited unrecovered fraction, and much lower passive leakage from cell-free DCA-EMLNPs compared with the cell-loaded system. These data support cell-associated outward DCA export rather than simple passive diffusion or nonspecific leakage (Fig. 4h–j and Fig. S16). We further showed that DCA-EMLNP-loaded NK cells reduced intratumoural lactate over time, indicating that the exported DCA was functionally sufficient to suppress lactate accumulation in the local tumour microenvironment in the tested model (Fig. R3).

Third, to support the metabolic reprogramming component beyond endpoint lactate measurement, we added pathway-level evidence showing that ER-Guided Exo-NK treatment reduced PDHA1 phosphorylation in tumour tissues, as demonstrated by western blotting, p-PDHA1/PDHA1

quantification, p-PDHA1 immunohistochemistry and positive-area analysis (Fig. S17a–e). Because DCA inhibits PDK activity and thereby reduces inhibitory phosphorylation of PDHA1, these data support that the exported DCA acts on the tumour lactate-producing metabolic pathway.

Fourth, we added time-aligned analyses to examine whether intracellular NAD^+ support and extracellular DCA export occur within the same critical immune-cell activity window. These data showed that DCA export progressively increased during the post-homing functional period and was largely established by 72 h, before NMN-supported NK-cell function markedly declined under persistent lactate stress. This supports a coordinated temporal relationship between NMN-mediated NK-cell metabolic support and DCA-mediated lactate relief, rather than a mismatched or merely sequential action (Fig. S17f,g).

Fifth, we directly compared NK cells, NMN-LNPs-NK cells, DCA-EMLNPs-NK cells and NMN-LNPs-DCA-EMLNPs-NK cells at the same NK-cell number. The combined dual-compartment system produced the strongest tumour control, IFN-γ production, CD107a/perforin recovery and tumour IFN-γ positivity, whereas either NMN or DCA alone produced only partial effects. Tumour lactate analysis further showed that DCA-containing formulations were responsible for lactate reduction, while NMN-containing formulations supported NK-cell effector recovery. These data support that the system is not merely a pharmacological co-administration of two metabolic agents, but a compartmentally organized immune-cell engineering strategy in which the two modules operate in distinct spatial compartments and contribute non-redundantly to the therapeutic outcome. (Fig. S18).

Finally, we expanded the safety and functional evaluation of the complete dual-compartment system. We showed that NMN-LNPs-DCA-EMLNPs-loaded NK cells did not globally impair CD107a or perforin expression, and analogous dual-loading experiments in T cells and macrophages showed preserved granzyme B/perforin or TNF-α/IL-6 secretion, respectively. These data indicate that the full dual-compartment engineering process preserves native immune-cell secretory and effector functions while adding compartmentalized metabolic support (Fig. S5 and Fig. S8).

Taken together, these revisions and added experiments support that our platform is not a simple NMN+DCA co-treatment using previously reported delivery technologies. Rather, it represents a spatially organized immune-cell engineering strategy in which intracellular NAD^+ restoration and ER–Golgi trafficking-directed extracellular DCA export are functionally coupled to reinforce adoptive NK cells against lactate-driven tumour immunosuppression. We have revised the Introduction and Results sections, and clarified the conceptual framing in the revised manuscript.

(1) Although the authors claim to have achieved stimulus-responsive DCA exocytosis, they provide only a vague definition of stimulus, failing to clearly specify what exactly triggers the response. This ambiguity renders the claimed core advantage of "spatiotemporal precision" unsubstantiated. In addition, the term "spatiotemporal metabolic reprogramming" is used repeatedly but never defined.

Response:

We apologize for the ambiguity caused by our previous use of the term “stimulus-responsive”. We agree with the reviewer that this term was not appropriate in the classical nanomedicine sense. In conventional stimulus-responsive nanocarriers, payload release is triggered by defined physicochemical or tumour-microenvironmental cues, such as pH, ROS, enzymes, hypoxia, light or temperature. In our platform, DCA export is not controlled by such an external trigger. We have therefore removed “stimulus-responsive DCA exocytosis”, “stimulus-triggered DCA release” and related wording throughout the manuscript, and replaced them with “ER–Golgi trafficking-directed exocytic DCA export”.

We have also clarified the meaning of “spatiotemporal precision” in the revised manuscript. We do not use this term to imply classical stimulus-triggered nanoparticle disassembly or bulk tumour-site drug release. Rather, our system extends the concept of spatiotemporal precision from conventional tissue-level nanomedicine to subcellularly programmed delivery within a living immune cell. In this context, spatial precision refers to the ER–Golgi trafficking-directed relocation of DCA-EMLNPs from an intracellular compartment to the extracellular, tumour-facing pericellular space through exocytic export. Thus, DCA delivery is not simply intracellular release inside the carrier NK cell or nonspecific leakage into the bulk medium, but a directionally organized intracellular-to-extracellular transport process mediated by the secretory machinery of the engineered cell.

Temporal precision refers to the kinetic coordination between this cellular trafficking/exocytosis process and the therapeutic window of NK-cell activity. Because DCA export is governed by ER–Golgi trafficking and exocytic output, its extracellular availability develops over the post-loading/post-infusion period and overlaps with the time window in which engineered NK cells encounter tumour cells and require relief from lactate-mediated suppression. Therefore, in our study, "spatiotemporal precision" refers to subcellular spatial control of DCA movement across intracellular and extracellular compartments, together with temporal matching between exocytosis-mediated DCA output and NK-cell functional activity. This subcellular spatiotemporal delivery concept is illustrated in Fig. R2.

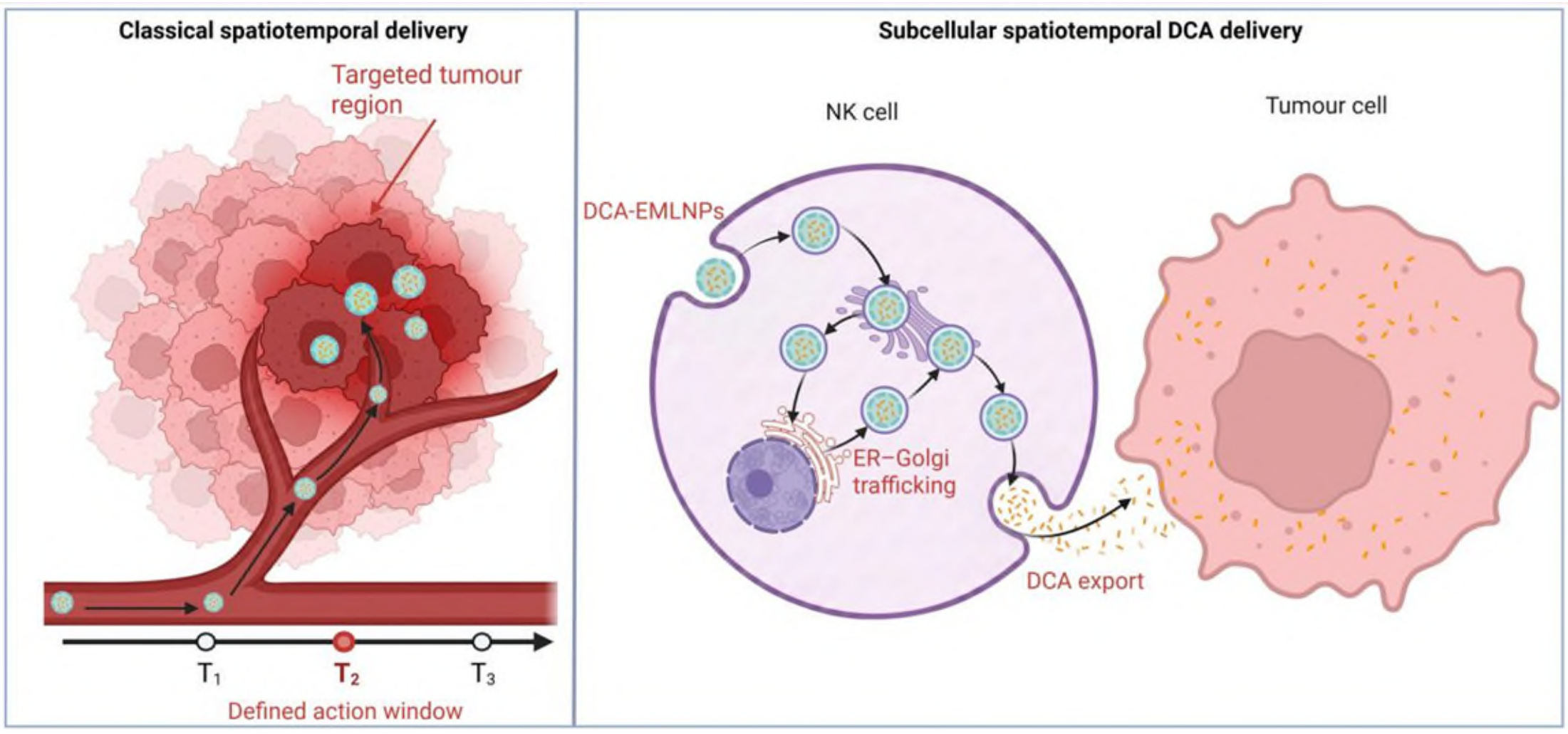

Fig. R2. Conceptual distinction between classical spatiotemporal delivery and subcellular spatiotemporal DCA delivery. Left, classical spatiotemporal delivery confines drug action to a targeted tumour region and a defined therapeutic time window, thereby limiting broad and continuous systemic exposure. Right, in our ER-guided strategy, spatiotemporal DCA delivery is achieved at the cellular and subcellular levels. DCA-EMLNPs are routed within engineered NK cells through ER–Golgi trafficking and subsequently exported toward the tumour-facing pericellular space by exocytosis. This subcellular delivery route aligns local DCA export with NK–tumour interaction, supporting tumour-localized lactate suppression during the relevant post-infusion functional window.

Finally, regarding the term "spatiotemporal metabolic reprogramming", we thank the reviewer for pointing out that this term was not clearly defined. Following the clarification of subcellular spatiotemporal delivery above, we have revised the manuscript to define this term as the metabolic consequence of ER–Golgi trafficking-directed DCA export from engineered NK cells. Specifically, "metabolic reprogramming" in our system refers to local remodelling of tumour lactate metabolism through the DCA-regulated PDK–PDH axis, rather than a broad or undefined metabolic effect. To

support this definition, we added pathway-level data to examine whether ER-Guided Exo-NK functionally affected the DCA-related tumour metabolic pathway, beyond endpoint lactate measurement. ER-Guided Exo-NK reduced tumour PDHA1 phosphorylation, as demonstrated by western blotting and p-PDHA1/PDHA1 quantification (Fig. S17a–c), and this was further confirmed by p-PDHA1 immunohistochemistry and positive-area analysis (Fig. S17d,e). Because DCA inhibits PDK activity and thereby reduces inhibitory phosphorylation of PDHA1, these data support that exported DCA acts on the tumour PDK–PDHA1 axis and attenuates lactate-associated tumour metabolic pressure. Taken together, we now use "spatiotemporal metabolic reprogramming" to describe the coupling of subcellularly controlled DCA export with local suppression of the tumour PDK–PDHA1–lactate metabolic axis. These revisions have been incorporated into the Introduction and Results sections, with corresponding figure legend and methodological updates (lines 28, 65–68, 74-75, 81-85, 305–308, 432, 766, 878-900, 1407-1417), and all corresponding changes are highlighted in colour in the revised manuscript.

Figure S17

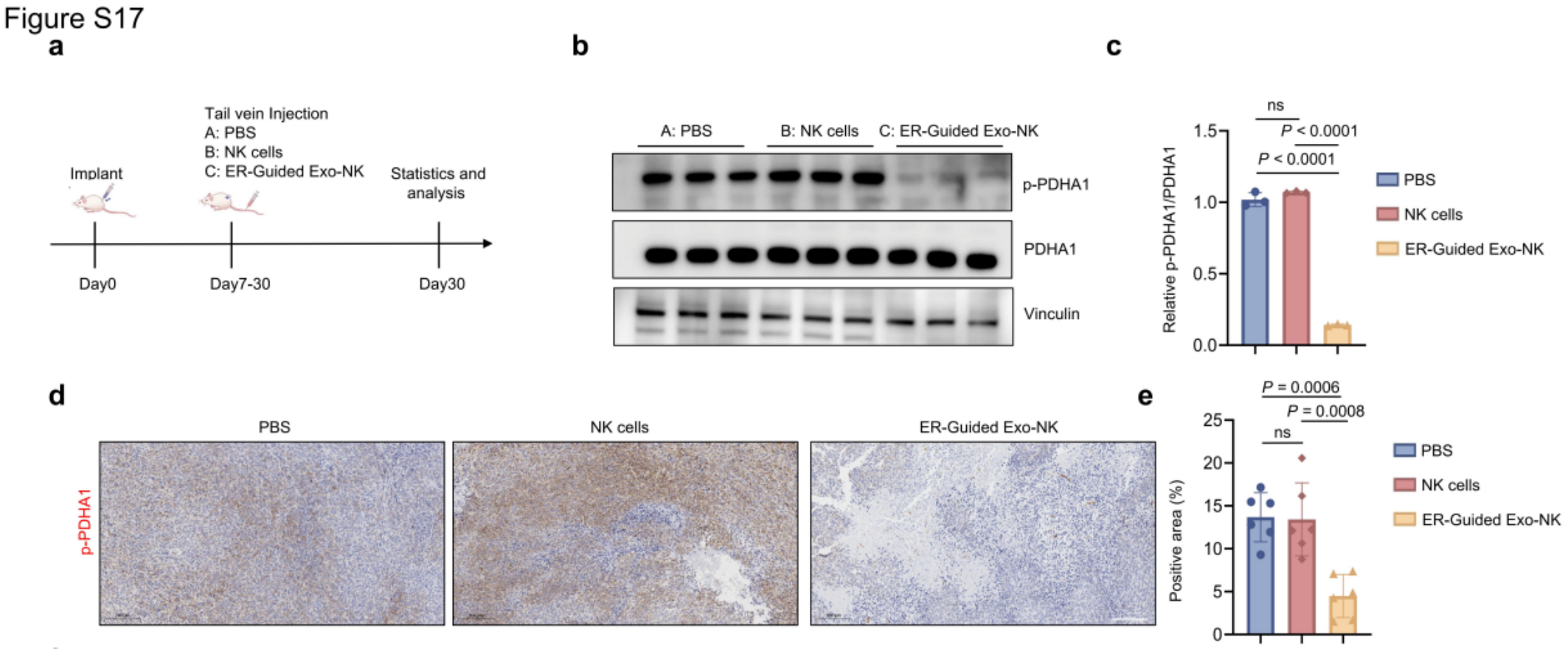

Figure S17. ER-Guided Exo-NK suppresses PDHA1 phosphorylation in tumour tissues.
(a) Schematic workflow for in vivo assessment of PDHA1 phosphorylation after treatment with PBS, NK cells or ER-Guided Exo-NK cells in HOS tumour-bearing mice. (b) Western blot analysis of p-PDHA1 and total PDHA1 in tumour tissues from the indicated treatment groups. Vinculin was used as the loading control. (c) Quantification of the relative p-PDHA1/PDHA1 ratio in panel b (mean ± SD). Statistics: one-way ANOVA with multiple comparisons; ns, not significant. (d) Representative immunohistochemical staining of p-PDHA1 in tumour sections from PBS-, NK-cell- and ER-Guided Exo-NK-treated mice. Scale bar, 200 µm. (e) Quantification of p-PDHA1-positive area in tumour sections shown in panel d (mean ± SD). Statistics: one-way ANOVA with multiple

comparisons; ns, not significant.

(2) Whether the sustained, cytosolic release of NMN temporally with the stimulus-responsive exocytosis of DCA. How this temporal relationship is characterized and optimized to ensure that NAD^+ replenishment and lactate clearance support each other continuously during critical windows of immune cell activity, rather than presenting a mismatched or sequential action that could limit therapeutic efficacy?

Response:

We thank the reviewer for raising this important point. We agree that the therapeutic value of the dual-compartment system depends on whether NAD^+ replenishment and lactate clearance occur within the critical window of NK-cell activity. We therefore characterized the timing of both modules in relation to NK-cell tumour homing and post-homing effector function. Based on previous imaging studies showing that adoptively transferred NK cells accumulate in tumours within approximately 24 h (PMID: 26272812), together with our own in vivo imaging showing detectable tumour-region accumulation of ER-Guided Exo-NK cells from 24 h and progressive accumulation over 24–96 h (Fig. 4m,n), we defined 24–96 h as the main post-homing functional window.

Within this window, the NMN and DCA modules are carried together in the same engineered NK-cell product but act in complementary compartments. NMN-LNPs maintain a higher intracellular NAD^+/NADH state and help preserve NK-cell cytotoxic activity under lactate stress, as supported by the NAD^+/NADH restoration and its association with CD107a degranulation **(Fig. S2d,g)**. In parallel, DCA-EMLNPs underwent ER–Golgi trafficking-directed exocytic export, with extracellular DCA progressively increasing during the same 24–96 h window (Fig. 4i,j). Thus, NMN provides early intracellular metabolic support as NK cells enter the tumour, while DCA export develops during the same post-homing period to relieve extracellular lactate pressure.

To make this relationship more explicit, we added a time-aligned coordination analysis (Fig. S17f,g). Under persistent lactate stress in the absence of effective lactate clearance, NMN-mediated support preserved NK-cell degranulation during the early post-homing period, but functional decline became evident by approximately 72 h. During this same 24–72 h overlap window, ER-guided DCA

export increased from 13.03 ± 1.30% at 24 h to 42.63 ± 2.08% at 48 h and 62.68 ± 2.45% at 72 h, reaching 65.20 ± 4.29% at 96 h (Fig. S17f). The DCA-export kinetics in Fig. S17f were derived from an independent experimental repeat performed for the time-alignment analysis, and showed the same progressive release pattern as the mass-balance experiment in Fig. 4i,j, with minor numerical variation attributable to independent cell preparations and nanoparticle batches. Thus, by 72 h, DCA export had reached approximately 96.1% of the 96 h level, indicating that lactate-relieving DCA output was largely established before NMN-supported NK-cell function markedly declined under continuous lactate stress. When DCA-mediated lactate relief was included in the ER-Guided Exo-NK system, NK-cell functional activity was maintained for a longer period and was accompanied by progressive tumour lactate reduction (Fig. S17g). These data support a temporally coordinated and functionally overlapping relationship between NAD^+ replenishment and lactate relief during the critical NK-cell activity window, rather than a mismatched or merely sequential action. These revisions have been incorporated into the Results section and Supplementary Fig. 17 legend (lines 269–286, 1417-1430), and all corresponding changes are highlighted in colour in the revised manuscript. We did not intend to claim that this timing represents a globally optimized regimen across all tumour models. Rather, we used the time-aligned analysis to verify that, under the current dosing and loading conditions, the two modules act within an overlapping functional window. We have revised the manuscript to avoid implying universal temporal optimization.

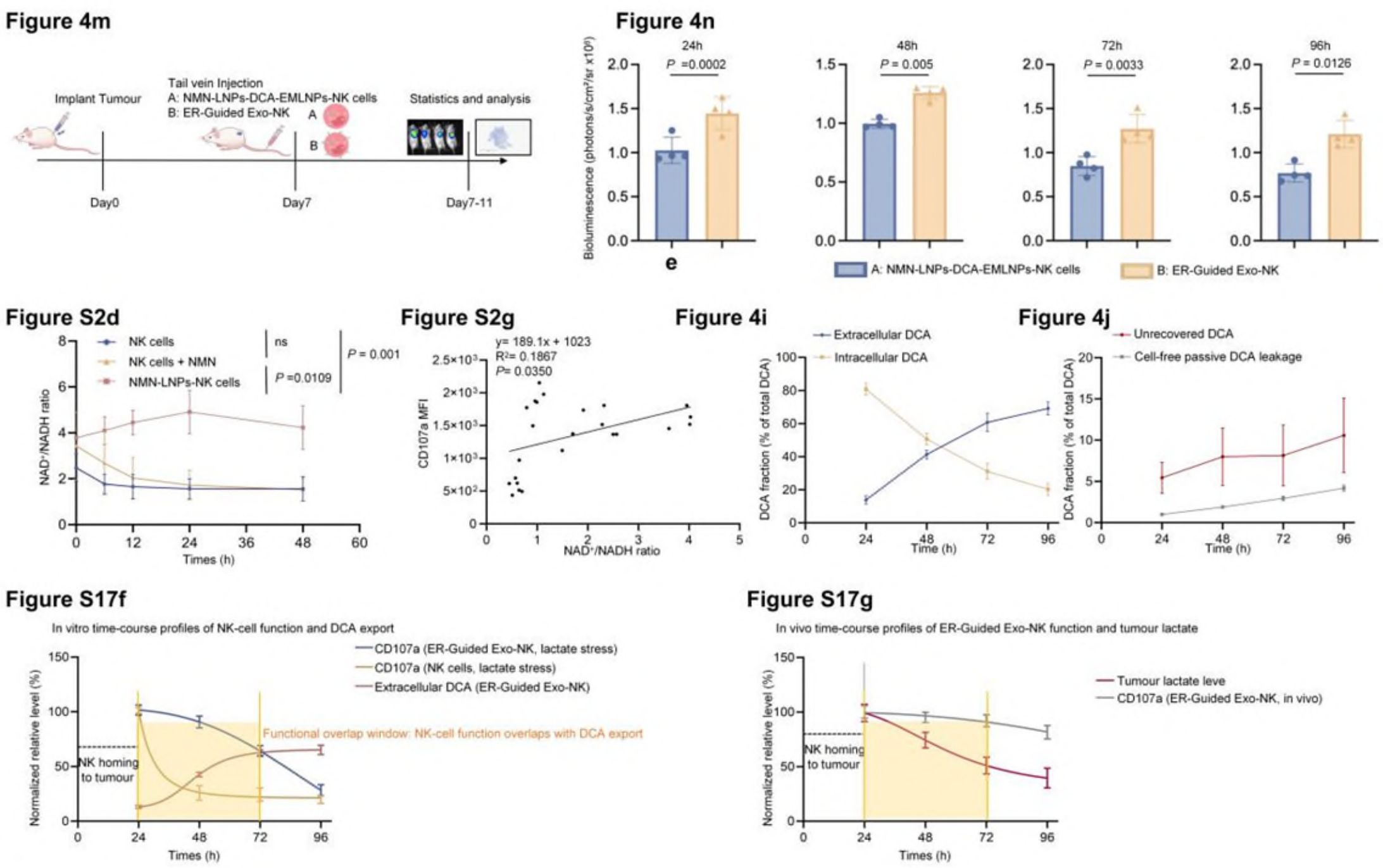

(Figure 4m) Schematic showing the in vivo tumour-homing assay. HOS tumour-bearing mice received tail-vein injection of either NMN-LNPs-DCA-EMLNPs-NK cells or ER-Guided Exo-NK cells on day 7 after tumour implantation, followed by longitudinal imaging and analysis from day 7 to day 11. (Figure 4n) Quantification of tumour-region fluorescence signals at 24, 48, 72 and 96 h after infusion. ER-Guided Exo-NK cells showed stronger and progressively increased tumour accumulation compared with non-targeted NMN-LNPs-DCA-EMLNPs-NK cells. (Figure S2d) Intracellular NAD^+/NADH ratio in NK cells after treatment with free NMN or NMN-LNPs under lactate-stress and washout conditions. NMN-LNPs maintained a higher NAD^+/NADH ratio over time compared with untreated NK cells or free NMN treatment. (Figure S2g) Correlation analysis between intracellular NAD^+/NADH ratio and CD107a mean fluorescence intensity (MFI) in NK cells, supporting the link between redox restoration and NK-cell degranulation capacity. (Figure 4i) LC–MS/MS-based quantification of extracellular and intracellular DCA fractions over time after cellular loading. Extracellular DCA progressively increased from 24 to 96 h, whereas intracellular DCA declined reciprocally. (Figure 4j) Quantification of the calculated unrecovered DCA fraction and cell-free passive DCA leakage over 24–96 h. Passive leakage from cell-free DCA-EMLNPs remained low compared with extracellular DCA accumulation from cell-loaded systems. (Figure S17f) Time-course alignment of CD107a expression and extracellular DCA export under lactate-stress conditions. ER-Guided Exo-NK cells maintained higher CD107a expression during the early post-homing functional window, while extracellular DCA progressively increased. The shaded region indicates the overlap window in which NK-cell function is preserved together with DCA export. (Figure S17g) Time-course alignment of ER-Guided Exo-NK functional activity and tumour lactate levels after systemic infusion. During the post-homing window, ER-Guided Exo-NK function remained preserved while tumour lactate levels progressively declined, supporting temporal coordination between immune-cell activity and lactate suppression.

(3) How much of the encapsulated DCA is actually secreted extracellularly via exocytosis, and what fraction remains intracellular or is lost due to passive diffusion or degradation? Is the extracellular DCA concentration resulting from exocytosis sufficient to inhibit lactate production in the local tumor microenvironment?

Response:

We thank the reviewer for raising this important and technically specific question. We agree that quantifying the fate of encapsulated DCA is necessary to support the proposed ER-guided exocytic export mechanism and to evaluate whether the exported DCA is functionally sufficient. To address this point, we established an LC–MS/MS-based DCA mass-balance assay **(Fig. 4h)**. In this workflow, total loaded DCA was first quantified and used as the denominator. After DCA-EMLNP loading into NK cells, intracellular DCA was measured from NK-cell lysates, extracellular DCA was measured from culture supernatants, and passive leakage was measured from cell-free DCA-EMLNPs incubated under the same conditions. Representative LC–MS/MS chromatograms were provided to validate DCA detection in each fraction, including total loaded DCA, cell-free passive leakage, extracellular DCA in culture supernatants, and intracellular DCA in NK-cell lysates **(Fig. S16a–d).**

Using this approach, extracellular DCA progressively increased from 13.86 ± 2.42% at 24 h to 41.31 ± 2.51% at 48 h, 60.77 ± 5.53% at 72 h and 69.13 ± 3.92% at 96 h, whereas intracellular DCA declined from 80.70 ± 3.63% to 50.70 ± 3.56%, 31.09 ± 4.67% and 20.29 ± 3.41% over the same time points **(Fig. 4i)**. The calculated unrecovered fraction remained limited, measuring 5.43 ± 1.87%, 7.99 ± 3.48%, 8.14 ± 3.69% and 10.58 ± 4.52% at 24, 48, 72 and 96 h, respectively **(Fig. 4j)**. In contrast, passive leakage from cell-free DCA-EMLNPs was much lower, increasing only from 0.99 ± 0.12% at 24 h to 1.89 ± 0.18%, 2.94 ± 0.27% and 4.18 ± 0.38% at 48, 72 and 96 h, respectively **(Fig. 4j)**. These data indicate that extracellular DCA detected in the cell-loaded system greatly exceeded cell-free passive leakage and was accompanied by reciprocal intracellular DCA loss, supporting cell-associated outward export rather than simple passive diffusion.

We further evaluated whether the exported DCA was sufficient to attenuate lactate accumulation in the local tumour microenvironment. Because lactate was measured directly in tumour tissues rather than in bulk culture medium or circulation, this assay reflects local tumour metabolic modulation. DCA-EMLNP-loaded NK cells progressively reduced intratumoural lactate over the same 24–96 h period compared with control groups, indicating that cell-associated DCA export was sufficient to suppress lactate accumulation in the tested tumour model **(Fig. R3a,b)**. We have revised the text to state this conclusion more precisely and to limit it to the current experimental setting, while noting that the modular formulation may be further adjusted in future models with higher lactate-producing metabolic burden. These revisions have been incorporated into the Results section ,methods section,

Fig.4 legend and Supplementary Fig.16 legend (lines 251–263, 852-877, 1142-1152, 1400-1406), and all corresponding changes are highlighted in colour in the revised manuscript.

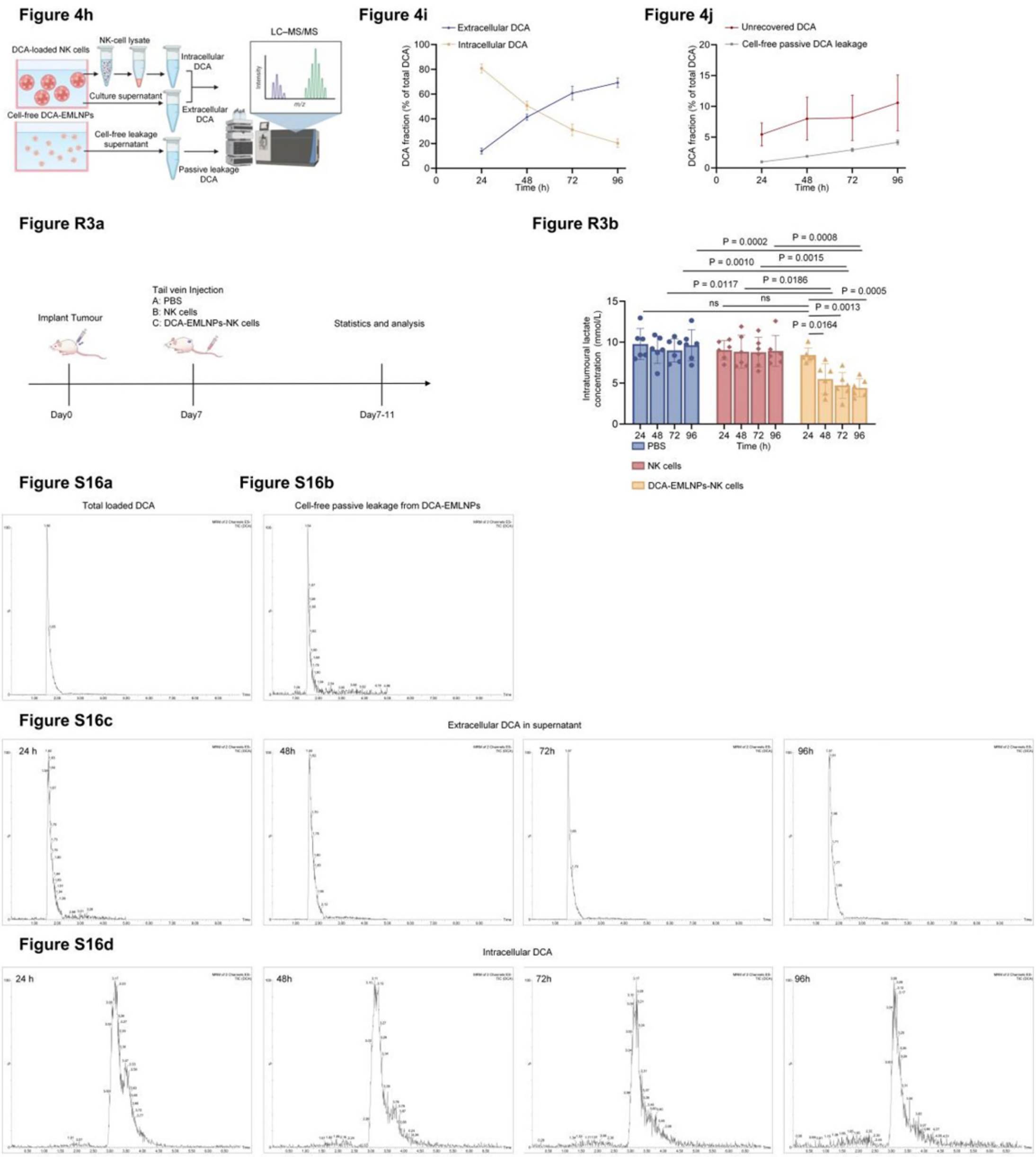

(Figure 4h) Schematic showing the LC–MS/MS-based DCA fate analysis after DCA-EMLNP loading. Total loaded DCA was first quantified as the denominator. After DCA-EMLNP loading into NK cells, intracellular DCA was measured from NK-cell lysates, extracellular DCA was measured from culture supernatants, and passive leakage was measured from cell-free DCA-EMLNPs incubated under identical conditions. (Figure 4i) LC–MS/MS-based quantification of extracellular and intracellular DCA fractions over time after DCA-EMLNP loading. Extracellular DCA progressively increased from 24 to 96 h, whereas intracellular DCA declined reciprocally, indicating time-dependent outward DCA export from loaded NK cells. (Figure 4j) Quantification of the calculated unrecovered DCA fraction and cell-free passive DCA leakage over 24–96 h. The unrecovered DCA fraction was calculated as 100% − extracellular DCA fraction − intracellular DCA

fraction. Passive leakage from cell-free DCA-EMLNPs remained low compared with extracellular DCA accumulation in the cell-loaded system. (Figure R3a) Schematic showing the in vivo HOS tumour model used to evaluate whether exported DCA is sufficient to suppress local tumour lactate accumulation. HOS tumour-bearing mice received tail-vein injection of PBS, native NK cells or DCA-EMLNP-loaded NK cells on day 7 after tumour implantation. Tumour samples were collected at 24, 48, 72 and 96 h after treatment for intratumoural lactate analysis. (Figure R3b) Quantification of intratumoural lactate concentrations at 24, 48, 72 and 96 h after treatment with PBS, native NK cells or DCA-EMLNP-loaded NK cells. DCA-EMLNP-loaded NK cells progressively reduced intratumoural lactate over time compared with PBS and native NK-cell controls, supporting that exported DCA is sufficient to suppress lactate accumulation in the local tumour microenvironment. (Figure S16a) Representative LC–MS/MS chromatogram of total loaded DCA after complete extraction from DCA-EMLNPs. This signal was used as the reference denominator for calculating extracellular, intracellular and unrecovered DCA fractions. (Figure S16b) Representative LC–MS/MS chromatogram of DCA detected in the supernatant of cell-free DCA-EMLNPs under passive leakage conditions. (Figure S16c) Representative LC–MS/MS chromatograms of extracellular DCA detected in culture supernatants collected from DCA-EMLNP-loaded NK cells at 24, 48, 72 and 96 h. (Figure S16d) Representative LC–MS/MS chromatograms of intracellular DCA detected in NK-cell lysates collected from DCA-EMLNP-loaded NK cells at 24, 48, 72 and 96 h.

(4) While the authors show that DCA-loaded ER-hybrid liposomes do not impair normal secretory functions in NK cells, key data for the full dual-compartment system are currently lacking.

Response:

We thank the reviewer for pointing out this important omission. We agree that the full dual-compartment system containing both NMN-LNPs and DCA-EMLNPs should be directly evaluated, rather than assessing DCA-EMLNPs alone. We therefore replaced and expanded the relevant supplementary data to include the complete NMN-LNPs-DCA-EMLNPs dual-loading system.

For NK cells, we compared NK cells, NMN-LNPs-NK cells, DCA-EMLNPs-NK cells and NMN-LNPs-DCA-EMLNPs-NK cells. The dual-loaded NK cells showed no significant change in CD107a or perforin expression compared with untreated or single-module controls, indicating preserved degranulation and cytotoxic granule function **(Fig. S5c–f)**. We further performed analogous tests in T cells and macrophages. NMN-LNPs-DCA-EMLNPs-T cells showed no significant changes in granzyme B or perforin levels **(Fig. S8i–l)**, and NMN-LNPs-DCA-EMLNPs-macrophages showed no significant changes in TNF-α or IL-6 secretion **(Fig. S8m,n)**.

Together, these data demonstrate that the complete dual-compartment system does not globally disrupt native secretory or effector functions in NK cells, T cells or macrophages. These revisions have been incorporated into the Results section and updated supplementary figure legends (lines 234–241, 392–396, 418–422, 1298-1305, 1343-1353), and all corresponding changes are highlighted in colour in the revised manuscript.

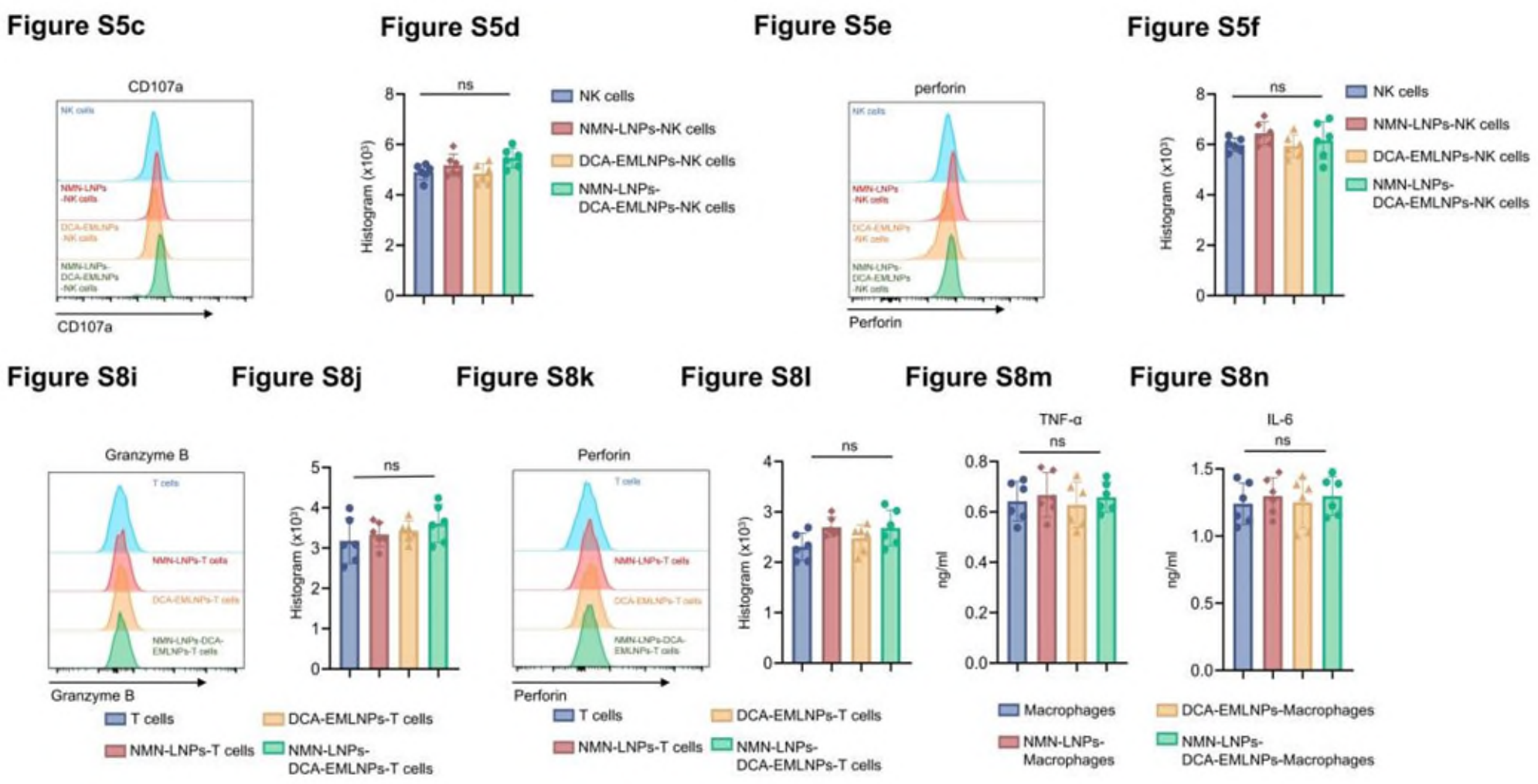

(Figure S5c) Representative flow-cytometry histograms of CD107a expression in NK cells, NMN-LNPs-NK cells, DCA-EMLNPs-NK cells and NMN-LNPs-DCA-EMLNPs-NK cells. (Figure S5d) Quantification of CD107a expression in NK cells, NMN-LNPs-NK cells, DCA-EMLNPs-NK cells and NMN-LNPs-DCA-EMLNPs-NK cells. No significant difference was observed among the indicated groups. (Figure S5e) Representative flow-cytometry histograms of perforin expression in NK cells, NMN-LNPs-NK cells, DCA-EMLNPs-NK cells and NMN-LNPs-DCA-EMLNPs-NK cells. (Figure S5f) Quantification of perforin expression in NK cells, NMN-LNPs-NK cells, DCA-EMLNPs-NK cells and NMN-LNPs-DCA-EMLNPs-NK cells. No significant difference was observed among the indicated groups. (Figure S8i) Representative flow-cytometry histograms of granzyme B expression in T cells, NMN-LNPs-T cells, DCA-EMLNPs-T cells and NMN-LNPs-DCA-EMLNPs-T cells. (Figure S8j) Quantification of granzyme B expression in T cells, NMN-LNPs-T cells, DCA-EMLNPs-T cells and NMN-LNPs-DCA-EMLNPs-T cells. No significant difference was observed among the indicated groups. (Figure S8k) Representative flow-cytometry histograms of perforin expression in T cells, NMN-LNPs-T cells, DCA-EMLNPs-T cells and NMN-LNPs-DCA-EMLNPs-T cells. (Figure S8l) Quantification of perforin expression in T cells, NMN-LNPs-T cells, DCA-EMLNPs-T cells and NMN-LNPs-DCA-EMLNPs-T cells. No significant difference was observed among the indicated groups. (Figure S8m) Quantification of TNF-α secretion from macrophages, NMN-LNPs-macrophages, DCA-EMLNPs-macrophages and NMN-LNPs-DCA-EMLNPs-macrophages. No significant difference was observed among the indicated groups. (Figure S8n) Quantification of IL-6 secretion from macrophages, NMN-LNPs-macrophages, DCA-EMLNPs-macrophages and NMN-LNPs-DCA-EMLNPs-macrophages. No significant difference was observed among the indicated groups.

(5) In Figure S5c-f, the x-axis labels are missing.

Response:

We thank the reviewer for pointing this out and apologize for the omission. We have now added the missing x-axis labels to Fig. S5c–f. The revised panels clearly indicate the four groups: NK cells, NMN-LNPs-NK cells, DCA-EMLNPs-NK cells, and NMN-LNPs-DCA-EMLNPs-NK cells. The figure legend has also been updated accordingly.

Figure S5c

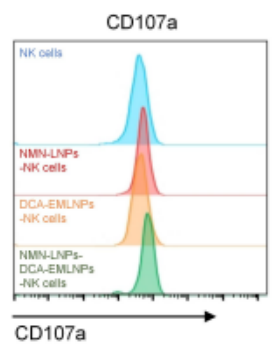

Figure S5d

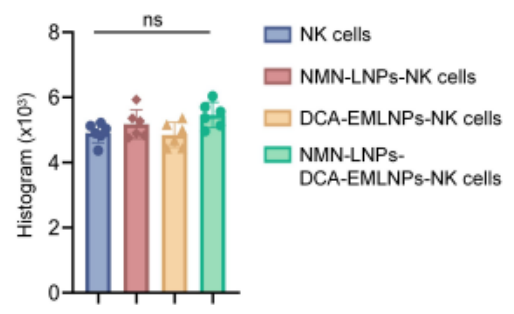

Figure S5e

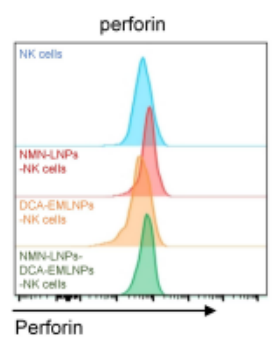

Figure S5f

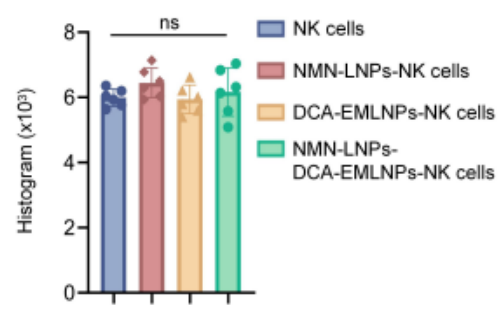

(Figure S5c–f) Representative flow-cytometry histograms and quantification of CD107a and perforin expression in NK cells, NMN-LNPs-NK cells, DCA-EMLNPs-NK cells and NMN-LNPs-DCA-EMLNPs-NK cells. No significant differences were observed among the indicated groups. (Figure S8i) Representative flow-cytometry histograms of granzyme B expression in T cells, NMN-LNPs-T cells, DCA-EMLNPs-T cells and NMN-LNPs-DCA-EMLNPs-T cells.

Reviewer #4 (new, replacing R1, cancer metabolic reprogramming):

This manuscript describes a nanocarrier-based strategy to enhance the efficacy of adoptive cellular therapies in solid tumors by metabolically reprogramming engineered immune cells. The authors design a dual-compartment nanoparticle system that targets two intracellular locations. Cytosolic lipid nanoparticles deliver nicotinamide mononucleotide (NMN) to restore intracellular NAD^+ levels that are depleted in the lactate-rich tumor microenvironment. In parallel, endoplasmic reticulum–targeted nanocarriers exploit the ER–Golgi trafficking pathway to enable stimulus-responsive exocytosis of dichloroacetate (DCA), with the aim of reducing extracellular lactate locally. Together, this approach is proposed to counteract lactate-driven immunosuppression and improve persistence and cytotoxic activity of CAR-NK cells (also in patient-derived xenograft (PDX) models, Figure 6). The authors report enhanced tumor control in experimental models and further show that this strategy may be applicable to other adoptive cell therapies, including T cells and macrophages (Figure 7).

Major comments.

1. Could the authors provide more direct evidence linking NAD^+ restoration via NMN delivery to potential improvements in IFN-γ production in CAR-NK, T and/or macrophage cells?

Response:

We sincerely thank the reviewer for this important and constructive suggestion. To directly validate whether NMN-LNP reverses lactate-induced immune suppression through NAD^+ replenishment,

we performed comprehensive functional analyses in three key anti-tumor immune cell types (NK cells, $CD8^+$ T cells, and macrophages) and examined the correlation between intracellular NAD^+/NADH ratio and cytokine production capacity. Specifically, we examined IFN-γ production in lactate-stressed NK cells and $CD8^+$ T cells, the two major IFN-γ-producing effector-cell populations in our platform. For macrophages, we followed the same logic of linking NAD^+ restoration to cytokine-producing function, and selected TNF-α as a lineage-appropriate functional readout because it directly reflects macrophage-associated inflammatory and antitumour activity[3,4] . These assays provide a more direct functional assessment by measuring cytokine output and its association with intracellular NAD^+/NADH restoration.

In NK cells, lactate markedly reduced IFN-γ secretion, whereas NMN-LNP treatment restored IFN-γ release under lactate stress **(Fig. S3c)**. Intracellular flow cytometry showed a consistent pattern, with lactate reducing the percentage of IFN-γ^+ NK cells and NMN-LNP treatment rescuing the IFN-γ^+ NK-cell fraction **(Fig. S3d,e)**. Importantly, IFN-γ^+ NK-cell frequency positively correlated with the intracellular NAD^+/NADH ratio **(Fig. S3f)**, supporting a direct functional association between NAD^+ restoration and IFN-γ-producing capacity.

In $CD8^+$ T cells, lactate similarly suppressed IFN-γ secretion and IFN-γ^+ T-cell frequency, whereas NMN-LNP treatment restored both extracellular IFN-γ release and intracellular IFN-γ positivity **(Fig. S15a–c)**. IFN-γ^+ T-cell frequency also positively correlated with the NAD^+/NADH ratio **(Fig. S15d)**.

For macrophages, lactate reduced TNF-α secretion, whereas NMN-LNP treatment restored TNF-α production under lactate stress **(Fig. S15e)**, and TNF-α levels positively correlated with intracellular NAD^+/NADH ratio **(Fig. S15f)**.

Together, these new data provide direct and robust evidence that NMN-LNP-mediated NAD^+ restoration is associated with improved IFN-γ production in NK and $CD8^+$ T cells, as well as enhanced macrophage-relevant anti-tumour cytokine output. These revisions have been incorporated into the Results section, Methods and Supplementary figure legend (lines 124–136, 382–388, 411–416,901-916, 1271-1277, 1388-1399), and all corresponding changes are highlighted in colour in the revised manuscript.

Figure S3

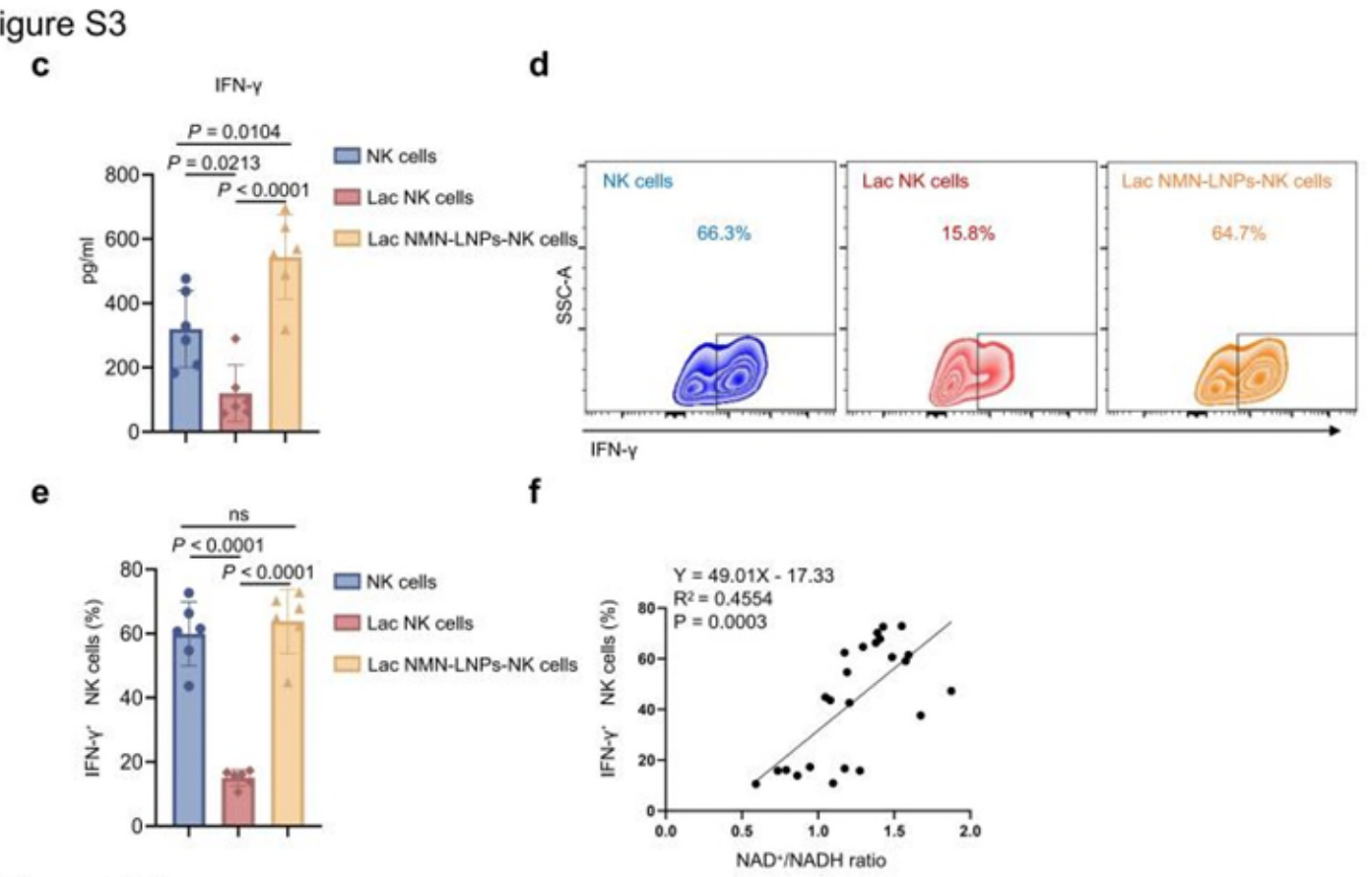

Figure S15

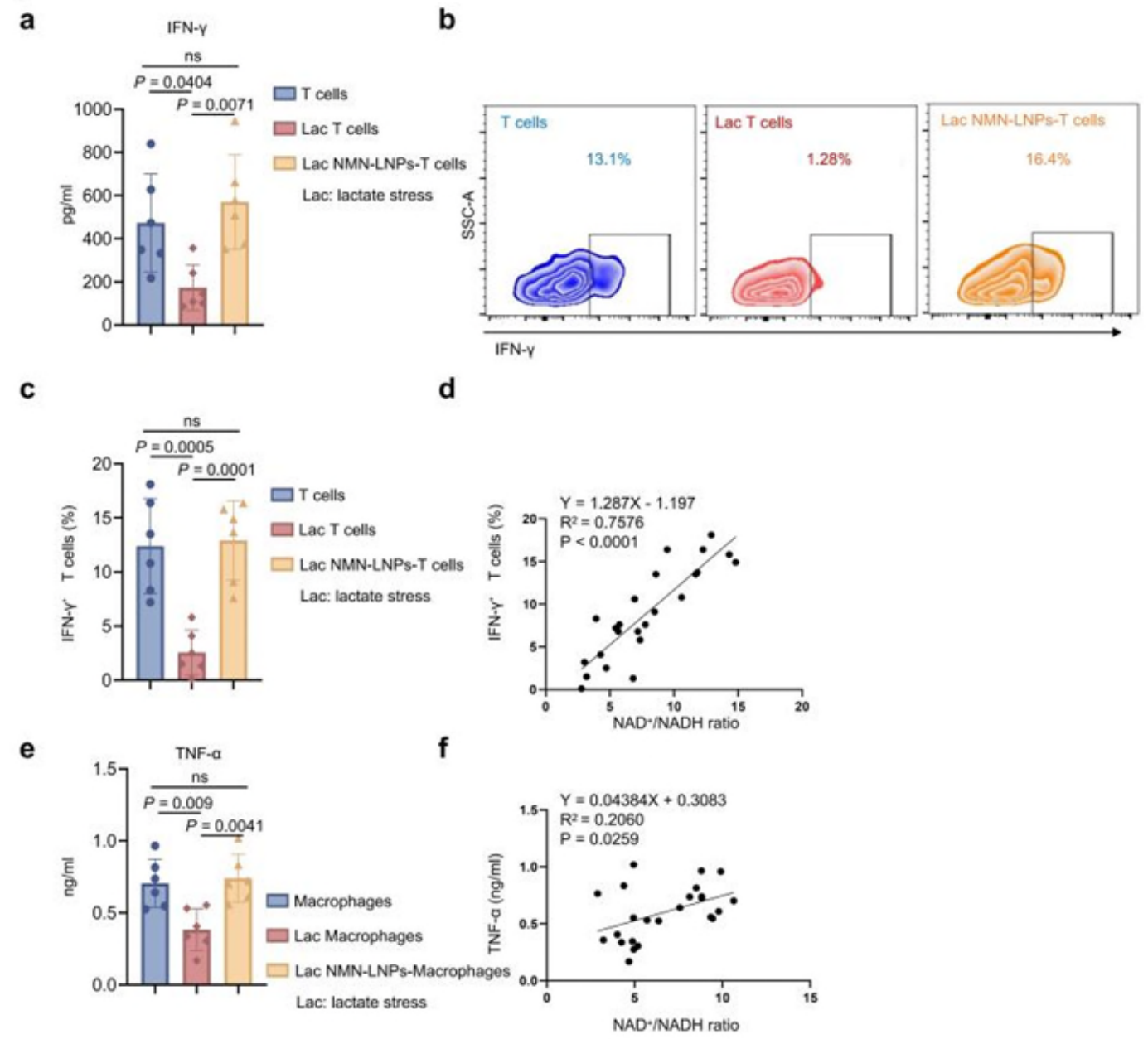

Figure S3. (c) ELISA quantification of IFN-γ concentration in the culture supernatant of NK cells under control conditions, lactate stress, or lactate stress with NMN-LNPs treatment. (d) Representative flow-cytometry plots showing intracellular IFN-γ staining in NK cells from the indicated groups. (e) Quantification of IFN-γ^+ NK cells (%) by flow cytometry. (f) Correlation analysis between intracellular NAD^+/NADH ratio and the percentage of IFN-γ^+ NK cells.

Figure S15. (a) ELISA quantification of IFN-γ concentration in the culture supernatant of T cells under control conditions, lactate stress, or lactate stress with NMN-LNP treatment. (b) Representative flow-cytometry plots showing intracellular IFN-γ staining in T cells from the indicated groups. (c) Quantification of IFN-γ^+ T cells (%) by flow cytometry. (d) Correlation analysis between the intracellular NAD^+/NADH ratio and the percentage of IFN-γ^+ T cells. (e) ELISA quantification of TNF-α concentration in the culture supernatant of macrophages under control conditions, lactate stress, or lactate stress with NMN-LNP treatment. (f) Correlation analysis between the intracellular NAD^+/NADH ratio and TNF-α concentration in macrophages. Data are presented as mean ± SD. Statistical significance was determined by one-way ANOVA with multiple-comparison correction. Correlations were assessed by Pearson correlation analysis; ns, not significant.

2. Both NMN and DCA delivery appear to be required for the observed therapeutic effects, but it remains unclear whether one component dominates the overall outcome. A clearer comparison of NMN alone, DCA alone, and the combined NMN+DCA treatment, focusing on functional readouts such as lactate production, IFN-γ production, and tumor control, would strengthen the conclusions.

Response:

We sincerely thank the reviewer for this constructive suggestion. The reviewer's comment is very helpful for clarifying how NMN-mediated intracellular support and DCA-mediated lactate depletion each contribute to the therapeutic outcome. Following this suggestion, we directly compared four groups using the same NK-cell number: NK cells, NMN-LNPs-NK cells, DCA-EMLNPs-NK cells and NMN-LNPs-DCA-EMLNPs-NK cells. This design allowed us to evaluate both the individual and combined roles of the two metabolic modules.

The results showed that neither NMN-LNPs-NK cells nor DCA-EMLNPs-NK cells alone achieved meaningful therapeutic efficacy, whereas the combined treatment produced a markedly stronger antitumour effect than either single-module treatment. In the tumour-growth assay, treatment with NMN-LNPs-NK cells or DCA-EMLNPs-NK cells exerted only mild, partial tumour inhibition, with no significant difference compared with naive NK cells. In sharp contrast, the combination group nearly halted tumour progression, demonstrating superior and indispensable cooperative activity **(Fig. S18a)**. Tumour lactate analysis further identified the distinct non-overlapping roles for each module: DCA-containing formulations were primarily responsible for tumour lactate reduction, whereas NMN-LNP-loaded NK cells alone had minimal effect on lactate levels (Fig. S18b). However, lowering lactate alone was insufficient to restore NK-cell function: treatment with DCA alone only minimally recovered IFN-γ production and cytotoxicity. Likewise, NMN alone only slightly enhanced NK-cell effector function but could not overcome the severe lactate-mediated suppression.

In marked contrast, the combination treatment simultaneously enabled both lactate clearance and robust NK-cell metabolic recovery, leading to dramatic restoration of IFN-γ secretion and the frequency of IFN-γ^{+} NK cells (Fig. S18c–e). Similarly, CD107a degranulation and perforin expression were only fully restored in the combination group, whereas single treatments remained profoundly impaired (Fig. S18f–i).Tumour immunostaining further confirmed that IFN-γ signals

were drastically enhanced only in the combination group (Fig. S18j,k). Together, these results demonstrate that NMN and DCA act in a functionally cooperative and non-redundant manner. DCA removes the external lactate barrier, while NMN sustains intracellular NK-cell status. Neither component alone is sufficient; only their combination enables full restoration of NK-cell function and optimal tumour control. These revisions have been incorporated into the Results section and Supplementary Fig. 18 legend (lines 310–326 and 1431–1458), and all corresponding changes are highlighted in colour in the revised manuscript.

Figure S18

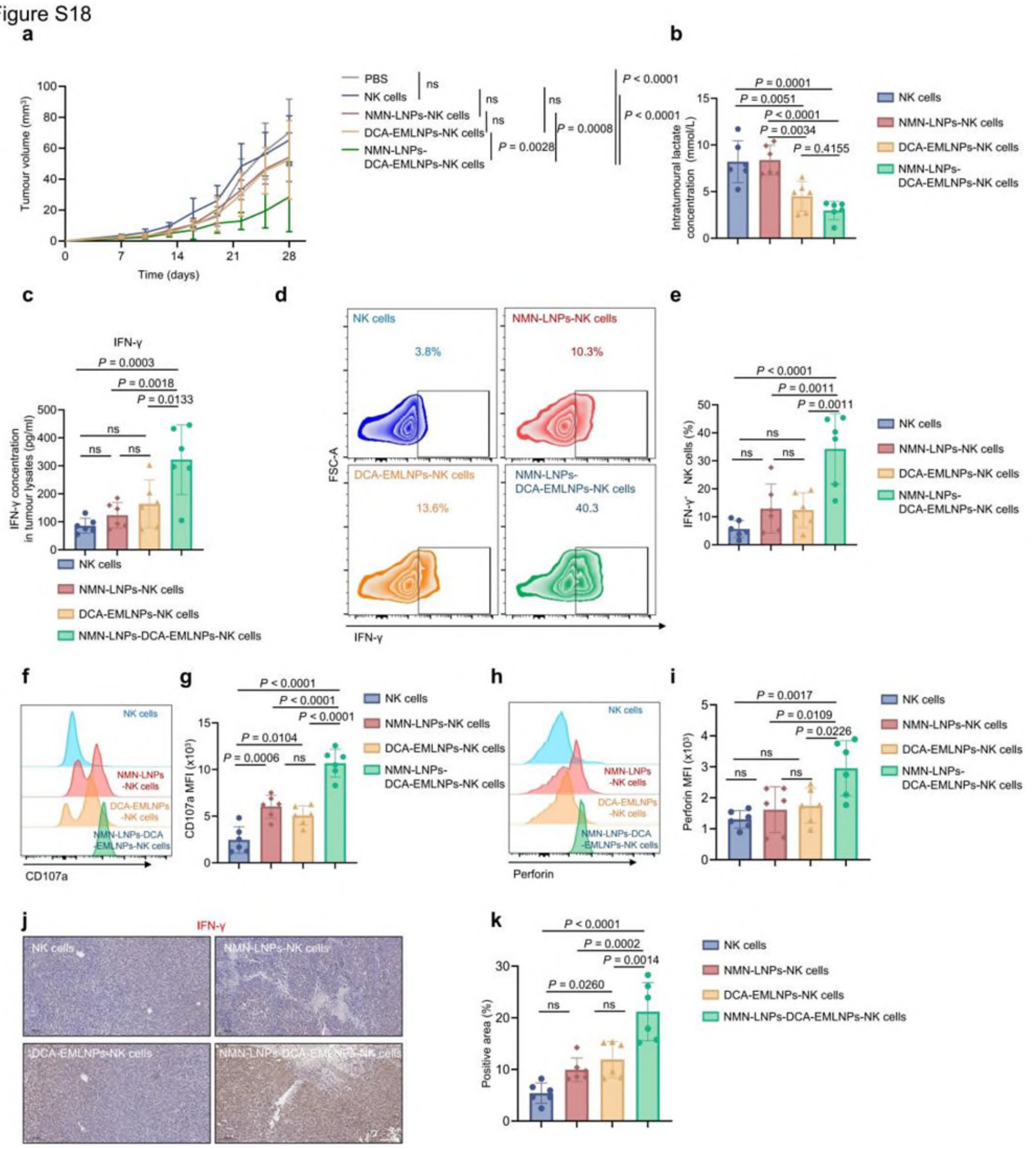

Figure S18 (a) Tumour-growth curves of HOS tumour-bearing mice treated with PBS, NK cells, NMN-LNPs-NK cells, DCA-EMLNPs-NK cells, or NMN-LNPs-DCA-EMLNPs-NK cells. Tumour volume was monitored over 28 days to compare the antitumour contribution of NMN alone, DCA alone, and combined NMN+DCA delivery. Statistical significance was determined by two-way repeated-measures ANOVA followed by Sidak's multiple-comparison test. (b) Tumour lactate concentrations in the indicated treatment groups. DCA-containing NK-cell products reduced tumour lactate levels, whereas NMN-LNP-loaded NK cells alone showed limited lactate-depleting activity. (c) ELISA quantification of IFN-γ concentration in tumour samples from mice receiving the indicated NK-cell products. (d) Representative flow-cytometry plots showing intracellular IFN-γ staining in NK cells from the indicated groups. (e) Quantification of IFN-γ^{+} NK cells (%) by flow cytometry. (f) Representative flow-cytometry histograms showing CD107a expression in NK cells from the indicated treatment groups. (g) Quantification of CD107a mean fluorescence intensity (MFI) in NK cells. (h) Representative flow-cytometry histograms showing perforin expression in NK cells from the indicated treatment groups. (i) Quantification of perforin MFI in NK cells. (j) Representative immunohistochemical staining of IFN-γ in tumour sections from mice receiving the indicated treatments. Scale bar, 200 µm. (k) Quantification of IFN-γ-positive area (%) in tumour sections. Data are presented as mean ± SD. Statistical significance in tumour-growth curves was determined by two-way repeated-measures ANOVA with multiple-comparison correction. For endpoint comparisons, statistical significance was determined by one-way ANOVA with multiple-comparison correction; ns, not significant.

Minor:

1. The manuscript would benefit from a clearer description of the different experimental groups in both the text and figures.

Response:

We sincerely thank the reviewer for this helpful suggestion and apologize for the lack of clarity in the original group descriptions. We have carefully revised the figure panels and legends to make the experimental groups easier to follow.

Specifically, we added or clarified group annotations where needed and ensured that abbreviated labels are consistently matched with their full definitions. For panels in which abbreviations were necessary due to space limitations, the corresponding full group names were provided in the figure notes or legends. These revisions improve the readability of both the main and supplementary figures without changing the experimental design or group definitions.

2.The @ usage is not wrong, but field-dependent. For Nature Comm is more appropriate to use e.g. NMN-LNPs or NMN-loaded LNPs instead of NMN@LNPs.

Response:

We thank the reviewer for this helpful suggestion. We have replaced the "@" notation throughout the manuscript, figures and figure legends to improve clarity and align the nomenclature with the style of Nature Communications.

Specifically, NMN@LNPs and DCA@EMLNPs have been revised to NMN-LNPs and DCA-EMLNPs, respectively. Related engineered cell products have also been standardized accordingly, such as NMN-LNPs-NK cells, DCA-EMLNPs-NK cells, and NMN-LNPs-DCA-EMLNPs-NK cells.

These nomenclature changes have been applied throughout the revised manuscript, figures and figure legends.

The manuscripts lacks overall clarity with respect to the dual strategy, both in describing and in experimental setup. I am therefore suggesting major revision or reject unless the authors are able to overcome this. The comments of reviewer 1 seem to be addressed.

Response:

We sincerely thank the reviewer for this important assessment. We understand the concern that the dual-compartment strategy needed to be presented more clearly, and we have focused the revision on explaining how the two modules are assembled, how each module functions, and why their combination is required. We have also revised the relevant Results subsections, figure legends and group annotations to present the platform in a more stepwise manner, moving from product assembly, to module-specific mechanism, to component-dissection and therapeutic outcome.

As described in the manuscript, ER-Guided Exo-NK is generated through a one-step ex vivo co-loading procedure in which NK cells are simultaneously incubated with NMN-LNPs and DCA-EMLNPs, followed by washing before downstream use. In this design, NMN-LNPs serve as the intracellular NAD^+-supporting module to maintain immune-cell metabolic fitness under lactate stress, whereas DCA-EMLNPs serve as the ER-guided lactate-depleting module to export DCA and reduce the extracellular lactate burden in the tumour-facing microenvironment. This explanation is intended to make clear that the strategy is not two unrelated treatments, but a coordinated dual-module cell product.

We strengthened the experimental support for the DCA module by quantifying the fate of encapsulated DCA using LC–MS/MS. We separately measured extracellular DCA, intracellular DCA, cell-free passive leakage and the unrecovered fraction after cellular loading (Fig. 4h–j). Representative chromatograms were provided to validate DCA detection in each fraction, including total loaded DCA, passive leakage, extracellular DCA in culture supernatants and intracellular DCA in NK-cell lysates (Fig. S16a–d). Extracellular DCA progressively increased over 24–96 h, whereas intracellular DCA declined reciprocally. We further assessed whether the exported DCA was functionally sufficient by measuring lactate directly in tumour tissues at 24, 48, 72 and 96 h after treatment. DCA-EMLNP-loaded NK cells reduced intratumoural lactate compared with controls, supporting local lactate suppression by the DCA module (Fig. R3a,b).

We also clarified how the two modules work within a compatible functional window. The time-aligned analysis showed that, under persistent lactate stress, NMN-mediated support preserved NK-cell degranulation through the early functional period, while ER-guided DCA export progressively increased during the same 24–72 h overlap window and was largely established by 72 h (Fig. S17f). When DCA-mediated lactate relief was included in the ER-Guided Exo-NK system, NK-cell functional activity was maintained beyond the lactate-stress-only reference window and was

accompanied by progressive tumour lactate reduction (Fig. S17g). These data help clarify that NAD^+ support and lactate clearance are temporally coordinated rather than mismatched or disconnected steps.

Finally, to determine whether one module dominated the therapeutic effect, we directly compared NK cells, NMN-LNPs-NK cells, DCA-EMLNPs-NK cells and NMN-LNPs-DCA-EMLNPs-NK cells under the same NK-cell number. The combined product achieved the strongest tumour control, whereas either single-module product produced only partial antitumour effects (Fig. S18a). DCA-containing products contributed to tumour lactate reduction, but DCA alone did not fully restore NK-cell effector function (Fig. S18b). In contrast, the combined product produced the strongest IFN-γ recovery by ELISA, intracellular flow cytometry and tumour IFN-γ staining, and also showed the greatest restoration of CD107a and perforin expression (Fig. S18c–k). Together, these revisions directly address the reviewer's concern by making the dual strategy clearer at three levels: how the two modules are assembled, how each module contributes, and why the combined NMN-LNPs-DCA-EMLNPs-NK product is required for optimal tumour lactate reduction, NK-cell functional recovery and tumour control.

REFERENCES

1. Navas, L. E. & Carnero, A. NAD+ metabolism, stemness, the immune response, and cancer. *Signal Transduct Target Ther* **6**, 2 (2021).
2. Lv, H. *et al.* NAD+ Metabolism Maintains Inducible PD-L1 Expression to Drive Tumor Immune Evasion. *Cell Metab* **33**, 110-127.e5 (2021).
3. Murray, P. J. & Wynn, T. A. Protective and pathogenic functions of macrophage subsets. *Nat Rev Immunol* **11**, 723–737 (2011).
4. Duan, Z. & Luo, Y. Targeting macrophages in cancer immunotherapy. *Signal Transduct Target Ther* **6**, 127 (2021).
